# Enhanced Photostability through Rapid Exciton Decay in Desymmetrized Cyclopentannulated Acenes with Strong Face-to-Face pi Stacking

**DOI:** 10.1021/acs.chemmater.5c02815

**Published:** 2026-01-28

**Authors:** Chad D. Cruz, Karl J. Thorley, Zachary J. Knepp, Jared Wahlstrand, Gil M. Repa, John C. Stephenson, Sean Parkin, Lisa A. Fredin, John E. Anthony, Emily G. Bittle

**Affiliations:** † 96990 National Institute of Standards and Technology, Physical Measurement Laboratory, Gaithersburg, Maryland 20899, United States; ‡ Center for Applied Energy Research, 4530 University of Kentucky, Lexington, Kentucky 40511, United States; § Department of Chemistry, 1687 Lehigh University, Bethlehem, Pennsylvania 18015, United States; ∥ Department of Chemistry, 4530 University of Kentucky, Lexington, Kentucky 40506, United States

## Abstract

The photophysics of organic semiconductors impacts their efficiency in optoelectronic devices where exciton transitions, including singlet fission, intersystem crossing and the formation of charge transfer states influence the ability to convert between bright and dark states and to dissociate into free charges. Unfortunately, photodegradation and spurious signals often confound the results of optical studies, especially of important triplet states. Here four asymmetric cyclopentannulated acenes are synthesized and studied. This system represents an extreme in photophysics achieved via molecular design to fully quench the photoluminescence and bypass triplet formation allowing for comparative studies with other highly absorbing acenes. Rapid molecular exciton decay that is unaffected by strong electronic coupling induced by the crystal packing is found. The quick return to the ground state inhibits the formation of triplets and leads to heating in the solid state. These aceacenes are photostable both in solution and as single crystals, likely because the short excited-state lifetime diminishes the chances for deleterious photoreactions. Density functional theory calculations highlight excited state twisting in the five-membered ring, indicating a key driver of rapid internal conversion.

## Graphical abstract

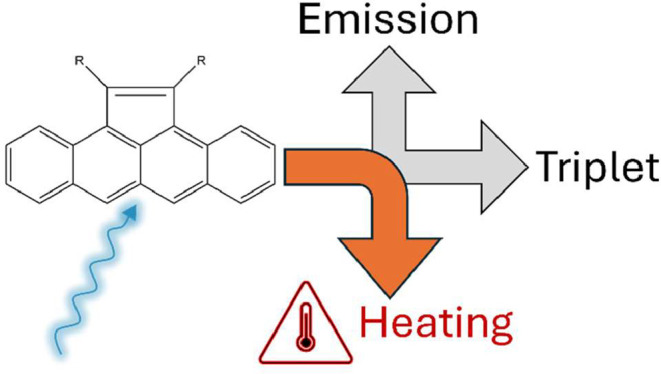

## 1 Introduction

In organic optoelectronics, molecular design is used to optimize energetic alignment and crystal packing, which are important control parameters for tuning intermolecular interactions that ultimately decide the fate of the exciton within the material.^1–3^ Of particular interest is the interconversion of singlet and triplet exciton states, which can be used to enhance the light output of light emitting diodes (LEDs) through reverse intersystem crossing (ISC) from the dark triplet to the bright singlet state^4,5^ or to enhance photovoltaic efficiency through the doubling of photon to charge conversion ratios via singlet fission (SF).^6^ The lifetimes of these processes range over orders of magnitude, with spin-allowed processes like SF generally occurring on femtosecond to picosecond time scales^7^ and ISC typically occurring on nanosecond or longer time scales.^8^ In both cases, however, the organic materials remain in an excited state for microseconds since the triplet exciton is long-lived. While long-lived excitons are desirable for long-range transport, the longer a material remains in an excited state the more chance it has to undergo photochemistry which generally leads to degradation.^9–11^ Additionally, detailed study of the exciton states is often hindered by long-lived effects, such as heating and molecular decay,^12–16^ where spectroscopic signals can interfere to give ambiguous results. To further understanding of the photophysics in device relevant molecules, it is therefore beneficial to study analogous materials with decreased rates of SF and ISC.

Polycyclic aromatic hydrocarbons are the common choice for optoelectronics applications, and those constructed with a mixture of five- and six-membered rings are well studied, in particular fullerenes^17^ and corannulenes,^18^ and materials which mimic fragments of these such as rubicene.^19^ In these cases, the five-membered rings act mostly as connectors to join other aromatic sextets together, since they feature fused benzenoid rings around the five-membered ring.^20^ If, instead, the five-membered ring contains an isolated double bond, it can have much more profound effects on the molecular optoelectronic properties. Decades ago, Garcia-Garibay and co-workers^21,22^ reported a route to cyclopenta-fused anthracenes (Figure 1c,d), which was further exploited by Plunkett and co-workers into a series of cyclopenta-fused anthracenes,^23^ tetracenes,^24^ and even pentacenes^25^ (Figure 1e). These materials were found to be potential electron acceptors for organic electronic applications, due to their favorable electrochemistry, and cyclopentannulation appears to impart significantly improved stability to the chromophores. Derivatives of these materials have found their way into donor–acceptor^26^ or ladder-^27^ conjugated polymers.

**Figure 1:**
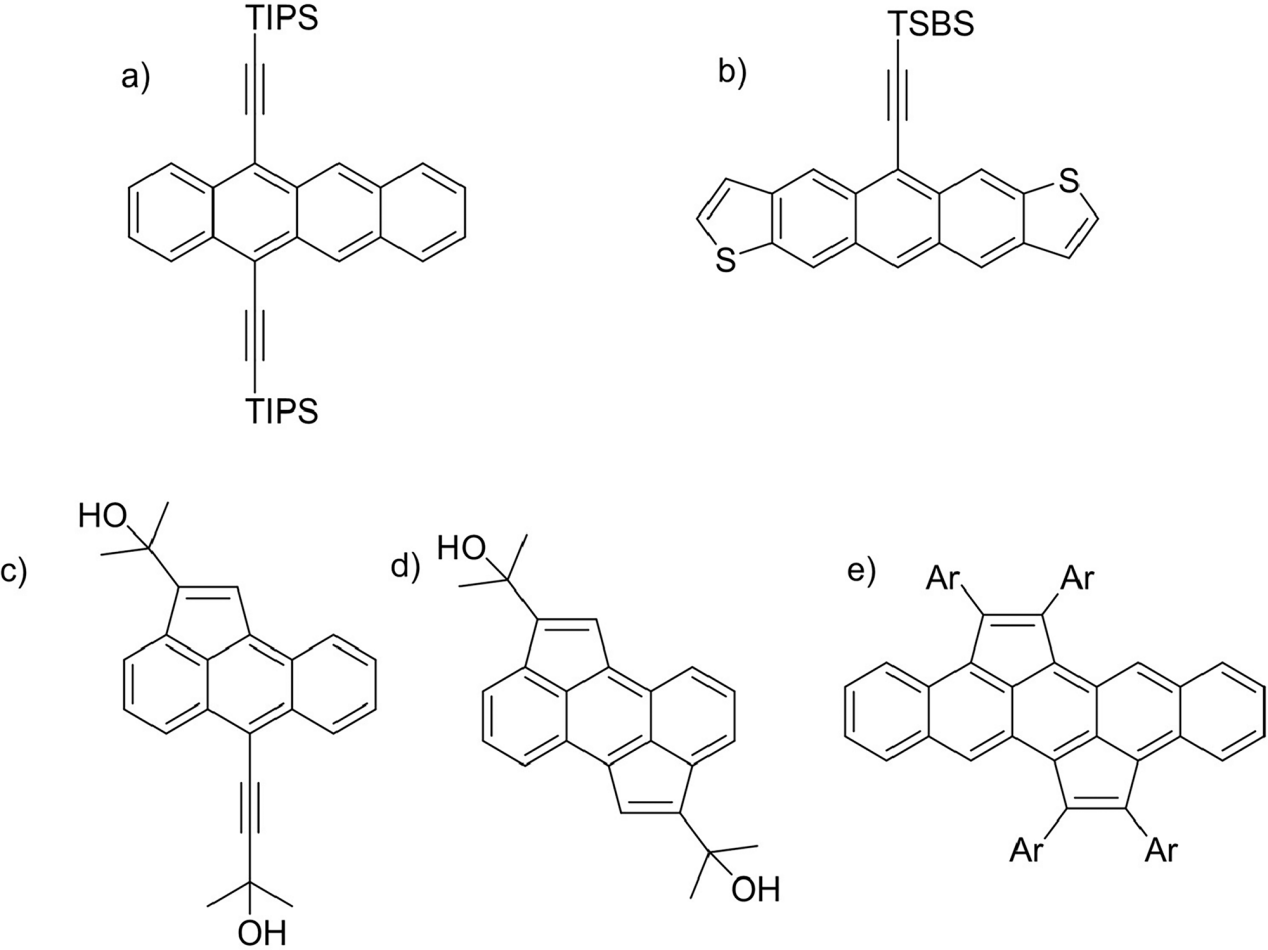
Chemical structures of relevant polycyclic aromatic hydrocarbons: (a) TIPS-tetracene, (b) desymmetrized anthradithiophene, (c) and (d) cyclopentannulated anthracenes, and (e) bis-cyclopentannulated pentacene.

Optimization of solid-state order is a key parameter across a wide swath of electronic and photonic materials and can be used to tune the exciton dynamics. One approach that we explored to induce strong π-stacking interactions in heteroacenes (anthradithiophenes) involved desymmetrizing the chromophore by adding solubilizing substituents to only one side of the molecule (Figure 1b) – leading to a flip/flop columnar stacking arrangement with strong intermolecular interactions.^28^ The use of trialkylsilyl solubilizing groups held farther from the acene core by the alkyne groups allows closer π-system packing like the desymmetrized anthradithiophenes. Furthermore, the solid-state structure can be adjusted by variation of the alkylsilyl group, such that molecules with identical molecular electronic properties can adopt different packing motifs with varying intermolecular coupling. However, few examples of small polycyclic hydrocarbons with single cyclopentannulation (desired here to yield the asymmetry required for appropriate crystal packing) have been seen in literature, and the photophysical properties of these compounds were not explored in detail.^22,29^

Here the synthesis, optical characterization and structural characterization of a series of silylethyne-functionalized monocyclopentannulated acenes are presented, demonstrating the potential for strong pairwise and long-range π–π stacking analogous to high performing electronic materials. A fast nonradiative decay that outcompetes every other relaxation channel leading to essentially no detectable emission is observed, which is unusual for strongly absorbing molecules. Pump–probe measurements find internal conversion (IC) to be the dominant deactivation pathway, ultimately leading to rapid heating in the crystalline samples. Steady-state absorption identifies a forbidden absorption band that gains oscillator strength through vibrational coupling; while distortion in the five-membered ring, as determined from time-dependent density functional theory, is the likely route to rapid energy dissipation through the side groups. The asymmetric cyclopentannulated acenes exhibit increased photostability over common polycyclic organic semiconductors, likely because the short excited-state lifetime inhibits triplet formation. Despite the predicted favorable energetic alignment of singlet and triplet energies, there is little evidence of singlet fission. We find that the triplet yield following singlet excitation is less than 1% for the aceacenes both isolated in solution and in crystalline form. Instead, the absorbed energy is converted to heat via IC to the ground state which leads to long-lived features in the transient absorption spectra.

## 2 Synthesis

Unlike the prior reported syntheses of cyclopentannulated acenes, we avoided the use of palladium-catalyzed coupling reactions^30^ for reasons of both cost and final product purity. Instead, the known double Friedel–Crafts acylation^31^ between unfunctionalized acenes and oxalyl chloride was followed, which yielded the resulting quinones in reasonable yield (Scheme 1). These acene diones proved to be stable intermediates which could be stored indefinitely under ambient conditions, making them suitable precursors to cyclopentannulated polycyclic hydrocarbons. The final products were easily and scalably produced by ethynylation following procedures we established on similar acenaphthenequinones.^32^ Yields were lower than typical silylethynyl acene synthesis, in part due to difficulty in performing the Tin­(II) deoxygenation step. Heating overnight helped improve the yield of this step toward 50%, however some diol was still observed during purification of the final products. The synthetic approach presented here should be suitable for the synthesis of a range of cyclopentannulated polycyclic hydrocarbons, the major limitation being functional group tolerance toward aluminum chloride in the Friedel–Crafts step, while a range of nucleophiles (e.g., aryllithiums or Grignard reagents) might be added to the resulting dione intermediates. The structures of four derivatives – two cyclopentannulated anthracenes (**aceAN**) and two cyclopentannulated tetracenes (**aceTN**) bearing different alkylsilyl groups were confirmed by otherwise unremarkable NMR spectra, mass spectrometry, and X-ray diffraction of suitable crystals (Scheme 1, see SI for characterization spectra).

**Scheme 1:**
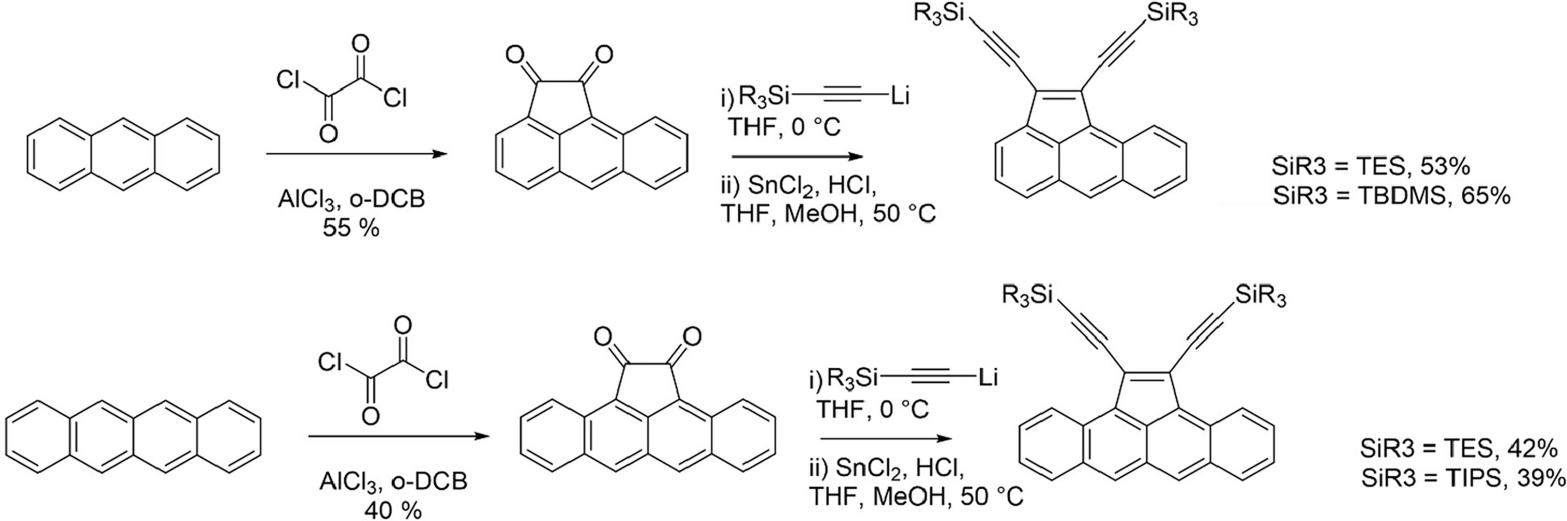
Synthesis of Cyclopentannulated Anthracene (Top) and Tetracene (Bottom) Derivatives **TES/TBDMS-aceAN** and **TES/TIPS-aceTN**

## 3 Optical Properties of the Single Molecules

The optical properties of each compound were investigated both in solution and as crystalline samples utilizing steady state UV–vis absorption measurements as well as photoluminescence (PL) measurements. The origin of the optoelectronic properties of both cyclopentannulated tetracenes was also investigated using density functional theory (DFT) and its time-dependent variant (TD-DFT). In Figure 2, the solution spectra of the cyclopentannulated derivatives are compared with their more typical acene analogs. A clear deviation from the symmetric molecules occurs upon cyclopentannulation with the most obvious difference being the color of the resulting solutions. Both solutions of **aceTN** yield a deep blue color, in stark contrast to the yellow-orange color typical of tetracene, while the colorless anthracene becomes brown upon the addition of the 5 membered ring.

**Figure 2:**
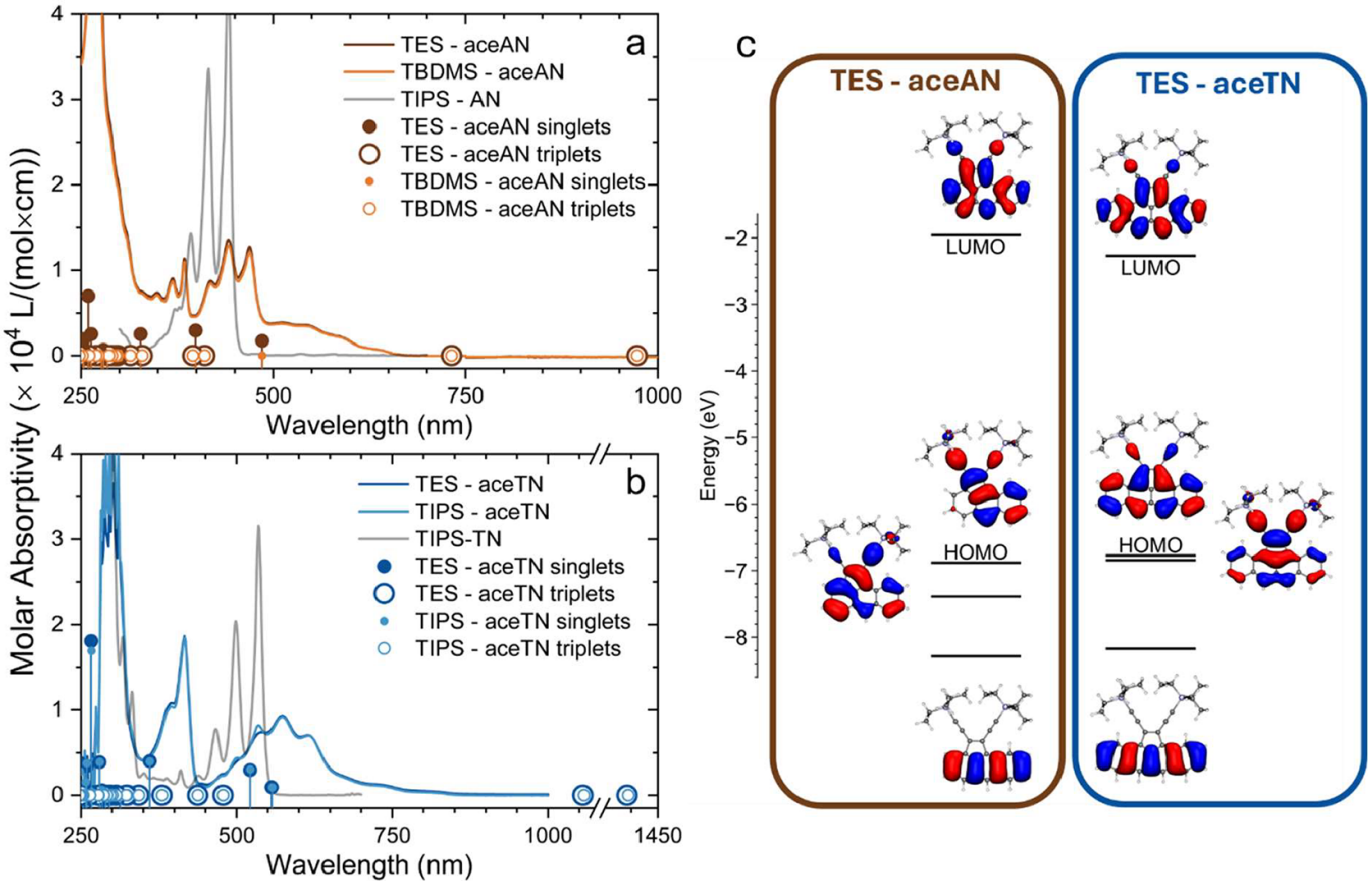
Absorbance spectra of (a) **TES-aceAN**, **TBDMS-aceAN**, and TIPS-AN in chloroform; and (b) **TES-aceTN**, **TIPS-aceTN** and TIPS-TN in toluene. Each plot also includes TD-DFT excitations with corresponding oscillator strengths (solid circles with bars are singlets, open circles are triplets). No triplet transition is found to have any oscillator strength, similar to other acenes. (c) Frontier molecular orbital (MO) diagrams of **TES-aceAN** (left) and **TES-aceTN** (right) (iso = 0.02).^37^ CAM-B3LYP-D3­(BJ)/6–311++G**/PCM­(toluene). The solution concentrations used in the absorption and quantum yield studies in this paper are small, in the 0.01 to 1 mol/m^3^ range. No significant contributions from molecular aggregates are expected (see SI).

In solution, **TES-aceAN** and **TBDMS-aceAN** (**TES-aceTN** and **TIPS-aceTN**) produce nearly identical spectra to one another (Figure 2a,b) indicating that the different alkyl chain functionalization does not alter the molecular electronic properties. However, the absorption spectra do indicate that the cyclopentadiene group is more than a stabilizing decoration on the acene backbone; instead, it also participates in the electronic transition as confirmed by our DFT calculations (Figure 2c). The emergence of additional absorption bands after cyclopentannulation is consistent with previous reports on other aceanthracenes.^33^ While the band shape in these previous reports closely resembles the spectra, the silylethynyl functionalization of **TES-aceAN** and **TBDMS-aceAN** yields absorption features red-shifted by ≈1800 cm^–1^ from other reported aceanthracene molecules indicating that the alkynyl group also contributes to the π system in agreement with the frontier MOs shown in Figure 2c. Lacking a direct literature to the acetetracenes (**TES-aceTN** and **TIPS-aceTN**) a bathochromic shift and redistribution of oscillator strength among the visible vibronic peaks is to be expected upon linear annulation.^34^ This previously reported trend aptly captures the qualitative differences between the absorbance spectra of **aceAN** and **aceTN**. TD-DFT predicts three main absorbance features in the visible region for each of the aceacenes (Table 1 and Figure 2c). For each of the three lowest energy excitations of **TES-aceAN** and **TBDMS-aceAN**, electrons are promoted into the LUMO from increasingly deeper occupied orbitals (Table 1). The first and second excitations of **TES-aceTN** and **TIPS-aceTN** occur from the nearly degenerate HOMO–1 and HOMO, respectively, to the LUMO (Table 1 and Figure 2c). The close energies of the occupied HOMO–1 and HOMO result in two convoluted absorption bands between 500 and 850 nm in the experimental spectra (Figure 2a,b), while the greater energetic offset induced by molecular asymmetry in **TES-aceAN** and **TBDMS-aceAN** results in separation of the absorption into two well-defined bands.

**Table 1:** Three Lowest Lying Singlet Excited States of Solution Phase Optimized **TES-aceAN**, **TBDMS-aceAN**, **TES-aceTN**, and **TIPS-aceTN** at CAM-B3LYP-D3­(BJ)/6–311++G** Level of Theory^a^

	energy (eV)	wavelength (nm)	orbitals	*f*	transition dipole (*x*,*y*,*z* (×10^–30^ C·m))	*D*_CT_ (Å)
**TES-aceAN**	2.5535	485.55	HOMO → LUMO	0.1738	5.444/1.131/–0.028	1.688
	3.1061	399.16	HOMO–1 → LUMO	0.2948	–4.617/4.668/–0.034	0.436
	3.7847	327.59	HOMO–2 → LUMO	0.2532	–3.142/–4.529/0.078	1.325
**TBDMS-aceAN**	2.5517	485.90	HOMO → LUMO	0.1747	–5.361/1.427/0.570	1.693
	3.1047	399.35	HOMO–1 → LUMO	0.2957	4.955/4.325/0.045	0.439
	3.7832	327.72	HOMO–2 → LUMO	0.2544	2.744/–4.707/–0.928	1.323
**TES-aceTN**	2.2291	556.20	HOMO–1 → LUMO	0.0949	–3.441/2.736/–0.043	2.390
	2.3778	521.43	HOMO → LUMO	0.2951	4.676/5.873/0.037	0.031
	3.4394	360.48	HOMO–2 → LUMO	0.4014	5.707/–4.519/0.121	0.377
TIPS-aceTN	2.2258	557.03	HOMO–1 → LUMO	0.0932	–0.405/–4.343/0.023	2.412
	2.3765	521.70	HOMO → LUMO	0.3045	–7.610/0.542/0.027	0.059
	3.4397	360.45	HOMO–2 → LUMO	0.3974	0.421/7.231/–0.123	0.376

a A chloroform polarizable continuum model (PCM) was used for the aceanthracenes while a toluene PCM was used for the acetetracenes to match experimental conditions.

The lowest energy feature in the absorbance spectra of **TES-aceAN** and **TBDMS-aceAN** is a rather weak and broadened band spanning 490 to 670 nm arising from an orbitally forbidden transition likely stemming from the poor orbital overlap between HOMO and LUMO (Figure 2c). Forbidden transitions acquiring some intensity from vibrations have been reported in other molecules containing five-membered rings.^35^ This transition bears significant cyclopentadienyl character and can be described as a charge transfer from the five-membered ring into the more delocalized LUMO. Evidence of charge transfer is further supported by the computed D_CT_ indexes^36^ of approximately 1.7 Å (Table 1). Another transition is identified between 400 to 490 nm that resembles the primary visible band in TIPS-AN although experimentally it is red-shifted from TIPS-AN by 30 nm, likely due to the larger π-network in the aceAN. This HOMO–1→LUMO excitation is described as a combination of anthracene and cyclopentadiene character due to the density of both orbitals across the whole molecular core (Figure 2c). The greater spatial overlap between the ground and excited states results in a sharper absorbance, while a rigid polycyclic structure provides fine detailed vibronic structure. The higher energy band (340 to 390 nm) is calculated to occur from HOMO–2, which is delocalized along the anthracene backbone, to the LUMO.

The absorbance spectra of **TES-aceTN** and **TIPS-aceTN** also display a weakened, broad band as the lowest energy feature spanning 660 to 850 nm. This forbidden transition is less prominent in the **aceTN**s but again has considerable cyclopentadienyl character (HOMO and LUMO in Figure 2c) and evidence of charge transfer with a computed D_CT_ of ≈2.4 Å (Table 1). The second transition is a collection of peaks between 460 to 650 nm that shows a more distinct vibronic progression. This set of peaks could be related to the visible transition in TIPS-TN although they are red-shifted ≈80 nm, likely due to the larger π-network in the aceTN. The frontier MOs show that this excitation is delocalized over the acene backbone, cyclopentadiene and alkynyl group. The greater orbital overlap between the HOMO and LUMO (Figures 2c and S18) results in a more intense absorption relative to the first forbidden transition. The third predicted transition originates from the HOMO–2 which is delocalized along the tetracene backbone. In the absorbance spectra, it appears as a stronger set of vibronic transitions between 350 to 450 nm. Experimentally, we partially resolve an additional high energy peak around 300 nm that is common to TIPS-TN and both **aceTN**s which could indicate the transition primarily involves the acene backbone.

## 4 Time-Resolved Spectroscopy of the Single Molecules

The excited states of both unsubstituted TN and AN in dilute solutions relax primarily via a combination of radiative decay and ISC,^38,39^ while TIPS-TN behaves similarly albeit with slightly longer relaxation times.^40^ In contrast, all the **aceAN** and **aceTN** derivatives have no detectable PL (see SI for details) suggesting that nonradiative processes dominate the excited state dynamics; thus, transient absorption (TA) spectroscopy is used to investigate the photophysics. Figure 3 shows the TA spectra of 1 mol/m^3^ solutions of **TES-aceAN** in degassed chloroform and **TES-aceTN** in degassed toluene excited with a 415 nm femtosecond-pump beam and probed with white light supercontinuum (SC) spanning 415–870 nm. Much like the steady state absorption spectra (Figure 2), the different functional groups on the **aceAN** and **aceTN** derivatives have no obvious impact on the photodynamics in solution producing nearly indistinguishable TA spectra (Figure S4). Because of the similarity we focus only on the subtle differences between **TES-aceAN** and **TES-aceTN**.

**Figure 3:**
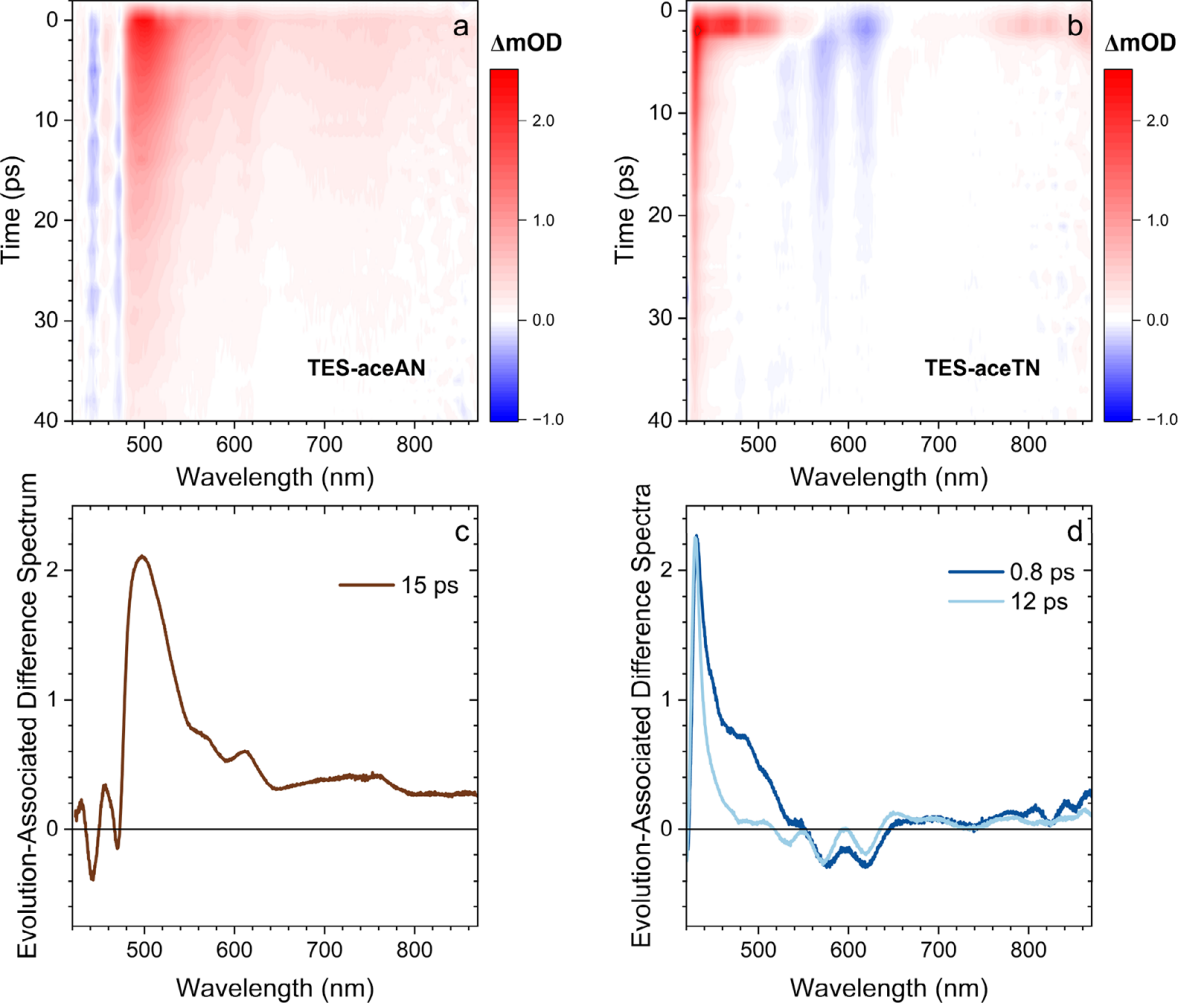
Transient absorption spectra of 1 mol/m^3^, degassed solutions of (a) **TES-aceAN** in chloroform and (b) **TES-aceTN** in toluene. Both spectra were excited with a 415 nm pump beam and were probed with white light continuum utilizing magic angle polarization between pump and probe. (c) Evolution associated difference spectrum of TES-aceAN showing that all the dynamics within the probe window decay with one time constant as determined by a target analysis. (d) Evolution associated difference spectrum of TES-aceTN showing a rapid narrowing of the SIA (dark blue) after which the entire spectrum decays with the same 12 ps time constant (light blue).

Upon photoexcitation, **TES-aceAN** shows a broad singlet induced absorption (SIA) spanning the majority of the probed wavelengths (475–870 nm) while only two negative features are observed. These negative signals are due to a ground state bleach (GSB) as they correspond to the vibronic peaks observed in the steady state spectrum. Both the SIA and GSB decay simultaneously and their collective evolution can be completely described with a single lifetime of 15 ps. This is confirmed by target analysis which simply yields a single evolution associated difference spectrum (EADS) (Figure 3c) that matches the raw TA spectrum. **TES-aceTN** also produces broad positive SIA features between 420 to 500 nm and from 675 nm to the edge of the SC. A negative collection of peaks from 550 to 650 nm is identified as a GSB as it corresponds to the vibronic peaks seen in the steady state absorption spectra. In this case, however, the dynamics are multiexponential as can be seen in the EADS (Figure 3d). The first spectral component is broad and disappears on a subpicosecond time scale revealing a longer-lived, sharp spectral component that decays in 12 ps.

Over the course of 50 ps, the SIA and GSB decay in concert without the emergence of any new spectral signatures for all **aceAN** and **aceTN** derivatives. The observed decay times are far too short to be caused by ISC in a series of molecules with no appreciable spin–orbit coupling (Tables S9–S21). Other fast nonradiative processes like SF may be caused in solution by the collision of a molecule in the excited singlet state with another molecule in the ground state, resulting in two triplets formed on each molecule. This has been reported for several acenes at concentrations typically higher (>10^3^ mol/m^3^) than in these experiments.^40,41^ Certain cases have shown that more dilute solutions can undergo SF, but the rate is limited by diffusion and thus is typically longer (many nanoseconds).^42^ At sample concentrations of 1 mol/m^3^, the diffusion-limited time between molecular collisions in toluene is 0.1 μs, so such interactions have no effect on dynamics at these short time scales.

The estimated radiative and nonradiative lifetimes show that the lack of PL comes from the fast nonradiative rate. The upper limit to the radiative rate is $\frac{1}{\tau_{\mathrm{rad}}}\approx \frac{2}{3}n^{2}\nu^{2}f$ where τ_rad_ is the radiative lifetime, *n* is the refractive index of the solvent, ν is the energy of the transition in wavenumbers and *f* is the oscillator strength (see SI for details). Estimated radiative lifetime limits for some of the low-lying electronic states are ≈15 ns for the aceanthracenes and ≈30 ns for the acetetracenes. The nonradiative lifetime, τ_NR_, can be estimated from the measured photoluminescence quantum yield (PLQY) using the relation τ_NR_ = τ_rad_ × PLQY. For the electronic states excited in the TA experiments, PLQY ≤ 2 × 10^–4^ for all the aceacenes (Table S2). The resultant nonradiative rates can be estimated to be in the range ≈ 0.3 to 2.0 ps, depending on the molecule and electronic state excited. Those estimated numbers are smaller than the observed values in the range 12–15 ps from TA measurements. This discrepancy is not entirely surprising as large deviations from the Strickler–Berg relation^43^ are commonplace for molecules that undergo conformational changes upon excitation or have forbidden transitions like the aceacenes studied here.^44–50^ Critically, both estimates support rapid depopulation of the initially excited singlet state. To conclusively rule out triplet formation, we performed sensitization experiments which show that triplet species would be detectable in our spectral window as there is a strong triplet-induced absorption spanning 460–530 nm yielding a triplet lifetime of ≈1 μs (Figure S5). Without the presence of the triplet sensitizer, the triplet yield following singlet excitation for the aceacenes is less than 1% (see SI for details). The lack of any triplet signatures and the very fast decay times confirm that internal conversion is the primary decay pathway for each of the aceacenes in solution.

The one notable dynamic difference between **aceAN** and **aceTN** derivatives is the subpicosecond component present in the acetetracenes. This likely corresponds to internal conversion from higher lying excited states to the lowest excited state. The 415 nm pump corresponds to a more energetic excited state in the acetetracenes; whereas this excitation only populates the lowest energy allowed band in the aceanthracenes. Despite the highly efficient internal conversion in these molecules, intermolecular interactions in the crystalline samples could still lead to rich excited state dynamics.^51–53^

## 5 Photochemical Stability

A particularly striking feature of the cyclopentannulated acenes is their enhanced photochemical stability compared to their noncyclopentannulated polyaromatic analogs. This is evidenced by changes in UV–vis absorption spectra. For instance, we observe that **TIPS-aceTN** in toluene exposed to air but shielded from light shows no sign of decomposition over months, whereas the same concentration of symmetrically functionalized trimethylsilylethynyl tetracene (TMS-TN) shows ≈30% decomposition in 1 week. To quantify this, dilute, aerated solutions of **TES-aceAN**, **TBDMS-aceAN**, **TIPS-aceTN** and TMS-TN were optically excited at low light intensity to various electronic states. The decrease in absorption gives the quantum yield measured for photodecomposition, PDQY, which is a measure of molecules destroyed per photons absorbed (see SI for details). For TMS-TN in 2-propanol (iPA) excited in the red (527 nm, lowest energy transition) PDQY = 2 × 10^–4^. In contrast, **TIPS-aceTN** in iPA excited in the red (572 nm, next to lowest transition) had PDQY < 1.5 × 10^–6^. At the lowest energy transition (730 nm) in toluene, **TIPS-aceTN** had PDQY < 3 × 10^–7^, and the lowest transition in **TES-aceAN** (547 nm) had PDQY < 3 × 10^–7^. Comparably small PDQY were also observed for **TES-aceAN**, **TBDMS-aceAN**, and **TIPS-aceTN** when pumped to the next two lowest visible wavelength transitions. However, the cyclopentannulated acenes do decompose readily when excited in the UV. For instance, **TES-aceAN** in iPA pumped at 269 nm gave PDQY ≈ 10^–3^. There are similar limits on the PDQY in the solid state, i.e., PDQY < 3 × 10^–7^ for visible transitions (see SI). Or phrased more qualitatively, in typical experiments done in absence of oxygen, solid TMS-TN decomposed in ≈10 min of irradiation whereas at comparable excitation in **TIPS-aceTN** showed no decomposition in ≈10 h.

## 6 Solid-State Properties

A benefit of utilizing silylethynyl substituents is the ability to synthesize derivatives which are electronically identical to one another as isolated molecules (e.g., in solution) but possess different side chain volume causing them to pack differently in the solid state. For **aceTN**, both triethylsilyl (TES) and triisopropylsilyl (TIPS) derivatives were synthesized and suitable crystals were grown from slow cooling of saturated solutions. The smaller **aceAN** required smaller side chains with the tertbutyldimethylsilyl (TBDMS) derivative yielding X-ray suitable crystals, while crystals of the **TES-aceAN** did not produce sufficient quality diffraction to allow solid-state structure determination.

In each of the crystal structures determined by X-ray diffraction (Figure 4), the asymmetric substitution of the silylethynyl groups to only one edge of the acene structure results in alternation of molecular orientation within π-stacks in the crystal. Edge-to-face interactions observed in desymmetrized anthradithiophenes are notably absent, with face-to-face π-stacking the preferred interaction between polycyclic hydrocarbon cores. Plane-to-plane π-stacking distances are between 3.3 and 3.4 Å, with pairwise binding energies computed up to 1.4 eV (30 kcal/mol), indicating a strong affinity for this face-to-face alignment (Tables S4 and S5). Additionally, each of these aceacenes possesses at least one dimer with a cofacial slip stack arrangement (Figure S20) that has been identified as a favorable motif for efficient SF in other molecules.^54–56^ In all cases, the asymmetric substitution pattern of the aceacenes results in alternating placement of the side chains along the stack, a motif that might be exploited in other materials to generate unique solid state packing structures for a range of optoelectronic applications.

**Figure 4:**
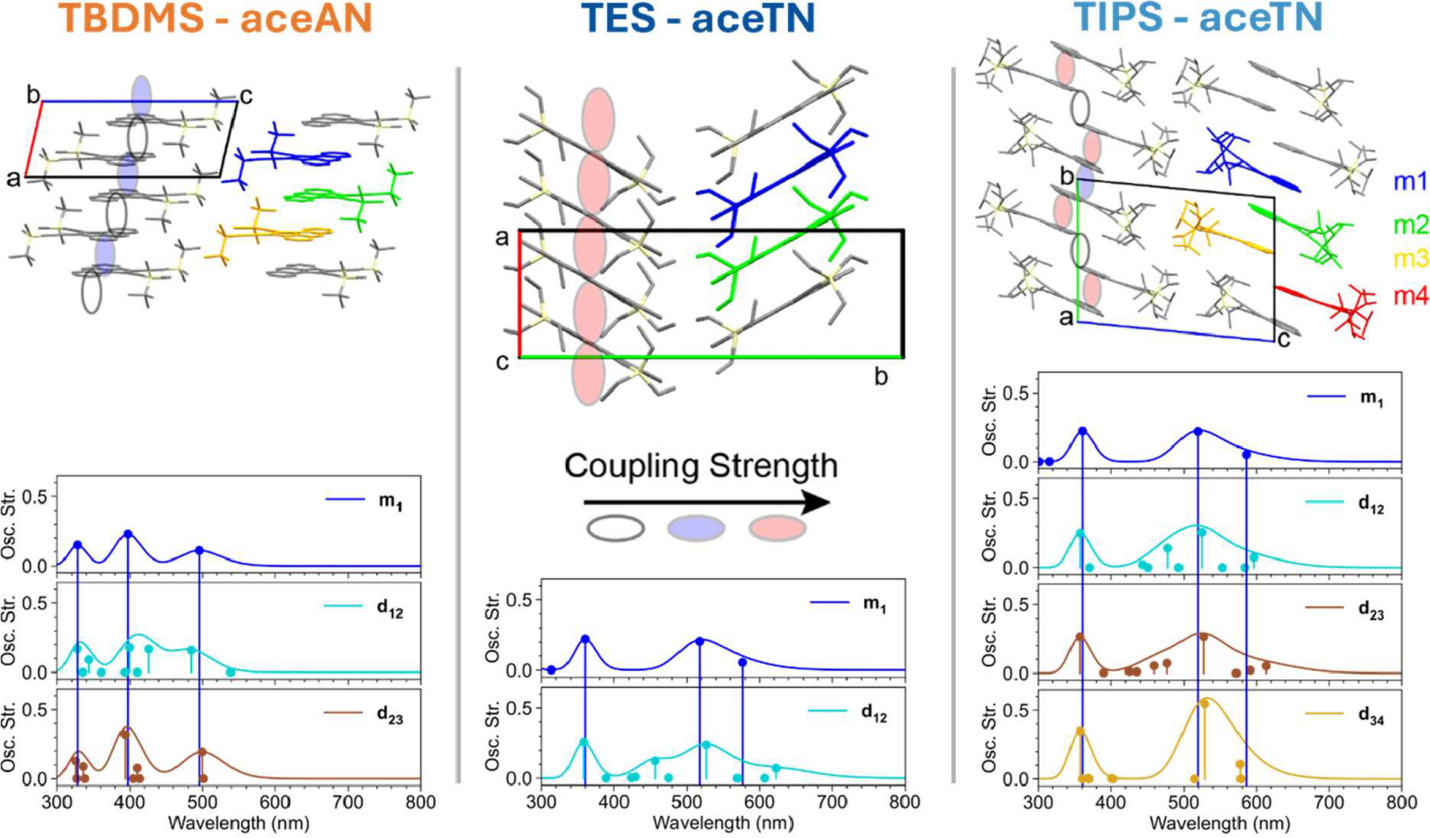
X-ray packing of derivatives **TBDMS-aceAN** (top, left), **TES-aceTN** (top, middle), and **TIPS-aceTN** (top, right). Ovals indicate relative computed electronic coupling strengths between LUMO orbitals of adjacent molecules. Monomers m1-m4 used in Table 2 are highlighted as different colors. Monomer and dimer TD-DFT predicted excitations at CAM-B3LYP-D3­(BJ)/6–311++G** level of theory for **TBDMS-aceAN** (bottom, left), **TES-aceTN** (bottom, middle), and **TIPS-aceTN** (bottom, right). Geometries are extracted from the measured crystal structures.

The change in side chain between **TES-aceTN** and **TIPS-aceTN** provides two different packing schemes. **TES-aceTN** forms 1-dimensional π-stacks with a herringbone arrangement between each of these stacks. Electronic coupling (Table 2) between adjacent molecules in the stacks was calculated using density functional theory (DFT) and is large; the LUMO–LUMO overlap results in transfer integrals of ≈249 meV whereas typical organic semiconductor transfer integrals are under 100 meV.^57^ Increasing the side chain size to the TIPS substituents maintains π-stacking interactions, but these occur in clusters of four molecules rather than the continuous 1-dimensional stacking of **TES-aceTN**. This kind of arrangement is reminiscent of recently published thienoacenes where strong coupling of parallel aligned isolated molecular pairs was investigated for singlet fission-induced quantum information applications.^58^ For **TIPS-aceTN**, any excited triplet pairs would be confined within a stack of four molecules which have electronic couplings of ≈140 meV, while coupling to adjacent quartets is much weaker at ≈50 meV. While the electronic couplings between frontier molecular orbitals are large, the computed excitation energies of molecular pairs show little variation from the isolated molecules, suggesting that some absorption band broadening should be expected in the solid state with a lack of any dramatic spectral shifts.

**Table 2:** Absolute Values of the Effective Electronic Coupling (*t*_ab_) Strengths between the HOMO (LUMO) Orbitals of Adjacent Molecules Extracted from **TBDMS-aceAN**, **TES-aceTN,** and **TIPS-aceTN** at CAM-B3LYP-D3­(BJ)/6-311++G** Level of Theory^a^

	|*t*_12_| [meV]	|*t*_23_| [meV]	|*t*_34_| [meV]
TBDMS-aceAN	183 (142)	90 (15)	
**TES-aceTN**	32 (249)		
**TIPS-aceTN**	140 (85)	78 (218)	86 (50)

a See Figure 4 for monomer numbering scheme.

## 7 Solid-State Absorption

Of the two **aceAN**s explored, only **TES-aceAN** formed single, square-shaped crystals (Figure S3) that were large enough to be probed optically. In contrast **TBDMS-aceAN** formed rough, polycrystalline films that prevented clean optical characterization. Figure 5a compares molecular absorption to polarized crystalline absorption along orthogonal optical axes. Along both optical axes the absorption is broad with multiple overlapping features. Along the first optical axis (Figure 5a, dotted line), absorption is enhanced between 380 to 420 nm, while that feature seems to be suppressed along the orthogonal axis (Figure 5a, dashed line). The biggest change from solution to solid appears to be a redistribution of oscillator strength among the transitions between 380 to 690 nm. On the other hand, polycrystalline samples of **TBDMS-aceAN** appear as a broadened version of the molecular spectrum where all the vibronic resolution has been lost (Figure 5c). The apparent increase of absorption from 700 to 750 nm is likely an artifact resulting from the strongly scattering sample and the inability of recording a reflectance spectrum from it. The lack of new spectral features is supported by the TD-DFT absorption spectra of the crystalline dimers (Figures 5 and S24–S26). No new absorption bands or types of absorption features arise from the interaction between monomers in the crystal structures. Instead, the nearest-neighbor transition orbitals (Figures S24–S26) look like large molecular orbitals, delocalized across both molecules. The symmetry of the different pairwise stacking gives rise to slightly different splitting of the solution transitions (Figure 5), which probably leads to the overall broadening of the experimental spectra whose computational equivalent would be the sum of all nearest-neighbor pairs. Interestingly, the molecular dipoles are antiparallel in all the crystal structures with the five-membered rings aligning opposite in each nearest-neighbor pair along the packing directions, cumulating in very small dipole moments of each pair. This means that the delocalization across the crystalline dimers is perpendicular to very weak dipole moments leading to small splitting of solution transitions in each nearest-neighbor pair.

**Figure 5:**
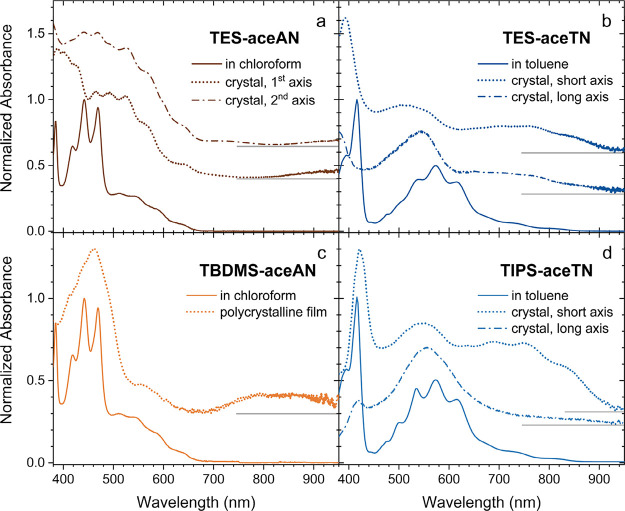
Normalized absorbance spectra comparing solid samples to solutions for **TES-aceAN** (a), **TES-aceTN** (b), **TBDMS-aceAN** (c), and **TIPS-aceTN** (d). Polarized absorbance spectra for **TES-aceAN** are shown along orthogonal optical axes which did not correspond to a well-defined crystal growth direction. The optical axes for both **aceTN** derivatives coincide with obvious crystal growth directions, and the polarized spectra are defined with respect to them. The spectra have been vertically offset for clarity. The baseline for each offset spectrum is shown by the gray solid line on the long wavelength side of the plot.

Crystals of **TES-aceTN** grow primarily as long needles with obvious long and short axes (Figure S3). The optical axes approximately correspond to these two crystal growth directions, and the polarized absorption shown in Figure 5b is distinguished as polarized along the long and short crystal axes. Light polarized along the short axis of the crystal yields a strongly absorptive feature around 390 nm and two broad features from 450 to 600 nm and 620 to 900 nm. The long axis polarized spectrum also shows these features, but the 450 to 600 nm feature becomes more intense. The chief difference between the polarized spectra lies in the 390 nm peak which is absent for the long axis polarized spectrum. Unfortunately, our white light continuum does not extend to shorter wavelengths, so we were unable to fully resolve the peak at 390 nm or look for the possibility of a shoulder like that seen in solution. Compared to **TES-aceTN** in solution a blue shift of ≈30 nm is observed in the crystal for both polarizations. The crystal structure determined for **TES-aceTN** shows that 1-D π stacks are formed with very strong calculated electronic coupling between the faces of adjacent molecules. Hypsochromic shifts can often be found in molecular solids that have a face-to-face π stacking motif between chromophores which might explain the observed blue shift.^59^ We do note, however, that determining H- or J-aggregation simply based on spectral shifts between the solution and solid has not proven to be the most reliable means of characterization.^60,61^

Crystalline **TIPS-aceTN** forms large rectangular crystals (Figure S3) which also have the optical axes approximately aligned with the long and short axes of the crystal. Polarized absorption measurements show a strong anisotropy along the crystal axes. Like **TES-aceTN**, the short axis polarized spectrum favors the high energy peak, this time centered at 420 nm which is shifted to the red ≈5 nm compared to solution (Figure 5d). A blue-shifted (≈25 nm), featureless peak centered at 550 nm is observed followed by a broad, vibrationally resolved feature from 650 to 930 nm. These long wavelength vibronic peaks are slightly red-shifted (≈15 nm) compared to the solution revealing a complicated solution-to-solid absorption change with two features shifting red (420 and 650 nm to 930 nm) and one feature shifting blue (550 nm). Probing the long crystal axis results in a strong decrease of the 420 nm peak while the region from 610 to 930 nm shows no detectable absorption. Compared to **TES-aceTN**, the forbidden transitions (650–930 nm) are more intense and the vibronic progression is better resolved. DFT shows that this transition has significant cyclopentadiene character. In the crystal structures, **TIPS-aceTN** has a larger dihedral angle (≈ 6°) between the silylethynyl groups than **TES-aceTN** (≈ 0°) indicating that cycopentadiene is slightly twisted in **TIPS-aceTN** enabling the forbidden transition to gain more intensity. The large anisotropy in the absorption spectra of **TIPS-aceTN** is likely indicative of the common molecular axis arrangement between 1D chromophore stacks along the *b* axis (Figure 4) and is evident in the high anisotropy of the transition dipole calculated for the HOMO to LUMO (S_1_) transition (Table 1). In contrast, **TES-aceTN** shows lower anisotropy in polarized absorption, transition dipole, and molecular stacking. This is reflective of the herringbone packing observed in single crystal X-ray diffraction, where the larger variation of molecular transition dipoles relative to the substrate surface results in more consistent absorption with polarization direction.

## 8 Time-Resolved Spectroscopy and Heating Analysis in the Solid State

Crystalline samples do not exhibit any detectable PL, similar to the isolated molecules, necessitating TA measurements for elucidating their photophysics. Crystals between 0.8 and 1 μm thick were grown on fused quartz substrates and placed into a nitrogen purge cell fitted with optical windows. The crystals are highly anisotropic, and the TA spectra show different spectral features depending on the polarization of the probe beam; however, the dynamics are largely independent of polarization. At first glance, the TA spectra are complex with many overlapping short- and long-lived features (Figure 6). Initially three regions with positive differential signals can be identified and assigned to SIA. Two broad negative features caused by a GSB are observed which are centered ≈570 and ≈780 nm (≈550 and ≈770 nm) for **TES-aceTN** (**TIPS-aceTN**). The lowest energy absorptive feature from 750 to 880 nm is better resolved in the crystalline samples. These features are indeed a GSB as confirmed by pumping at 575 and 830 nm and observing the same TA spectra (Figures S8 and S9). The initial GSB decay in the solid is biexponential having approximately 2 and 10 ps time constants (see Figure S7), whereas in solution it is 12–15 ps (Figure 3). Strikingly, after an initial rapid decay over the first 100 ps, certain spectral regions shift and grow back in (*t* > 540 ps). The reemergent features last for microseconds before giving way to the ground state. The long time TA spectral changes resemble the triplet absorption for TIPS-aceTN in solution as determined by triplet sensitization experiments (Figure S5); however, care must be taken when interpreting pump–probe dynamics of solid-state materials, because pump-induced heating can introduce spurious signals. The predominance of internal conversion found in the molecular spectroscopy further raises the possibility that rapid crystal heating could yield artifacts unrelated to the intrinsic photophysics, as has been previously observed in other OSCs containing five membered rings.^15^

**Figure 6:**
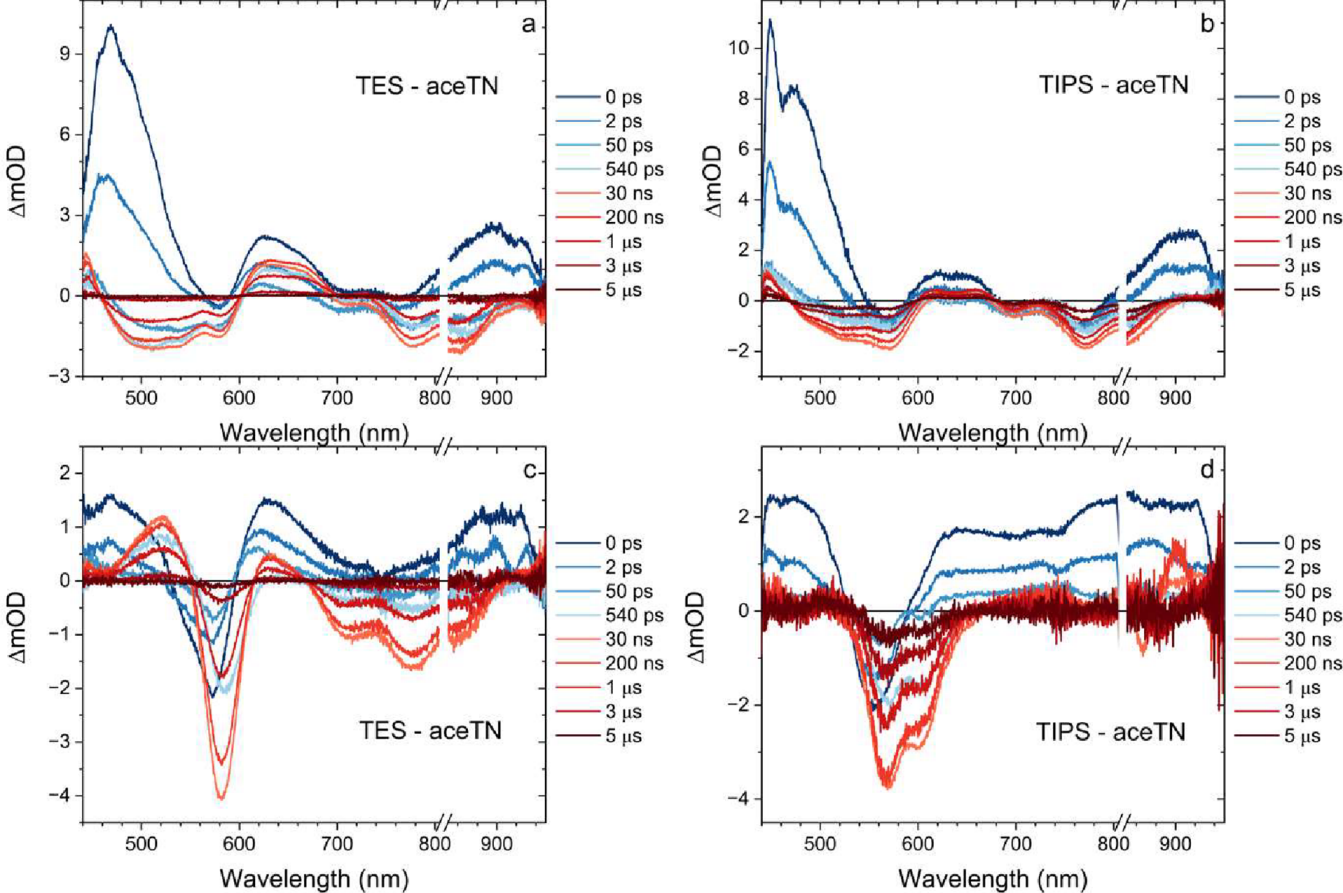
Transient absorption spectra of (a, c) **TES-aceTN** and (b, d) **TIPS-aceTN** crystals measured with both the 415 nm pump and white light continuum probe polarized along the long axis (a, b) and short axis (c, d) of each respective crystal. The break in the wavelength axis is to remove second order scattering of the pump beam from the spectra.

Optimally, one would perform a transient grating experiment to characterize thermalization in the crystalline samples.^62,63^ Given our small crystals combined with the use of a high numerical aperture objective, adding a second pump beam at a different angle to generate a grating for this experiment is not possible. Typically, identifying laser heating artifacts in TA experiments involves two strategies: (1) taking the steady-state absorbance spectrum of the heated sample versus a room temperature sample^13,15,16^ and (2) performing the TA experiment on a more thermally conductive substrate like sapphire.^12,64^ The first method seeks to generate a difference spectrum that is purely thermal by subtracting the steady-state absorbance at room temperature from one taken at an elevated temperature. This technique captures the features caused by laser heating assuming that the sample can be approximately described by a uniform temperature increase. Oftentimes this is combined with the second strategy using time-resolved comparisons on glass versus sapphire. The larger thermal conductivity of sapphire should cause any heat-related artifacts to decay faster while the pure molecular response will remain constant on each substrate. Such substrate dependent dynamics are easily implemented on thin polycrystalline and amorphous films, but the small residual birefringence present in C-plane sapphire complicates the determination of optical axes when birefringent crystals, like the ones in this work, are grown on it. Furthermore, the relatively thick aceacene crystals will be much less sensitive to differences in substrate conductivity than thinner films (Figure S15).

We heated the crystals within the purge cell and recorded steady-state absorption spectra. Because of the nonuniform crystal thickness, measuring a repeatable thermal spectrum required carefully translating the crystal to compensate for the thermal expansion of the sample mount. The observed TA signals shared a close resemblance to thermal changes in regions near the GSB features for Δ*T* ≈ 30–60 K (Figure S14). However, the long-lived induced absorption where we expected the triplet signal to appear could not be captured by the thermal spectra at any temperature. Because the usual techniques for thermal artifact identification are not ideal for optically thick crystals, we turned to modeling the heat flow (see Figures S15–S18 for details). From the known laser excitation fluence and the known absorption of the sample, the calculation of the maximum possible temperature rise Δ*T*_max_ throughout the sample is readily determined. If that number is very small, TA features caused by heating will also be small. Δ*T*_max_ varies depending on excitation wavelength, but Δ*T*_max_ ≈ 13 K is typical. The peak temperature is found at the laser entry surface and is much less at a depth of 0.7 μm into 0.8 μm thick crystals. From the heat flow equation, Δ*T*(*x,t*), the temperature as a function of time, *t*, and depth, *x*, in the sample after initial excitation, can be calculated. The observed decay of the TA spectra with long time constants (0.5–2.2 μs) is entirely congruent with the decay time expected for heat dissipation in these crystals rather than a long-lived exciton species. The TA spectra in Figure 6 are then best described by two different regimes. The first captures the intrinsic, ultrafast IC back to the ground state, but unlike the solution measurements, the absorbed energy is dissipated as heat into the crystal. This leads to the second regime containing long-lived thermal signatures that could easily be mistaken for triplets.

## 9 Discussion

Photophysical measurements of organic semiconductors are crucial for determining the formation, lifetimes, and transport length of excitons. These properties are essential to optoelectronic applications. In organic photovoltaics, common practice is to tune the bulk structure to the exciton transport length to ensure excitons reach dissociation sites before decaying. In both photovoltaics and LEDs managing the ratios of singlets and triplets, as well as the formation of charge transfer states, affects device efficiency. Looking ahead, the potential use of excitons for quantum memory requires long lifetimes for storage. The aceacenes we show here can be used to identify artifacts in common high intensity and ultrafast photophysics experiments, such as transient absorption and nonlinear measurements of single crystals. In comparison studies with other high-performance materials, heating effects, low photodegradation, and polarized measurements can be demonstrated without triplet-related signals. Here, we examine mechanisms that result in bypassing the triplet exciton and the related photostability, as well as the effects and limitations of heating measurements in crystalline samples.

### 9.1 Fast Internal Conversion and Photostability

The aceacenes strongly favor internal conversion over radiative emission, leading to very low photoluminescence (PL) and rapid excited state decay in dilute solutions despite having accessible triplet states. Compared to similar strongly absorbing acenes such as 9,10 – dialkynylanthracene (PLQY = 0.87),^21^ anthracene (PLQY = 0.30),^65^ tetracene (PLQY = 0.17),^66^ or TMS-TN (PLQY = 0.60), the low PLQY in the aceacenes appears to be an outlier. However, asymmetric and symmetric aceanthrylene molecules explored previously^21,22^ were also reported to have no detectable emission. The mechanism behind the lack of PL was not fully explored in the literature but attempts to indirectly measure triplet production with ^1^O_2_ sensitization suggested that ISC was not a primary contributor to the nonradiative decay. Other reports of polycyclic aromatic hydrocarbons with five-membered rings have similarly low PLQYs with calorimetric experiments suggesting that IC is a common nonradiative pathway for these molecules.^15,67–69^ In the absence of heavy atoms or extensive CT states, ISC would be expected to be a long-time scale process unable to entirely quench the emission on these picosecond time scales.^8^ DFT shows that there is no appreciable spin–orbit coupling in any of these aceacenes (Tables S9–S21); thus, rapid triplet generation is not expected. Furthermore, the data show that triplet formation from ISC is completely inconsequential. In solution, the fraction of excited singlets that form triplets is less than 1.4 × 10^–3^. With the assumption that the triplet absorption cross section in the crystal should be similar to the molecular cross section, we calculate the crystalline triplet yield to be less than 1.7 × 10^–3^ (see SI for details). The rapid IC preventing appreciable triplet generation may be caused by a distortion in the five-membered ring identified in DFT calculations. In particular, the symmetry of the aromatic five-membered ring leads to the electronic density bleeding onto the side groups, seen in all the orbitals (Figure 2c). This density on the first bond of the side groups means that the vibrational and rotational degrees of freedom of the side groups can directly disrupt the electron density on the aceAN and aceTN cores. We speculate that this tying of the side group motions to the aromatic density provides many rapid IC paths back to the ground state.

The photophysics remains unchanged in the solid state, indicating that the exciton properties and IC mechanism are not significantly influenced by neighboring molecules. This is despite strong π–π interactions like those in top-performing organic semiconductor structures. This consistency in physics between solution and solid state simplifies measurement and interpretation.

The increased photochemical stability of aceacenes compared to other acenes correlates with findings from other studies, which indicate that the presence of excitons is associated with photodegradation. Both singlet and triplet excitons can trigger unfavorable chemical changes. In functionalized tetracene, the formation of the triplet pair state has been linked to increased degradation through endoperoxide formation and photodimerization.^11^ The thermodynamics involving excitons and triplet oxygen influence the photodegradation process,^9^ with materials exhibiting low-energy exciton states showing better stability. The short exciton lifetimes, coupled with their low-energy ground states, likely explain the impressive material stability of aceacenes.

### 9.2 Heating in Crystalline Samples

In TA of the crystalline samples, microsecond features are observed that could easily be misinterpreted as triplet species since the heated steady-state absorption spectra partially reproduce the thermal artifacts. The lack of a uniform temperature distribution through the crystal, however, means that none of the heated steady-state spectra should be expected to accurately capture the thermal artifacts as Δ*T*(*x, t*) depends strongly on the depth position in the sample. Since the sample is not optically thin, probe wavelengths with the strongest absorptivity do not penetrate much farther than the front surface yielding the highest Δ*T*(*x, t*), while wavelengths of lower absorbance have an array of values for Δ*T*(*x, t*). Even if the sample were optically thin, Δ*T*(*x, t* > 0) varies dramatically throughout the sample due to heat flow into the substrate. Therefore, it is not surprising that no experimental TA spectrum matches any particular thermal spectrum. Generally, this means that caution must be taken when comparing TA spectra with simulated thermal artifacts generated from steady-state absorbance alone as features not captured by the thermal artifact spectrum still may not represent exciton species. Indeed, excitation of samples with rapid IC that generate heat without interference from long-lived exciton features, provide a clean TA measurement of the nonuniform sample heating. An exciting extension of this work using materials with rapid IC could help to uncover reliable methods to remove heating signatures from TA data through careful sample selection, measurement, and modeling.

## 10 Conclusions

Understanding the formation and properties of dark, long-lived triplet excitons is crucial for improving optoelectronic device performance and exploring quantum technology applications of organic materials. However, the photophysics of the triplet state are often obscured by material photodegradation and heating effects. Cyclopentannulation significantly enhances internal conversion of singlet exciton states, preventing triplet formation, boosting photochemical stability, and yielding a strong photothermal response compared to similar acenes. These molecules have stronger π–π interactions in their single crystal structures than previously reported aceacenes, achieved by the inclusion of trialkylsilylethynyl groups on only one side of the molecule. Despite strong coupling and long-range order, there is little change in the photophysical properties in the solid state compared to dilute solutions. Poor HOMO–LUMO overlap in the low-energy excited states contributes to the absence of PL, and rapid energy dissipation facilitated by the five-membered ring and solubilizing group motions outpaces triplet formation.

This study sheds light on how heat dissipation, triplet formation, and stability are interconnected in common donor structures. These aceacenes are structurally similar to high-performance organic semiconductors and, therefore, serve as a useful contrast for studying materials with high triplet formation. Additionally, both the exciton quenching effects and the optically pumped heating of the asymmetrically substituted aceacenes hold the potential to be useful in a broad range of applications. The high solubility and solid state order can be exploited in electronic devices where the molecules can be utilized as a protective optical coating for transistors or be diluted into devices to manage exciton densities.^70^ Whereas the strong photothermal response can enable a range of uses from chemical reactions,^71^ photothermal imaging,^72–74^ and targeted therapeutic heating.^75^ This study demonstrates the potential for cyclopentannulation and engineered packing through side chain modification to advance optoelectronic and photothermal applications through control of stability, solubility, and optically pumped heating.

## 11 Methods

### 11.1 Synthesis

Anhydrous tetrahydrofuran, *n*-butyllithium, Tert-Butyldimethylsilylacetylene were purchased from Sigma-Aldrich.^76^ Triethylsilylacetylene, Triisopropylsilylacetylene, Tin­(II) chloride were purchased from Oakwood. All purchased chemicals were used without further purification. Cyclopenta­[*fg*]­naphthacene-1,2-dione^31^ and 1,2-Aceanthrylenedione^77^ were synthesized as reported.

#### 11.1.1 TES-Aceanthracene

Triethylsilylacetylene (1.16 mL, 6.45 mmol) was dissolved in hexanes (70 mL) and cooled to 0 °C using an ice bath. *N*-Butyllithium (2.5 M, 2.1 mL, 5.17 mmol) was added slowly, and the mixture stirred for 1 h at 0 °C. 1,2-Aceanthrylenedione (0.300 g, 1.29 mmol) was added and the reaction mixture stirred overnight at room temperature. The reaction was quenched with saturated NH_4_Cl (aq) (2 mL) and the mixture poured onto a silica plug. Excess silylacetylene was eluted with hexanes, and the desired diol was then eluted with 1:1 CH_2_Cl_2_/acetone. The diol solution was concentrated by rotovap, and the residue redissolved in THF (40 mL) and MeOH (40 mL). Tin­(II) chloride (1.46 g, 6.45 mmol) and 10% HCl (aq) (15 mL) was added. The reaction mixture was stirred at 45 °C overnight. CH_2_Cl_2_ (150 mL) was added and the organic layer was washed with H_2_O (3 × 100 mL). The organic layer was evaporated, and the product purified by chromatography on silica (10:1 Hexanes:CH_2_Cl_2_) to yield a dark brown solid (0.330 g, 53%). Single crystals suitable for X-ray analysis were grown by slow cooling of a concentrated acetone solution.

#### 11.1.2 TBDMS-Aceanthracene

Tert-Butyldimethylsilylacetylene (0.54 g, 3.84 mmol) was dissolved in hexanes (20 mL) and cooled to 0 °C using an ice bath. *N*-Butyllithium (2.5 M, 1.23 mL, 3.08 mmol) was added slowly, and the mixture stirred for 1 h at 0 °C. 1,2-Aceanthrylenedione (0.180 g, 0.77 mmol) was added and the reaction mixture stirred overnight at room temperature. The reaction was quenched with saturated NH_4_Cl (aq) (2 mL) and the mixture poured onto a silica plug. Excess silylacetylene was eluted with hexanes, and the desired diol was then eluted with 1:1 CH_2_Cl_2_/acetone. The diol solution was concentrated by rotovap, and the residue redissolved in THF (20 mL) and MeOH (20 mL). Tin­(II) chloride (0.87 g, 3.84 mmol) and 10% HCl (aq) (10 mL) was added. The reaction mixture was stirred at 45 °C overnight. CH_2_Cl_2_ (100 mL) was added and the organic layer was washed with H_2_O (3 × 100 mL). The organic layer was evaporated, and the product purified by chromatography on silica (10:1 Hexanes:CH_2_Cl_2_) to yield a dark brown solid (0.240 g, 65%). Single crystals suitable for X-ray analysis were grown by slow cooling of a concentrated acetone solution.

#### 11.1.3 TES-Acetetracene

Triethylsilylacetylene (1.59 mL, 8.86 mmol) was dissolved in hexanes (100 mL) and cooled to 0 °C using an ice bath. *n*-Butyllithium (2.5 M, 2.83 mL, 7.08 mmol) was added slowly, and the mixture stirred for 1 h at 0 °C. Cyclopenta­[*fg*]­naphthacene-1,2-dione (0.500 g, 1.77 mmol) was added and the reaction mixture stirred overnight at room temperature. The reaction was quenched with saturated NH_4_Cl (aq) (2 mL) and the mixture poured onto a silica plug. Excess silylacetylene was eluted with hexanes, and the desired diol was then eluted with 1:1 CH_2_Cl_2_/acetone. The diol solution was concentrated by rotovap, and the residue redissolved in THF (50 mL) and MeOH (50 mL). Tin­(II) chloride (2.00 g, 8.86 mmol) and 10% HCl (aq) (20 mL) was added. The reaction mixture was stirred at 45 °C overnight. CH_2_Cl_2_ (200 mL) was added and the organic layer was washed with H_2_O (3 × 100 mL). The organic layer was evaporated, and the product purified by chromatography on silica (10:1 Hexanes:CH_2_Cl_2_) to yield a dark brown solid (0.39 g, 42%). Single crystals suitable for X-ray analysis were grown by slow cooling of a concentrated acetone solution.

#### 11.1.4 TIPS-Acetetracene

Triisopropylsilylacetylene (1.99 mL, 8.86 mmol) was dissolved in hexanes (100 mL) and cooled to 0 °C using an ice bath. *n*-Butyllithium (2.5 M, 2.83 mL, 7.08 mmol) was added slowly, and the mixture stirred for 1 h at 0 °C. Cyclopenta­[*fg*]­naphthacene-1,2-dione (0.500 g, 1.77 mmol) was added and the reaction mixture stirred overnight at room temperature. The reaction was quenched with saturated NH_4_Cl (aq) (2 mL) and the mixture poured onto a silica plug. Excess silylacetylene was eluted with hexanes, and the desired diol was then eluted with 1:1 CH_2_Cl_2_/acetone. The diol solution was concentrated by rotovap, and the residue redissolved in THF (50 mL) and MeOH (50 mL). Tin­(II) chloride (2.00 g, 8.86 mmol) and 10% HCl (aq) (20 mL) was added. The reaction mixture was stirred at 45 °C overnight. CH_2_Cl_2_ (200 mL) was added and the organic layer was washed with H_2_O (3 × 100 mL). The organic layer was evaporated, and the product purified by chromatography on silica (10:1 Hexanes:CH_2_Cl_2_) to yield a dark brown solid (0.42g, 39%). Single crystals suitable for X-ray analysis were grown by slow cooling of a concentrated acetone solution.

### 11.2 Characterization

Proton and carbon NMR spectra were collected using a 400 MHz spectrometer. Chemical shifts of each spectrum are reported in ppm and referenced to deuterated chloroform solvent (see SI). MALDI. UV–visible spectra were measured using a 10 mm cuvette and solutions approximately 10^–3^ mol/m^3^. Cyclic voltammetry was measured at a scan rate of 50 mV/s with a button glassy carbon working electrode, a platinum wire counter electrode and an Ag/AgCl reference electrode. A solution of 0.1 M Bu_4_NPF_6_ in dichloromethane was used as a supporting electrolyte solution under a blanket of N_2_ with Fc/Fc+ as an internal reference.

X-ray diffraction data were collected at either 90.0(2) K (k13054, x13133) or 100.0(2) K. Raw data were integrated, scaled, merged and corrected for Lorentz-polarization effects using either DenzoSMN^78^ (k13054) or APEX2/3 ^79^ (x13133, m23163). The structures were solved by dual-space methods (SHELXT)^80^ and refined against F^^2^ by weighted full-matrix least-squares (SHELXL).^81^ Hydrogen atoms were found in difference maps but subsequently placed at calculated positions and refined using riding models. Non-hydrogen atoms were refined with anisotropic displacement parameters. The final structure model was checked using established methods.^82,83^ Atomic scattering factors were taken from the International Tables for Crystallography.^84^

### 11.3 Optical Measurements

Transient absorption measurements were performed utilizing the 1030 nm, 200 fs output of a 1 MHz Yb laser. The experiment was performed at a repetition rate of 3 kHz. A 50/50 beam splitter sends half of the fundamental to an optical parametric amplifier which enabled pump wavelengths of 415 nm, 575 and 830 nm to be used. The remaining fundamental beam is focused into a cuvette filled with water to generate the supercontinuum probe spanning 390–950 nm. Continuous pump delays up to about 30 ns were obtained by multipassing two retroreflectors along an approximate 3 m delay line. For delays beyond 30 ns, a 405 nm laser diode of variable pulse duration (minimum 7 ns) was operated via external trigger at 3 kHz and used as the pump.

For solid state measurements, thin film samples were grown from 10 mg/mL solutions in toluene drop cast on to cleaned fused quartz or sapphire substrates. Slow drying using a glass cover and nearby small beaker of toluene over a minimum of 1 h achieved thin flat crystals larger than 100 μm on a side for **TBDMS-aceAN**, **TES-aceTN**, and **TIPS-aceTN**. **TES-aceAN** formed a polycrystalline film with preferential orientation of the crystallites, but no crystals larger than the light spot were formed. Absorption measurements were performed using the white light continuum source focused onto the samples using a microscope objective. A second microscope objective behind the sample collimated the transmitted light. Transmitted and reflected light spectra were measured using a spectrometer and CCD camera.

Heated absorption measurements were performed under a continuous flow of N_2_ (g) in an optical cryostat. Silver paint was used to create thermal contact between the glass substrate and coldfinger of the cryostat. A temperature controller was used to heat the samples. Each temperature was allowed to equilibrate for ≈30 min before measuring the absorption spectrum. Due to the thickness of the cryostat, the microscope objectives were replaced with short focal length achromats, and a xenon lamp was used as the light source. Polarizers were placed before and after the crystals to ensure that optical axes were probed. The transmitted spectra were recorded using the spectrometer and CCD camera.

All solution state transient absorption measurements were performed with magic angle polarization between the pump and probe beams. Solid state measurements employed λ/2 waveplates in the pump and probe paths to excite and probe the crystals along only the optical axes. A polarizer placed after the sample ensured that only probe light polarized along the optical axis was collected.

Because the supercontinuum pulses are significantly chirped after propagation through the water in the cuvette, each transient absorption spectrum needed to be corrected to extract the early time dynamics. This was accomplished by fitting the early time response with a polynomial and using that fit to correct time zero for each frequency of light in the probe beam. Our correction technique was verified by using a spatial light modulator to precompensate the chirp over a narrow wavelength range showing that the subpicosecond dynamics were indeed the intrinsic molecular response.

### 11.4 Computational Details

All calculations were run using Gaussian 16 ^85^ at CAM-B3LYP-D3­(BJ)/6–311++G**/PCM­(toluene) level of theory. CAM-B3LYP is a long-range corrected functional that is known to capture charge transfer TD-DFT excitations better than standard hybrid functionals. D3­(BJ) dispersion provides necessary density corrections for the highly delocalized densities of the dimers. This combination of methods was chosen to provide the best orbital and density descriptions so that the most chemical understanding can be gained from the quantum mechanical calculations. The predicted energies of transitions are slightly high as expected from standard CAM-B3LYP.

The CATNIP code was used to calculate the electronic coupling [https://github.com/JoshuaSBrown/QC_Tools]. All optimized geometries can be downloaded at https://github.com/fredingroup/organics/tree/main/blueTEN.

## Supplementary Material



## Data Availability

The data that support the findings of this study are available from the corresponding author upon reasonable request.

## Notes

Chemical shifts, mass spectrometry, calculation of experimental oscillator strengths, discussion of PL and PLQY, photostability, extended solution absorption data, steady-state crystalline spectroscopy, time-resolved analysis, thermal effect analysis, redox properties, characterization spectra, and theoretical calculations and TDDFT tables (PDF)

## ⊥

Division of Chemistry, Alfred University, Alfred, NY 14802, United States
