# Supplementary material for: Enhanced Photostability through Rapid Exciton Decay in Desymmetrized Cyclopentannulated Acenes with Strong Face-to-Face pi Stacking
Source: Chem Mater. 2026 Jan 28;38(3):1392–406. doi: 10.1021/acs.chemmater.5c02815 (PMC12895388; doi:10.1021/acs.chemmater.5c02815)
Supplement: Supplementary file 1 [file cm5c02815_si_001.pdf]

Supporting Information for

Enhanced photostability through rapid exciton decay in desymmetrized cyclopentannulated acenes with strong face-to-face pi stacking

Chad D. Cruz,[1,*] Karl J. Thorley,[2] Zachary Knepp,[3,†] Jared Wahlstrand,[1] Gil M. Repa,[3] John C. Stephenson,[1] Sean Parkin,[4] Lisa A. Fredin,[3] John E. Anthony[2,4] and Emily G. Bittle[1,*]

1 National Institute of Standards and Technology, Physical Measurement Laboratory, Gaithersburg, MD, 20899, USA

2 Center for Applied Energy Research, University of Kentucky, Lexington, KY, 40511, USA

3 Lehigh University, Department of Chemistry, Bethlehem, PA, 18015, USA

4 Department of Chemistry, University of Kentucky, Lexington, KY, 40506, USA

† Present address: Division of Chemistry, Alfred University, Alfred, NY, 14802, USA

* Email: chad.cruz@nist.gov

* Email: emily.bittle@nist.gov

Contents

Chemical Shifts and Mass Spectrometry S2

Calculation of Experimental Oscillator Strengths S3

Discussion of Photoluminescence and PLQY S3

Photostability of the Cyclopentannulated Acenes S6

Extended Solution Absorption Data S7

Steady-State Crystalline Spectroscopy S9

Time-resolved Analysis: Solutions S10

Time-resolved Analysis: Crystals S12

How Thermal Effects May Influence TA Measurements S13

Redox Properties S19

Theoretical Calculations S20

Copies of Characterization Spectra S27

TDDFT Tables S38

References S66

Chemical Shifts and Mass Spectrometry

Proton and carbon NMR spectra were collected using a 400 MHz spectrometer. Chemical shifts of each spectrum are reported in ppm and referenced to deuterated chloroform solvent.

TES-aceanthracene

^{1}H NMR ($CDCl_3$, 400 MHz) δ 9.27 (1H, d, J = 8.7Hz), 8.49 (1H, s), 8.06 (1H, d, J = 8.4 Hz), 8.03 (1H, d, J = 8.7 Hz), 7.91 (1H, d, J = 6.7 Hz), 7.57-7.65 (2H, m), 7.48 (1H, dd, J = 6.9, 7.2 Hz), 1.12-1.20 (18H, m), 0.74-0.86 (12H, m)

^{13}C NMR ($CDCl_3$, 100 MHz) δ 138.7, 134.6, 131.7, 130.4, 130.2, 129.5, 128.3, 127.6, 127.3, 126.2, 126.1, 125.6, 125.2, 124.8, 123.9, 105.3, 103.4, 103.3, 100.7, 7.7, 7.7, 4.6, 4.6

MS (MALDI,+ve) Calculated for $C_{32}H_{38}Si_2$: 478.2512, Found: 478.190

TBDMS-aceanthracene

^{1}H NMR ($CDCl_3$, 400 MHz) δ 9.24 (1H, d, J = Hz), 8.49 (1H, s), 8.02-8.08 (2H, m), 7.92 (1H, d, J = Hz), 7.57-7.65 (2H, m), 7.46 (1H, m), 1.12 (9H, s), 1.09 (9H, s), 0.33 (6H, s), 0.28 (6H, s)

^{13}C NMR ($CDCl_3$, 100 MHz) δ 138.6, 134.6, 131.7, 130.4, 130.4, 130.2, 129.5, 128.4, 127.6, 127.3, 126.2, 126.1, 125.6, 125.2, 124.8, 123.9, 106.0, 104.1, 102.9, 100.2, 26.3, 16.9, 16.8, -4.4, -4.5

MS (MALDI,+ve) Calculated for $C_{32}H_{38}Si_2$: 478.2512, Found: 478.121

TES-acetetracene

^{1}H NMR ($CDCl_3$, 400 MHz) δ 9.30 (2H, m), 8.64 (2H, m), 8.02 (2H, m), 7.55 (2H, m), 7.41 (2H, m), 1.21 (18H, t, J = 7.8 Hz), 0.85 (12H, q, J = 7.8 Hz)

^{13}C NMR ($CDCl_3$, 100 MHz) δ 134.3, 131.7, 131.6, 129.2, 125.4, 125.0, 124.1, 104.5, 104.1, 7.7, 4.7

MS (MALDI,+ve) Calculated for $C_{36}H_{40}Si_2$: 528.2669, Found: 528.197

TIPS-acetetracene

^{1}H NMR ($CDCl_3$, 400 MHz) δ 9.42 (2H, d, J = Hz), 8.65 (2H, s), 8.03 (2h, d, J = Hz), 7.52 (2H, m), 7.40 (2H, m), 1.27-1.32 (42H, m)

^{13}C NMR ($CDCl_3$, 100 MHz) δ 134.2, 131.8, 131.7, 131.6, 130.6, 129.0, 125.2, 125.0, 124.2, 124.2, 122.1, 104.8, 103.5, 18.9, 11.6

MS (MALDI,+ve) Calculated for $C_{42}H_{52}Si_2$: 612.3607, Found: 612.295

Calculation of Experimental Oscillator Strengths

To calculate the experimental oscillator strengths (f) shown in Table S1, we use the following equation derived by Tarleton et al. for comparison to DFT[1]:

$$f_{\text{exp}} = (4.319 \times 10^{-9})\, n \int \varepsilon(\tilde{\nu})\, d\tilde{\nu} \qquad \text{(Eqn. 1)}$$

where n is the refractive index of the solvent; $\tilde{\nu}$ is the frequency of light absorbed given in wavenumbers; and $\varepsilon(\tilde{\nu})$, the extinction coefficient expressed in units of (M^{-1} cm^{-1}), is integrated over a range of wavenumbers for the band of an electronically excited state. The integration range for each of our observed peaks and corresponding f_{exp} values are given in Table S1. The refractive index for each solvent used is n = 1.39 (isopropanol), n =1.45 (chloroform), n = 1.5 (toluene).

Discussion of Photoluminescence and PLQY

Measurements of the photoluminescence (PL) quantum yield (QY, photons emitted per photon absorbed) were made for TES-aceAN, TBDMS-aceAN, TES-aceTN and TIPS-aceTN in dilute solution. Two spectrofluorometers were used for detecting PL. One for emission in the wavelength range 300 nm to 900nm and a second for emission wavelengths in the range 900 nm to 1800nm.

We did not attempt an ab initio measurement of photons emitted to photons absorbed. Rather, we compared PL from our samples to a PL standard (i.e., dyes such as Coumarin 6 - C6- with well-established absorbance spectrum, emission spectrum and QY). This is common practice, but several details must be considered. Dilute solutions were made in spectrograde solvents transferred to 1 cm quartz cuvettes for the measurements. Concentrations were chosen so that there would be no significant depletion of emission through self-absorption and the excitation light was also not significantly depleted by sample absorption. Solutions were matched so the reference (e.g., C6) and the sample (e.g., TES-aceTN) had the same total absorbance of the exciting light in the cuvette. Since that is true, then the QY of the TES-aceTN is derived by comparing its PL to the C6 PL and its QY. That is,

$$PLQY_{(TES-aceTN)} = \frac{PL_{(\text{TES-aceTN})}}{PL_{C6} \times PLQY_{C6}} \qquad \text{(Eqn. 2)}$$

Results were checked for consistency using cuvettes of varying lengths and solutions of varying concentrations, various solvents, excitation at various wavelengths and various intensities (to check for sample decomposition). Figure S1 shows typical PL spectra for the reference dye (coumarin C6) and an aceacene (TBDMS-aceAN in isopropanol). Note that the aceacene signal is multiplied by 40,000×. We compared the integral of the TBDMS signal (ignoring the scattered excitation light at 481 nm) to the integral of the C6 PL. That ratio gave the value shown in Table S2 (PLQY < 4e-5). There are many possibilities for the origin of the extremely weak signal from the aceacene sample. It could be due to PL from impurities present at extremely low levels or some stray light inside the monochromator. Conceivably the feature at 561 nm could be Raman scattering from CH vibrational modes. For the TBDMS sample, a constant photomultiplier tube dark count value of ≈900 cps was subtracted from the data, a value to be compared to 5640 cps at maximum of the 562 nm feature. The broad feature from 600 nm to 800 nm is only 216 cps compared to the 900 cps background. Obviously the integral of 900 cps background over the spectral range red of the 481 nm excitation significantly exceeds the integral of the displayed “signal”. An incorrect background could introduce significant uncertainty in the derived PLQY and the subsequent calculations

using it (see below). Given the small signal compared to the dark counts, there may be significant uncertainty despite the extreme care taken when performing the measurement. The conclusion that there is negligible PL from any of the aceacene samples is certainly correct, but the interpretation of any non-zero signal is not possible at these low levels. The results are summarized in Table S2. For comparison to the extremely small QY for the aceacenes under study in this paper, the reference polyaromatic TMS-TN = bis(trimethylsilylethynyl) tetracene is also shown, for which we measured PLQY = 0.6.

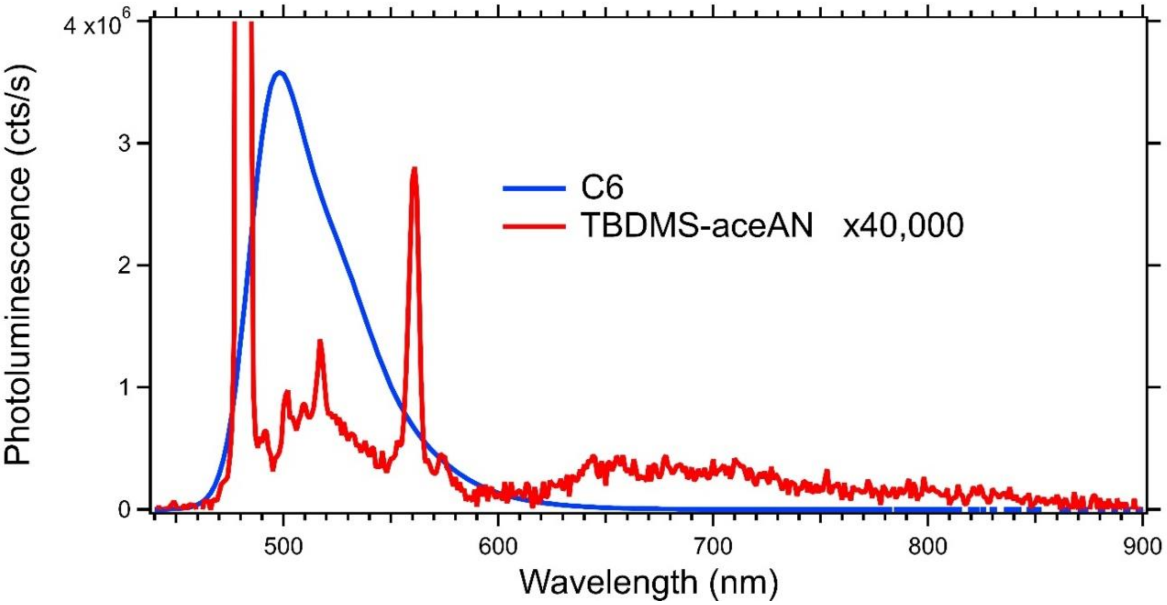

Figure S1. Photoluminescence (PL) from TBDMS-aceAN compared to a standard dye molecule C6 (for which the PLQY is 1), both dilute (0.069 mol/m^3 and 0.015 mol/m^3, respectively) in isopropanol. The TBDMS signal has been multiplied by 40,000 to bring it on scale with the C6 signal. Absorption of excitation light from the spectrofluorometer was the same for each sample.

It is known that energetic transitions with strong absorption typically have strong radiative transitions. The very low photoluminescence quantum yield (PLQY) for these molecules in solution is interesting because it implies extremely fast non-radiative rates, $k_{NR}= 1/\tau_{NR}$, compared to the radiative rates $k_{rad}=1/\tau_{rad}$, as PLQY= $1/\tau_{rad}/(1/\tau_{rad} + 1/\tau_{NR})$, or for small PLQY the $1/\tau_{NR} = 1/ (\tau_{rad} \times \text{PLQY})$. Given that PLQY $\leq 2 \times 10^{-4}$ for the four molecules, the $1/\tau_{NR} > 5000/\tau_{rad}$. To rule out any transitions at lower energies, we measured the fluorescence to 1800 nm and present quantum yields in Table S2. For comparison to the extremely small PLQY for molecules **TES-aceAN**, **TBDMS-aceAN**, **TES-aceTN**, **TIPS-aceTN**, the reference polyaromatic TMS-TN is also shown.

To emphasize this point, we estimate the expected molecular band fluorescence rates based on the strong absorption of the molecules using:[2]

$$\frac{1}{\tau_0} = 2.880 \times 10^{-9} n^2 \tilde{\nu}^2 \int \varepsilon(\tilde{\nu})\, d\tilde{\nu} \qquad \text{(Eqn. 3)}$$

where $\tilde{\nu}$ is the transition frequency given in units of cm^{-1}, n is the refractive index of the solvent, ε is the molar extinction coefficient, and τ_0 is the reciprocal of the rate constant, which is the maximum possible mean life of the upper state if the only decay is by spontaneous emission to a lower state. This equation is rigorous for atomic transitions where the band is sharp and the absorption and PL occur at the same wavelength; however, it is also useful for an approximate calculation of the radiative rate in molecules with

broad, Stokes-shifted PL spectra. We can obtain a simplified expression for our estimation by substituting Eqn 1 into Eqn. 3 which yields the equation given in the main text,

$$\frac{1}{\tau_{rad}} \approx \frac{2}{3} n^2 \nu^2 f \quad \text{(Eqn. 4)}$$

where f is the experimentally determined oscillator strength. First we apply equation 3 to coumarin 6 which has a QY of 1 in iPA and obtain $\tau_{rad} \approx 2.57$ ns which agrees nicely with the known PL lifetime[3] ($\tau = 2.4$ ns). The values for the aceacenes and the reference polyaromatic TMS-TN are shown in table S1. Despite the success of these calculations with coumarin 6 and a decorated tetracene molecule (TMS-TN), the aceacene nonradiative lifetimes are underestimated. For instance, the data from tables S1 and S2 imply the decay time for TES-aceAN in isopropanol should be 0.66 ps, not the 15 ps measured in figure 3, a discrepancy of 23×. The above equations are generally reliable for well-behaved molecules, like laser dyes and unsubstituted aromatics, since they are derived under the assumption of equally strong absorptive and emissive, optically allowed transitions with small Stokes' shifts. The lowest energy forbidden band present in the aceacenes suggests that these equations will already not accurately describe the photophysics. Furthermore, the lack of any detectable PL makes characterizing the magnitude of the Stokes' shift impossible. However, excited state conformational changes are likely in the aceacenes since the pi system extends beyond the relatively rigid anthracene and tetracene cores into the five-membered ring and the flexible solubilizing substituents.

Table S1. Calculated expected fluorescence rates and estimated radiative rates based on absorption. This table includes UV absorption peaks not included in Table 1 of the main text.

Molecule	Solvent	Peak Frequency (cm^{-1})	Wavelength Range (nm)	f_{exp}	$1/\tau_0$ (ns^{-1})	τ_{rad} (ns)
Coumarin 6	Isopropanol	18,519	346 – 540	0.88	0.39	2.57
TMS-TN	Toluene	18,727	422 – 562	0.15	0.074	13.5
**TES-aceAN**	Chloroform	15,625	399 – 750	0.19	0.065	15.4
	Isopropanol	15,625	394 – 899	0.24	0.075	13.2
**TBDMS-aceAN**	Chloroform	15,625	399 – 750	0.20	0.068	14.6
	Isopropanol	15,625	394 – 899	0.18	0.057	16.2
		26,110	340 – 394	0.10	0.088	11.4
		37,170	250 – 340	0.98	1.74	0.57
**TES-aceTN**	Toluene	17,422	452 – 899	0.16	0.036	27.7
**TIPS-aceTN**	Toluene	17,422	452 – 899	0.16	0.036	27.7
	Isopropanol	17,422	452 – 899	0.15	0.029	34.4
		25,150	342 – 435	0.17	0.14	7.22
		35,590	257 – 342	1.54	2.51	0.40

Table S2. PLQY measured in solution for **TES-aceAN**, **TBDMS-aceAN**, **TES-aceTN**, and **TIPS-aceTN**. and the reference molecule **TMS-TN**. Due the uncertainties in the measurements described above, the very low PLQY values for the aceacenes are given as lower limits (e.g., the few photons observed could be due to PL from a tiny impurity, from Raman scattering, from monochromator issues; any actual PL could be less than, but not more than total photons observed). Numbers are given to 1 significant figure for the aceacenes.

Molecule	Solvent	Excitation Wavelength (nm)	Quantum Yield ($\times 10^{-5}$)
**TMS-TN**	Isopropanol	534	60,000
**TES-aceAN**	Isopropanol	495	<3
		426	< 4
		384.5	< 5
		314	< 7
	Chloroform	405	< 2
**TBDMS-aceAN**	Isopropanol	481	< 4
		390	< 5
		277	< 20
	Chloroform	405	< 2
**TES-aceTN**	Toluene	580	< 2
**TIPS-aceTN**	Isopropanol	571	< 6
		376	< 40
		327	< 50

Photostability of the Cyclopentannulated Acenes

We quantitatively determined the photodissociation quantum yield (PDQY), defined as molecules dissociated/photons absorbed, for TBDMS-aceAN, TES-aceAN, and TIPS-aceTN dilute (<1mM) in isopropyl alcohol. Samples were not de-oxygenated. The apparatus used was the Fluorolog 3 mentioned in the PLQY experiments. The excitation power incident on the samples was measured by calibrated power meters and was in range 4-20 mW in a 4 mm diameter spot. Absorption spectra were recorded just before and after irradiation, which was 4 h to 20 h in duration. Standard quartz cuvettes of 1, 2, 4, and 10 mm pathlength were used. Since the spectrofluorometer may emit UV radiation in second order (i.e., excitation at nominal 500 nm might include some slight unwanted light at 250 nm), UV blocking filters were used to prevent that. We assumed that photodissociation would manifest as a change in absorbance according to Beers law, so

$$\frac{OD_{Post\ Irradiation}}{OD_{Initial}} = \frac{Molecular\ Density_{Post\ Irradiation}}{Molecular\ Density_{Initial}}$$

from which the decrease in number of aceacene molecule per photons absorbed (PDQY) was calculated. The results are shown in Table S3.

Table S3. Photodissociation Quantum Yields (PDQY) for molecules in dilute isopropyl alcohol solutions. The excitation wavelengths (nm) are shown along with PDQY values.

Molecule (in iPA)	Excitation Wavelength (nm)	Photodissociation Quantum Yield
**TBDMS-aceAN**	467	$< 7.7\times10^{-6}$
**TES-aceAN**	547	$< 3.0\times10^{-7}$
**TES-aceAN**	467	$< 1.8\times10^{-6}$
**TES-aceAN**	368	$< 6.8\times10^{-6}$
**TES-aceAN**	269	$> 7.1\times10^{-4}$
**TIPS-aceTN**	572	$< 6.2\times10^{-6}$
**TIPS-aceTN**	393	$< 8.5\times10^{-6}$
TMS-TN	527	1.9×10^{-4}

For comparison to the three aceacenes, TMS-TN was studied. At 527 nm, coinciding with a vibronic peak in its lowest absorption, it gave PDQY = 1.5×10^{-4}. This compares to TES-aceAN excited to its lowest absorption at 547 nm, where PDQY is $< 3.0\times10^{-7}$, which is at least 500 times more stable than TMS-Tet. We also estimated PDQY for molecules in solid state crystal form, in the absence of oxygen, using a similar approach. From the known fluence of our pump laser (cm^{-2}) per pulse, the absorption coefficient $\alpha(cm^{-1})$ at the pump wavelength, the number density of molecules in the crystal, and the number of pulses the sample could absorb without observable change in its absorption spectrum, an upper bound on the PDQY was estimated. Specifically, we present data for 415 nm pump (see Figure S7), used to study the long-time TA. For excitation at 3.5 mJ/cm^2 (7.3×10^{15} cm^{-2}), crystal of 0.8 μm thickness, and $\alpha_{(415)} = 3.4\times10^4$ cm^{-1} (short axis polarization; 93% absorption), 6.8×10^{15} cm^{-2} are absorbed on each excitation pulse. The density of TES-aceTN is 1.18×10^{21} cm^{-3}. At a repetition rate of 1500 Hz we observe < 5% change in sample absorption in 30 min (2.7×10^6 pulses). That is, after (6.8×10^{15} cm^{-2}) · (2.7×10^6 pulses) = 1.8×10^{22} cm^{-2} photons have been absorbed by molecules (1.18×10^{21} cm^{-3}) · (0.8 μm) = 9.4×10^{16} cm^{-2}, less than 5% of them dissociated; giving an upper bound of [(9.4×10^{16} cm^{-2}) · (0.05)] / (1.8×10^{22} cm^{-2})= $PDQY_{(415\ nm)} < 2.6\times10^{-7}$ for TES-aceTN as a crystal. Note that these calculations are for conditions of very high laser irradiation, where nonlinear effects might contribute. Under less intense illumination, one might expect a smaller PDQY.

Extended Solution Absorption Data

In figure S2 we show additional absorption data including peaks in the 183 nm to 270 nm region. Minimal to no shifts are seen in absorption peaks between solvents with varying polarity, toluene (141.8 kJ/mol), chloroform (163.6 kJ/mol), and isopropyl alcohol (202.5 kJ/mol).[4] The solution concentrations used in the absorption and quantum yield studies in this paper are small, in the 0.01 mol/m^3 to 1 mol/m^3 range. The

absorption spectra and PLQY measurements were independent of concentration in that range. That suggests but does not prove we studied only isolated molecules. However, previous studies on similar molecules in solution at room temperature strongly support the idea that there is no aggregation at such low concentrations. Room temperature solution studies on molecules such as TIPS-tetracene and highly substituted anthradithiophene (ADT) derivatives at concentrations in the 140 mol/m^3 to 260 mol/m^3 range (140 to 260 times our maximum concentrations) saw no evidence for aggregation.[5-8]

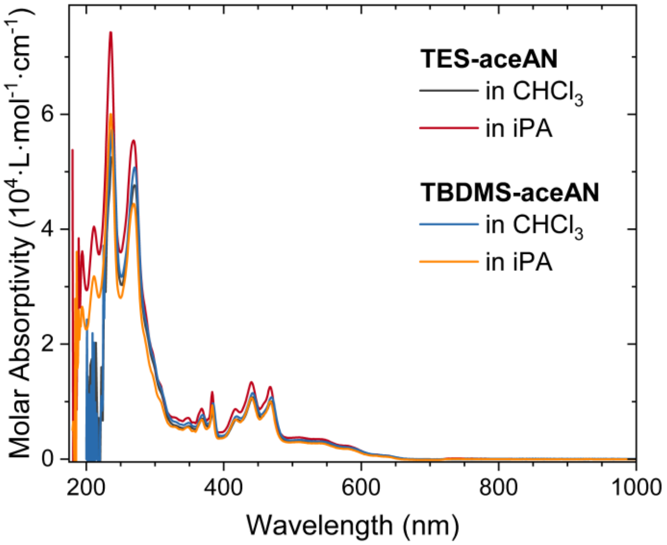

Figure S2a. Molar Absorptivity (M^{-1}cm^{-1}) for **TES-aceAN** and **TBDMS-aceAN** in chloroform ($CHCl_3$) and isopropanol (iPA). Spectra for **TES-aceAN** and **TBDMS-aceAN** overlap very closely in spectral shape although the **TES-aceAN** absorbance seems marginally greater than **TBDMS-aceAN** in both solvents. The solvatochromic shift between the two solvents is very slight, with the absorbance in iPA being 2 nm blue of that in chloroform.

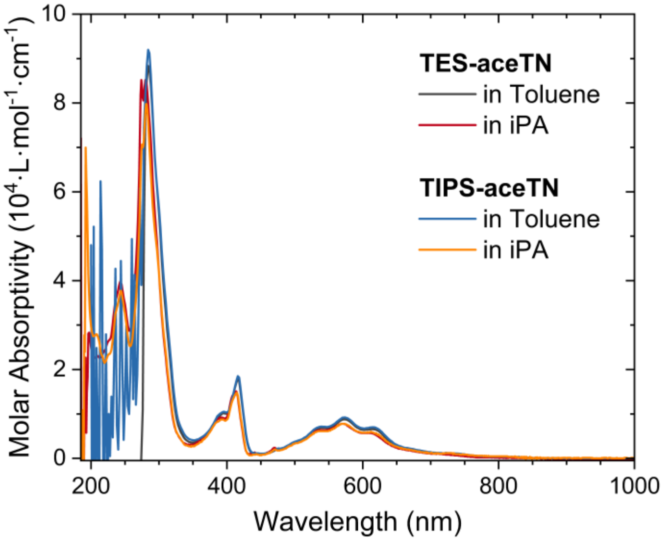

Figure S2b. Molar Absorbance (M^{-1}cm^{-1}) for **TES-aceTN** and **TIPS-aceTN** in toluene and isopropanol (iPA). In toluene, **TES-aceTN** and TIPS-aceTN overlap very closely in spectral shape and intensity. In isopropanol, the absorbance of **TIPS-aceTN** was measured absolutely, whereas the absorbance of **TES-aceTN** was measured for a sample of unknown concentration. The **TES-aceTN** spectrum was scaled by a factor of 2 to match the **TIPS-aceTN** spectrum. The spectral shapes for both molecules are identical in iPA and in toluene. The solvatochromic shift between the two solvents is very slight, with the spectra in iPA being 2 nm blue of the spectra in toluene.

Steady-State Crystalline Spectroscopy

All crystalline measurements were performed in a home-built purge cell with constant N_2 (g) flow. Transmission and reflection from the crystals were collected and used to calculate absorption spectra using A = 1 – T – R, with the exception of **TES-aceAN**, where increased scattering prevented the reflectance from being collected. Polarized absorption was measured along the optical axes as determined with cross polarizers. Optical microscope images of the four samples are shown in Figure S3.

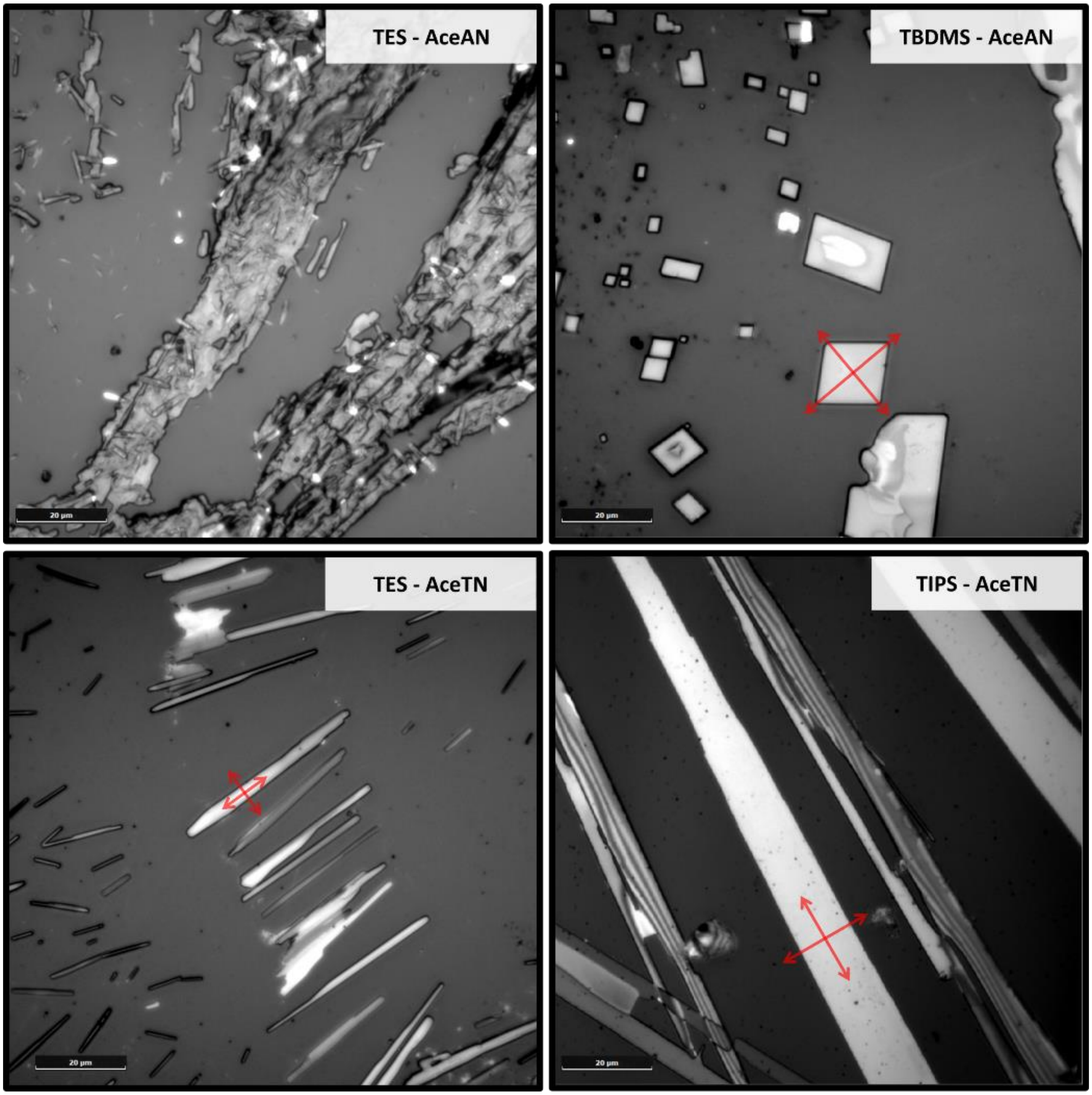

Figure S3. Images representative of the crystals grown and measured in this work: (top, left) polycrystalline sample of TES-aceAN, (top, right) square single crystals of TBDMS-aceAN, (bottom, left) small needle-like crystals of TES-aceTN, (bottom, right) large needle-like crystals of TIPS-aceTN. Red arrows show the optical axes relative to the crystal shape. Scale bar is 20 µm.

Time-resolved Analysis: Solutions

To find spectral evidence of triplet formation in our TA experiments, one needs the spectral signature of an aceacene triplet. Separate from our experiments where we directly excite the aceacene molecules, we performed triplet sensitization experiments in dilute solutions using N-methylfulleropyrrolidine (NMP) as a source of triplets. As discussed quantitatively below, the only case where we saw triplet excitons in aceacenes was in sensitizer experiments, never after directly exciting the aceacenes. After excitation of the NMP sensitizer singlet state using a 405 nm laser diode, NMP rapidly undergoes ISC to form triplets[9] which can then be transferred via diffusional collisions to the aceacenes. The TA spectra of degassed NMP is shown in Figure S5a. The 16 ns pulse from the laser diode is too long to capture the conversion of singlet induced absorption (SIA) into triplet induced absorption (TIA). At these timescales only the TIA of the NMP triplet and the subsequent decay are observed. In a separate experiment on mixed NMP and TIPS-aceTN, we initially see the NMP TIA around 700 nm which decays more rapidly as a new positive feature emerges from 440 nm to 530 nm which we attribute to TIA from TIPS-aceTN. Performing a target analysis assuming a sequential mechanism of triplet formation on NMP which then transfers the triplets to TIPS-aceTN before the TIPS-aceTN triplets decay, we find a triplet transfer time of ≈300 ns and a triplet lifetime of ≈1 μs (Figure S6). The observed triplet transfer time of 0.3 μs is close to the diffusion limited collision time between NMP and TIPS-aceTN of 0.1 μs in toluene. Despite the TIA being readily visible over our probe wavelengths, we see no evidence of triplet formation without the presence of the NMP sensitizer. A quantitative limit on the fraction of singlet aceacenes that form triplets is obtained as follows. The number density of excited NMP molecules, [NMP*], is calculated from the absorption coefficient of NMP at 405 nm and fluence of the 405 nm diode laser. That is the maximum density of aceacene that could give rise to the 0.65 mOD absorption at 520 nm seen in Figure S5b. In the aceacene TA experiments we saw no absorption at 520 nm (e.g., Figure 3, main text), specifically less than 5% of the 0.65 mOD seen in Figure S5b; therefore, the total triplet density in the TA experiments was $< 0.05 \cdot$ [NMP*]. The total number of excited singlet aceacene molecules in the TA experiments (e.g., [TIPS-aceTN*]), was similarly calculated from the absorption coefficient of aceacenes at 415 nm (see Figure 2, main text) and fluence of the 415 nm pump laser. The ratio ($0.05 \cdot$ [NMP*]) / [TIPS-aceTN*] gives a limit on the number of triplets formed per excited singlet TIPS-aceTN molecule as $< 1.4\times10^{-3}$. Similar results hold for the other aceacenes. The triplet yields in solution are insignificant.

A model-based, target analysis was adapted from reference 10 for spectral deconvolution of the solution TA measurements. The raw TA spectra were first corrected to account for the temporal chirp present in the probe beam after white light generation in a water cell. This was accomplished by fitting a polynomial to the wavelength-dependent instantaneous response of the solution and subtracting this polynomial from the data such that all the wavelengths of the white light probe arrived at time zero. The error from the least-squares fitting yields uncertainties that are better than the time resolution of the system. We previously determined[11] the temporal resolution of this pump-probe system to be 200 fs and we estimate the uncertainty in the lifetimes derived from the target analysis to be $\pm$ 500 fs. The largest uncertainty in the amplitude coefficients arises in the determining the laser fluence which is a key parameter in our estimation of possible triplet concentrations generated by the laser pulse. By testing 3 nominally identical energy meters against each other we arrive at an uncertainty in average laser power of approximately 10%. By comparing knife edge measurements of the beam waist with images of the beam at the sample, we estimate the uncertainty in our spot size to be approximately 15%. Upon adding these major sources of uncertainty in quadrature and rounding up, we expect that the fluences reported here have error bars of about 20%. Importantly, our conclusions do not rely upon an absolute determination of these parameters as we are more interested in setting limits for possible triplet generation.

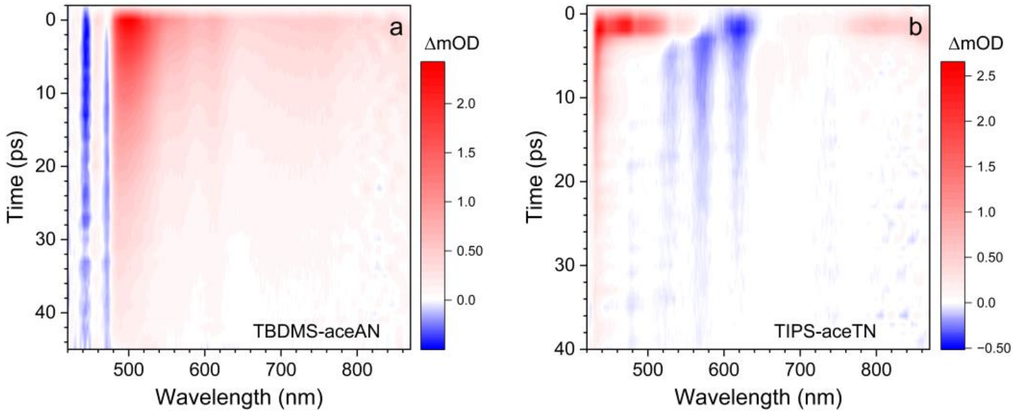

Figure S4. Transient absorption spectra of 1 mM, degassed solutions of (a) TBDMS-aceAN in chloroform and (b) TIPS-aceTN in toluene. Both spectra were excited with a 415 nm pump beam and were probed with white light continuum utilizing magic angle polarization between pump and probe.

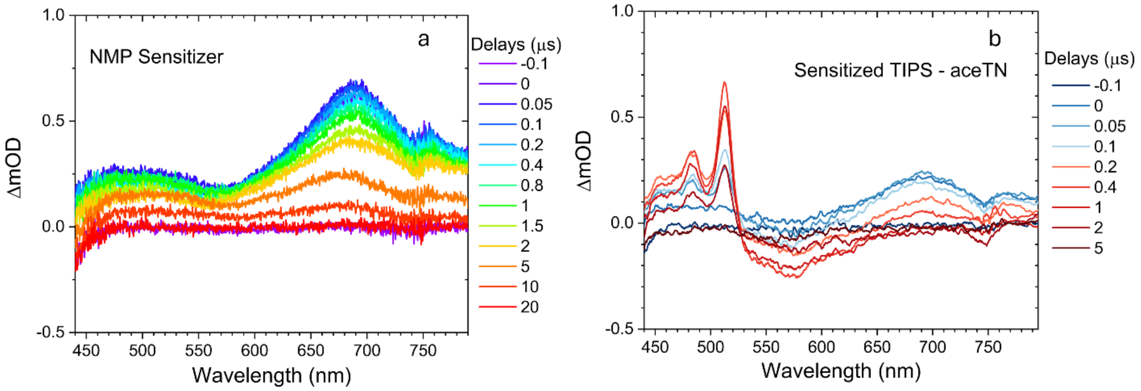

Figure S5. Transient absorption spectra of degassed, (a) 1 mol/m^3 N-methylfulleropyrrolidine (NMP) in chloroform and (b) a solution of 1.7 mol/m^3 NMP with 0.8 mol/m^3 TIPS-aceTN in chloroform.

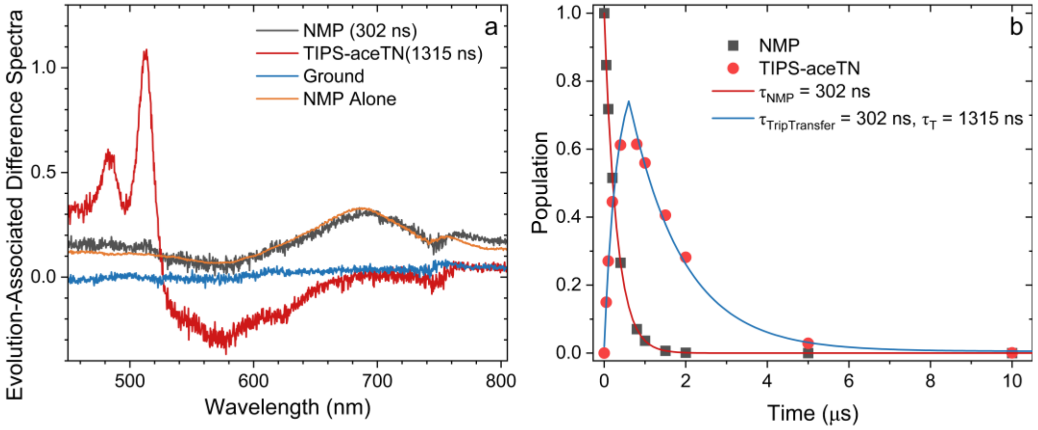

Figure S6. (a) Evolution-associated difference spectra for a solution of TIPS-aceTN with NMP sensitizer. Spectral deconvolution identifies a feature wholly attributable to the NMP sensitizer (dark gray) which is confirmed by overlaying the difference spectrum of NMP alone in solution (orange). This feature decays with a lifetime of 302 ns with the concomitant growth of the TIPS-aceTN feature (red) indicating triplet transfer to the TIPS-aceTN. (b) The population dynamics extracted from the global analysis show the growth of the TIPS-aceTN triplet induced absorption which then decays with a lifetime of 1.3 μs.

Time-resolved Analysis: Crystals

Fluence dependent measurements were performed on the crystals to ensure that nonlinear processes such as exciton-exciton annihilation were not contributing to the observed dynamics. The fluences used throughout these experiments are much higher than one typically would use (< 100 μJ/cm^2) during pump probe experiments; however, we found that the dynamics were independent of fluence over the range used in this study (Figure S7). We opted for higher fluences to increase the signal to noise which was necessary to obtain signal for certain polarization combinations (e.g. Fig S8).

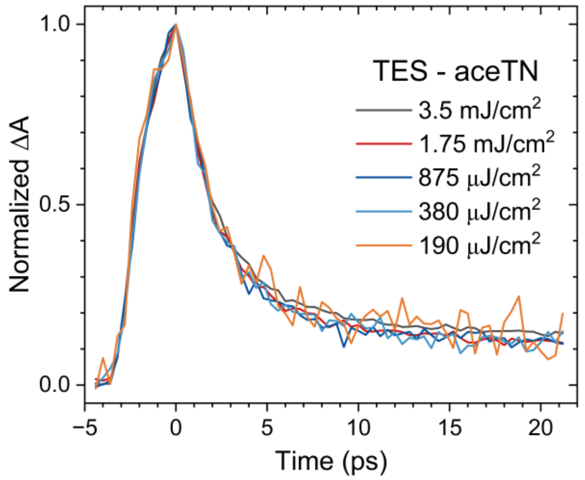

Fig S7. Normalized kinetic trace of the singlet induced absorption for TES-aceTN crystals as a function of 415 nm pump laser fluence.

Pump wavelengths of 575 nm and 830 nm were used to ensure that the negative TA features in the red part of the spectra were indeed a ground state bleach (GSB) rather than stimulated emission. In figures S8 and S9 we see that very similar TA spectra are obtained regardless of pump wavelength. Furthermore, this shows that the rapid heating response is not simply due to internal conversion (IC) from the higher lying excited states accessed with the 415 nm pump beam. The long-lived artifacts are present for each pump wavelength employed in this study.

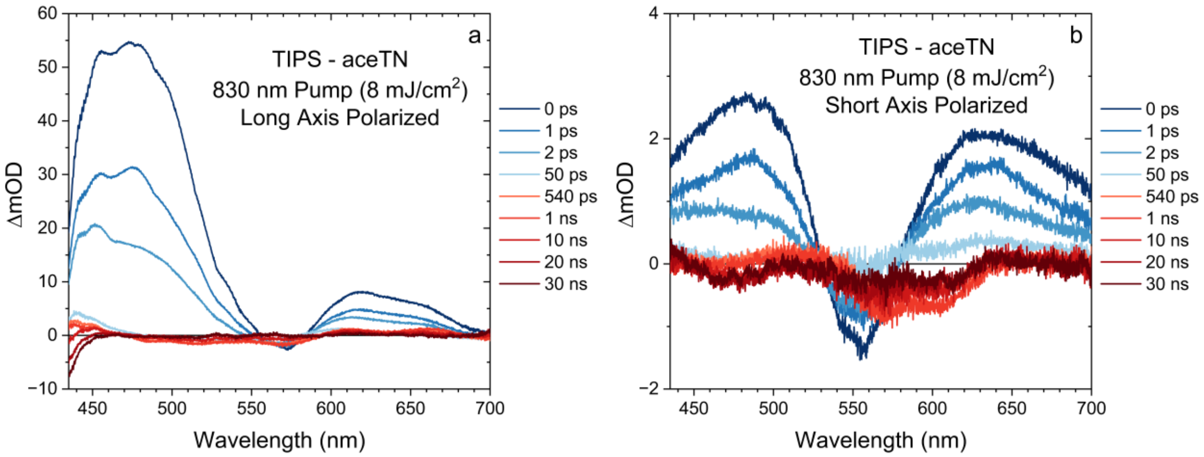

Figure S8. Transient absorption spectra of crystalline TIPS-aceTN with the 830 nm pump and white light probe polarized along the long axis (a) and short axis (b) of the crystal.

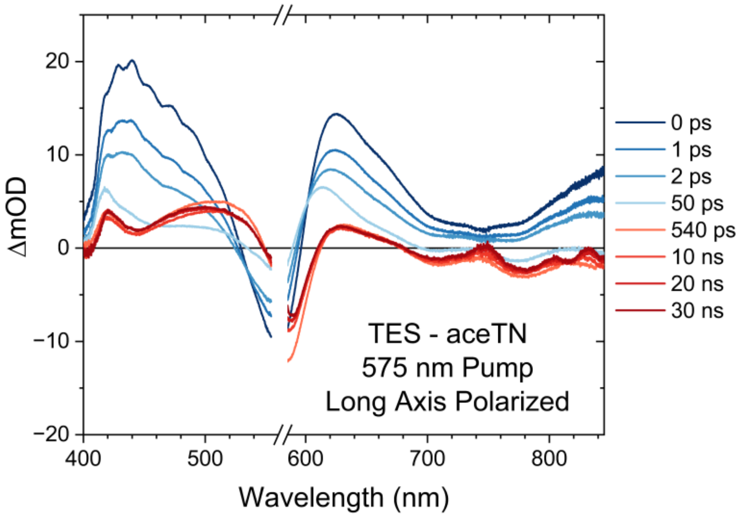

Figure S9. Transient absorption spectra of crystalline TES-aceTN with the 575 nm pump and white light continuum polarized along the long axis of the crystal. The break in the wavelength axis is to remove the pump light from the spectra.

A quantitative analysis of the triplet yield in the crystal TA experiments was made using the same reasoning as for the solution phase data. In solution the triplet absorption coefficient of TES-aceTN was determined from the known absorption (Figure S5b) and the known concentrations since [NMP*] = ^{3}[TES-aceTN]. When expressed as an absorption cross section per molecule (σ_T) we find $\sigma_{T,\ 520nm} = 1.6\times10^{-17}$ cm^2. Unlike the solution experiments σ_T is not known for the solids. However, the lack of strong solution-to-solid spectral shifts which are often observed upon crystallization raises the likelihood that σ_T is of similar magnitude to that determined from our solution measurements. Furthermore, we find that the oscillator strengths of the singlet transitions are the same for solution and crystal, which supports the notion that the same is true for the triplet transitions. If we assume that σ_T is the same in the crystal as in solution, then one calculates, from the limit for differential absorption at 520 nm in the crystal TA experiments, what the maximum density of triplet species, ^{3}[TES-aceTN], could have been following excitation. That number is then compared to the number of excited singlet species, ^{1}[TES-aceTN], created by 415 nm pump laser which is calculated from the known laser fluence and the known molecular absorption coefficient. That results in a value of (^{3}[TES-aceTN] / ^{1}[TES-aceTN]) < 1.7×10^{-3}. As in solution, the triplet yield in the crystals is insignificant.

How Thermal Effects May Influence TA Measurements

Several previous studies have claimed that heating effects caused by the pump laser can dominate the long time TA spectra in molecules similar to those studied here.[12-15] Such thermal effects may be mistaken for triplet TA signatures. We now make a quantitative estimate of likely thermal effects, based on the known equations for temperature rise and heat flow, our experimental conditions, and the known physical properties of the molecules. Then we compare the calculations to the observed TA spectra. The long-time TA spectra and their time dependence are consistent with their being primarily a thermal effect.

From the known laser excitation fluence(J/cm^2) and the known absorption of the sample, the maximum possible temperature rise ΔT_{max} throughout the sample is easily calculated. If all the absorbed laser energy is converted to heat on a timescale short compared to the rate of heat transfer from the crystal to the substrate, then initially the heat distribution in the crystal is $\Delta T(x, t = 0) = \Delta T_{max} e^{-\alpha x}$, where x is the distance

into the crystal from the laser entry face. α(cm^{-1}) is the crystal absorption coefficient at the pump laser wavelength. We initially approximate the heat flow as a 1D problem, which is justified because the thickness of the crystal is much smaller than the diameter of the pump laser beam. The probe laser beam is much smaller in diameter than the pump, so it probes a region of approximately constant fluence in the plane of the sample at the center of the pump laser. The peak energy deposited per laser pulse ΔE_{max}(J/cm^3) = Fluence(J/cm^2) × α(cm^{-1}). $\Delta T_{max} = \Delta E_{max}$(J/cm^3)/$C_p$, where C_p is the heat capacity of the crystal. Although C_p has not been measured for TIPS-aceTN or TES-aceTN, it has been measured for many analogous molecules: C_p(J/(K cm^3)) is 1.4 for tetracene and pentacene[16] and between 1.3 and 1.6 for most organic molecules.[17] In our calculations we assumed C_p = 1.4. In our experiments ΔTmax varied depending on the fluence of the pump pulse and the wavelength since α varies by a factor of 6.8 from the shortest wavelength used (403 nm) to the longest (830 nm).

With the initial ΔT(x, t = 0) known from above, ΔT(x, t > 0) was calculated from heat flow equation:

$$\frac{\partial T}{\partial t} = \kappa \nabla^2 T \qquad \text{(Eqn. 5)}$$

or in 1D:

$$\frac{\partial T}{\partial t} = \kappa \frac{\partial^2 T}{\partial x^2} \qquad \text{(Eqn. 6)}$$

We specifically employed the solution given as Eq 5, page 84 in Carslaw and Jaeger:[18]

$$\Delta T(x,t) = \frac{2}{L}\sum_{n=0}^{\infty} e^{\left(-\frac{\kappa a^2 t}{4L^2}\right)} \cos\left(\frac{ax}{2L}\right) \int_0^L \Delta T(x, t=0) \cos\left(\frac{ax}{2L}\right)\, dx \qquad \text{(Eqn. 7)}$$

where κ is the thermal diffusivity (K / Cp), a = (2n+1)π and L is the thickness of the crystal. The sum over n is from 0 to infinity.

It is frequently said that thermal decay time is the n=0 term in the sum, $\tau = 4L^2/\kappa\pi^2$. While true as $t \to \infty$, we sometimes had to include terms out to n ≈200 to get convergence at early relevant times. Our simplest calculations assumed that after the initial laser pulse no heat flowed in or out at the top (air) surface (insulated condition). The substrate was assumed to have a thermal conductivity fast (high) compared to the crystal, so the substrate temperature remained at room T.

Regrettably there are no data for the thermal conductivity of TES-aceTN or TIPS-aceTN. Thermal conductivities of conjugated polymers are ≈0.1 to 0.2 W/(m·K).[12] Room temperature values for thermal conductivity K (W/(m·K)) have been published for related molecules as crystals and amorphous thin films. For rubrene crystals, K values in the range 0.074 W/(m·K) to 0.5 W/(m·K) have been reported.[19, 20] Since thermal conductivity depends upon crystalline spatial arrangements, it is expected that K values may be different in plane vs in transverse direction. Thin films of pentacene have K ≈ 0.5 W/(m·K).[21] Thin films of 2, 8-difluoro-5, 11-bis(triethylsilylethynyl)anthradithiophene and TIPS-pentacene have K in the range 0.1 W/(m·K) to 0.14 W/(m·K).[22, 23] Crystalline TIPS-pentacene along the *a*-axis has K= 1.6 W/(m·K).[22] Amorphous polyethylene has K = 0.34 W/(m·K).[17] Given the significant range of K values, we expect K values for TIPS-aceTN or TES-aceTN crystals to be in the range 0.1 W/(m·K) to 1.0 W/(m·K).

Below are simulations for ΔT(x, t) for our samples for a lower (K = 0.2 W/(m·K)) and higher (K = 0.8 W/(m·K)) value of K. This simulates our film of 0.8 µm thickness when it is excited at a laser wavelength of 403 nm (used in all our long time (t > 30 ns) TA data) and a typical fluence of 0.81 mJ/cm^2.

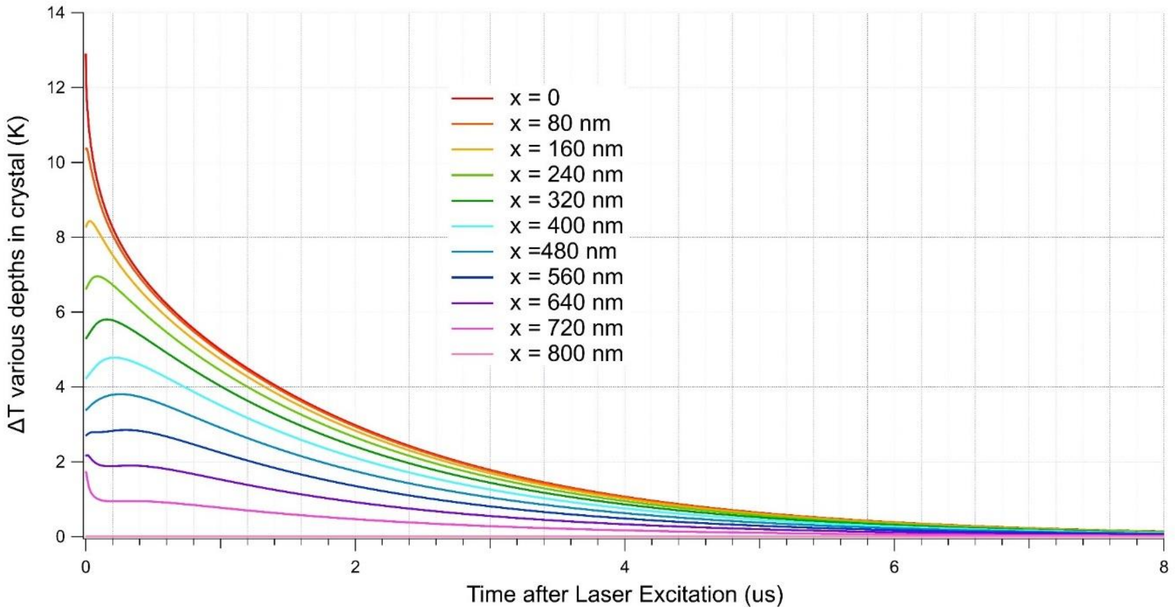

Figure S10. Graph of calculated temperature rise vs time after laser excitation for various depths into a **TIPS-aceTN** crystal. x = 0 at laser entrance face; x = 0.8 μm at substrate interface. Assumed thermal conductivity is K (W/(m·K)) = 0.2.

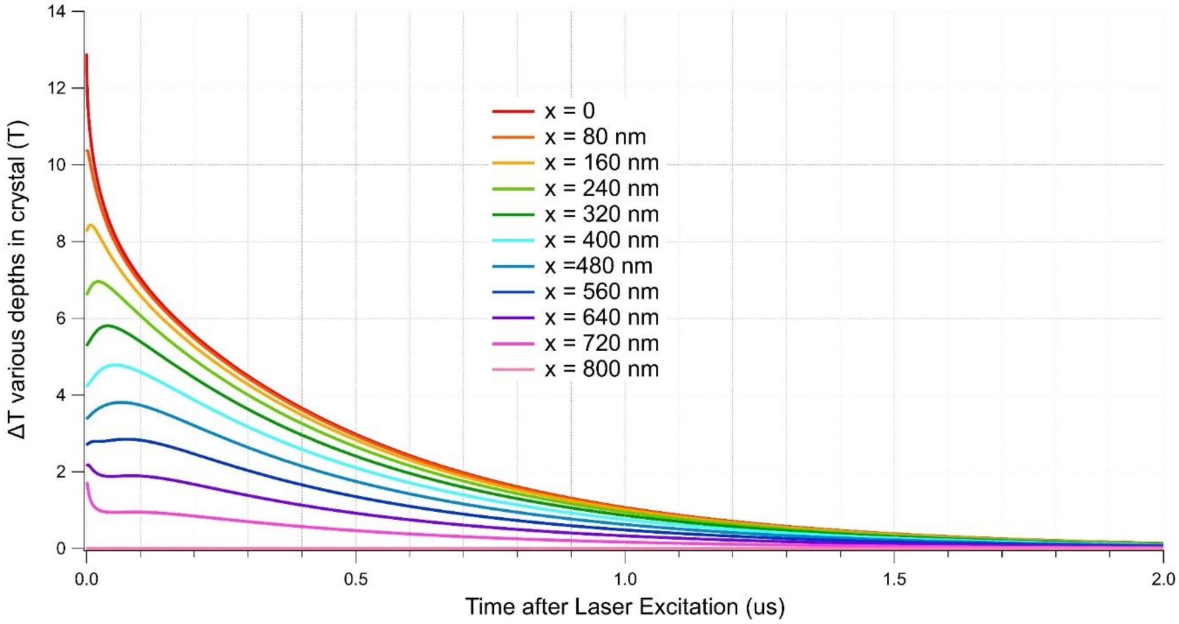

Figure S11. Graph of calculated temperature rise vs time after laser excitation for various depths into a **TES-aceTN** crystal. x= 0 is laser entrance face; x = 0.8 μm is substrate interface. Assumed thermal conductivity is K (W/(m·K)) = 0.8. Note the change in horizontal time axis between Fig S10 and Fig S11.

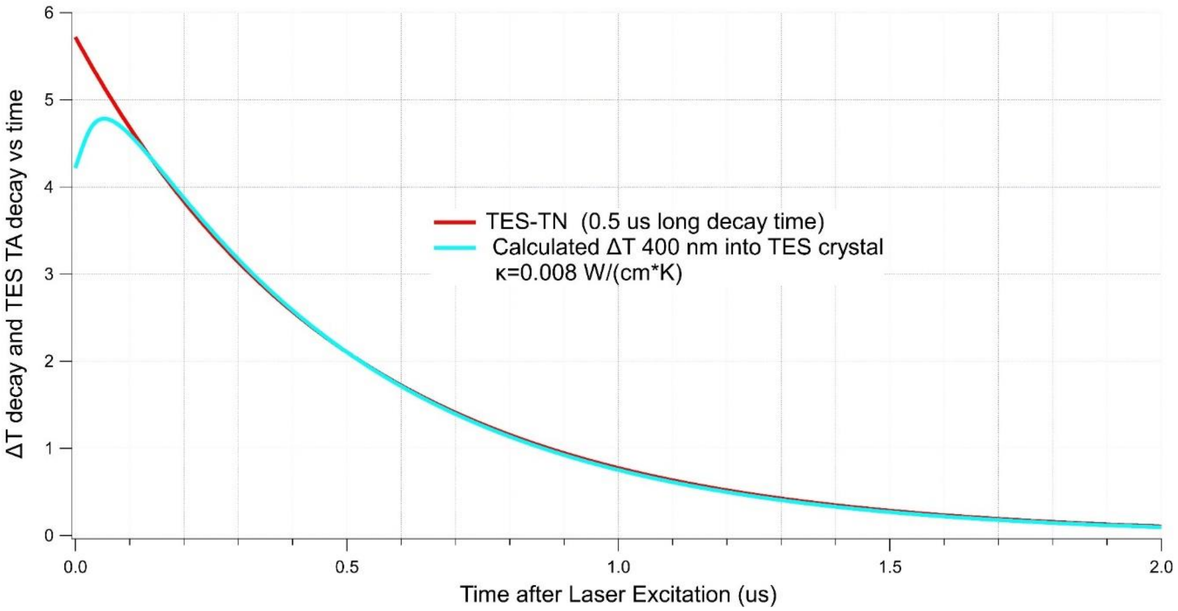

Figure S12. T near the middle of the crystal (0.4 μm down) for the K = 0.008 W/(cm·K) simulation compared to the observed long time TA decay of **TES-aceTN** (τ =0.5 μs). Vertical scale on TA decay is arbitrary. It is seen that the time dependence observed is quite consonant with the simulation using a K value in the expected range.

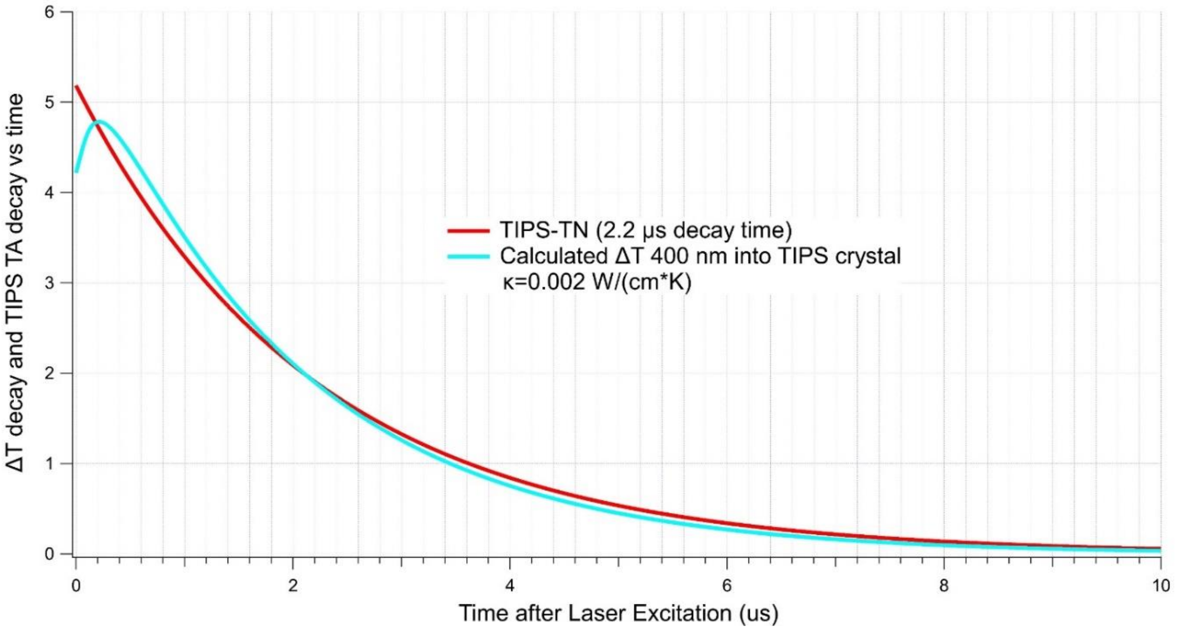

Figure S13. T near the middle of the crystal (0.4 μm down) for the K = 0.002 W/(cm·K) simulation compared to the observed long time TA decay of **TIPS-aceTN** (τ = 2.2 μs). Vertical scale on TA decay is arbitrary. It is seen that the time dependence observed is quite consonant with the simulation using a K value in the expected range. Since the crystal packing is different between TIPS and TES, a difference in K would be expected.

The initial somewhat odd T distribution is easily understood. The penetration depth of the 405nm pump laser is 357 nm. T at the top of the film shows very fast decay as heat flows into the lower crystal but obviously receives no heat from above. Further into the crystal, T(x,t) is a balance between heat flowing in from above and heat flowing down into the substrate. The bottom layer (L = 0.8 µm) on the substrate is kept at the substrate T, assumed to remain at room T (i.e. K for substrate > K for crystal).

The comparison of TA spectra to thermal-induced spectral changes is shown in Figure S14. Although the calculated value of ΔT(x = 0.4 µm, t = 200 ns) is ≈4.7 K, we did not obtain thermal spectra at that low value of ΔT. However, if the thermal spectra were to scale linearly with ΔT, then the observed amplitude for the TA spectra would be similar to that of the thermal spectra.

Figure S15 shows the sensitivity of the TA decays to substrate (SiO_2 vs sapphire). There is no evidence that the decay is faster on sapphire (higher thermal conductivity) than on SiO_2. Previous studies where such an effect was seen[12-15] involved films ≈100 nm thick. Our thicker 0.8 µm crystals are much less sensitive to substrate conductivity than those thinner films.

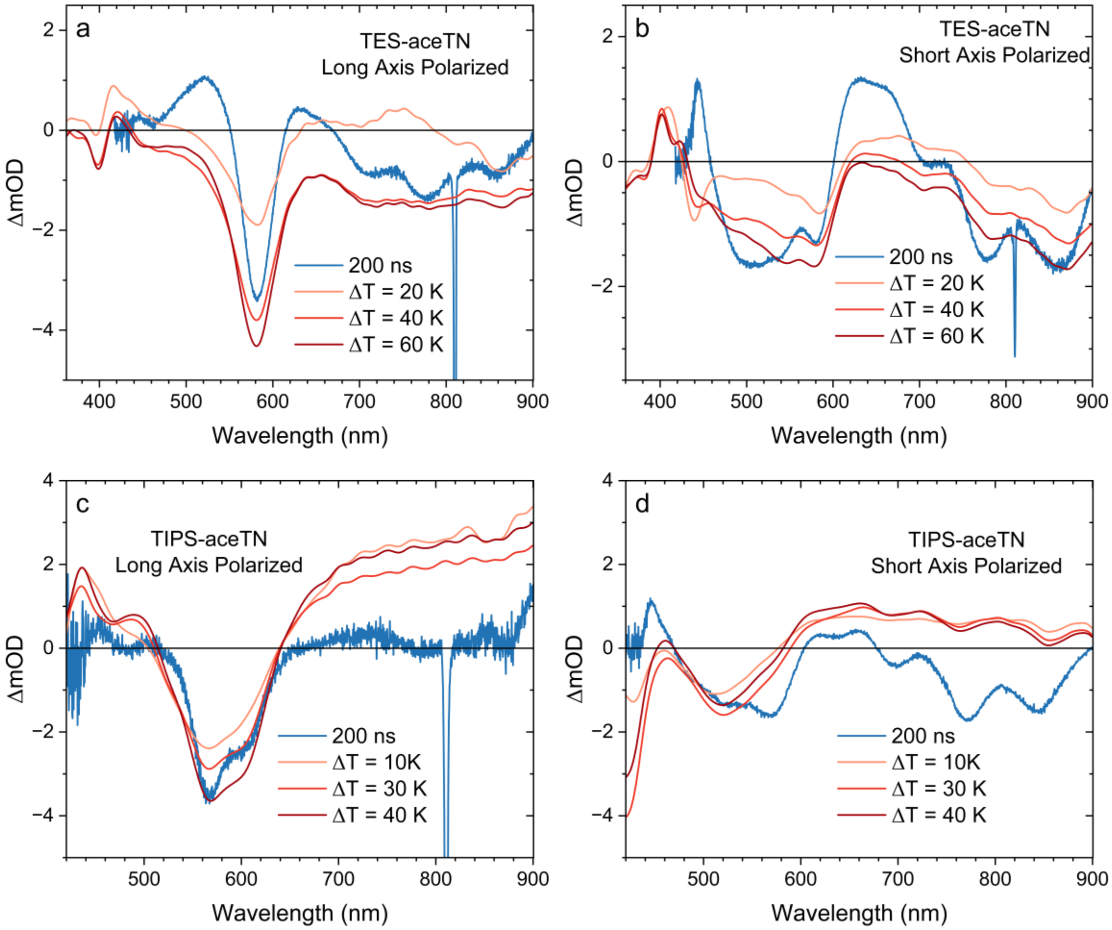

Fig S14. Figure above compares the spectral changes observed for TES and TIPS in the purely thermal experiment to the TA results at t = 200 ns (second tick in on the time axis in Fig S11). The 200 ns spectrum has been scaled vertically. At 200ns the calculated ΔT(x = 0.4 µm, t = 200ns) is ≈4.7K. The experimental thermal spectra in the ΔT = 20 K to 40K range are in modest agreement with the experimental TA spectrum at 200 ns.

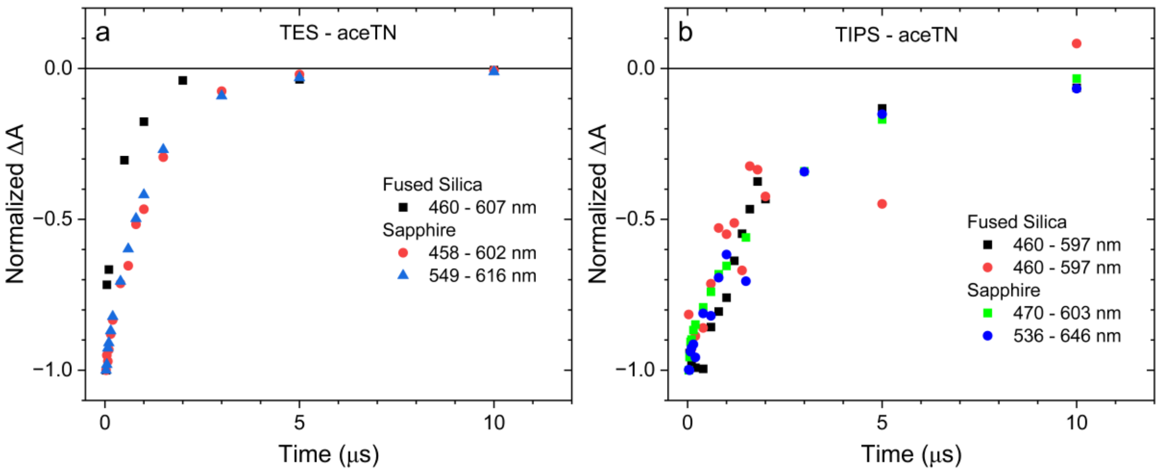

Fig S15. Normalized decays taken with the laser diode showing the dynamics for the thermal artifacts on sapphire and fused silica for (a) **TES-aceTN** and (b) **TIPS-aceTN**.

Our crystal data do not prove that all the pump-induced long-time spectral changes are a thermal effect. The timescales for decay of long-time TA and ΔT are consistent, and the spectral agreement is modest. In solution, we established that triplet formation was insignificant with triplets formed per excited singlets in TIPS-aceTN molecules being $<1.4 \times 10^{-3}$. Since σT is unknown we cannot prove that the crystalline triplet yield is equally small; however, we argue that triplet formation is inconsequential. Therefore, it is extremely unlikely that triplet formation gives rise to the long-time TA signal and is instead consistent with a thermal effect.

Redox Properties

The redox properties of the TES-derivatives of aceacenes and acetetracenes were determined by cyclic voltammetry (CV) and differential pulse voltammetry (DPV). CV shows a reduction that is fully reversible, occurring at -1.45 V for **TES-aceAN** and -1.11 V for **TES-aceTN** (versus the internal ferrocene standard). The smaller **TES-aceAN** exhibits a second reduction within the solvent electrochemical window, an irreversible reduction occurring at -2.1 V. The oxidation of both **TES-aceAN** and **TES-aceTN** is irreversible and at 0.89 V and 0.68 V respectively. These redox events equate quite well with the optical data and theory; the gaps between occupied and unoccupied energy levels are smaller in acetetracene **TES-aceTN** than in **TES-aceAN**, which is due to both higher energy HOMO and lower energy LUMO. The electrochemical HOMO-LUMO gaps of **TES-aceAN** and **TES-aceTN** were measured as 2.29 eV and 1.73 eV respectively, equating to 541 nm and 717 nm. These electrochemical gaps are slightly larger (blue-shifted) compared to the onset of the broad absorption bands in Figure 1.

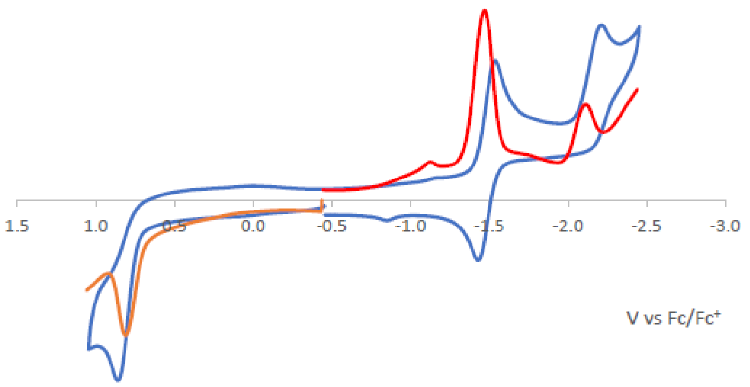

Figure S16. Cyclic voltammetry (blue) and differential pulse voltammetry (orange and red) data measured for **TES-aceAN** in 100 mol/m^3 tetrabutyl ammonium hexafluorophosphate electrolyte in dichloromethane at 298 K, using a scan rate of 50 mV/s. Data was referenced to Ferrocene internal standard (not shown in these scans).

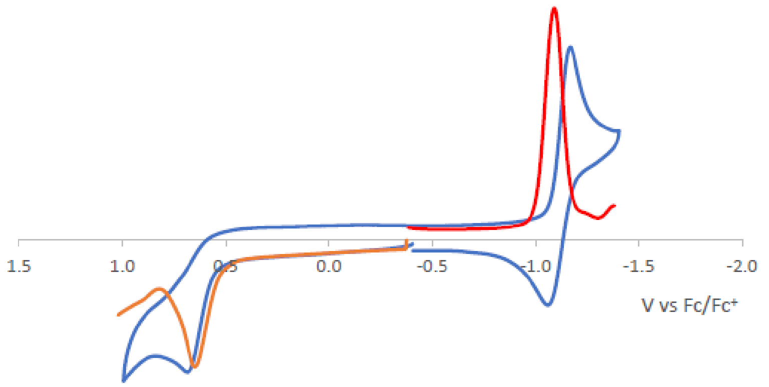

Figure S17. Cyclic voltammetry (blue) and differential pulse voltammetry (orange and red) data measured for **TES-aceTN** in 100 mol/m^3 tetrabutyl ammonium hexafluorophosphate electrolyte in dichloromethane at 298 K, using a scan rate of 50 mV/s. Data was referenced to Ferrocene internal standard (not shown in these scans).

Theoretical Calculations

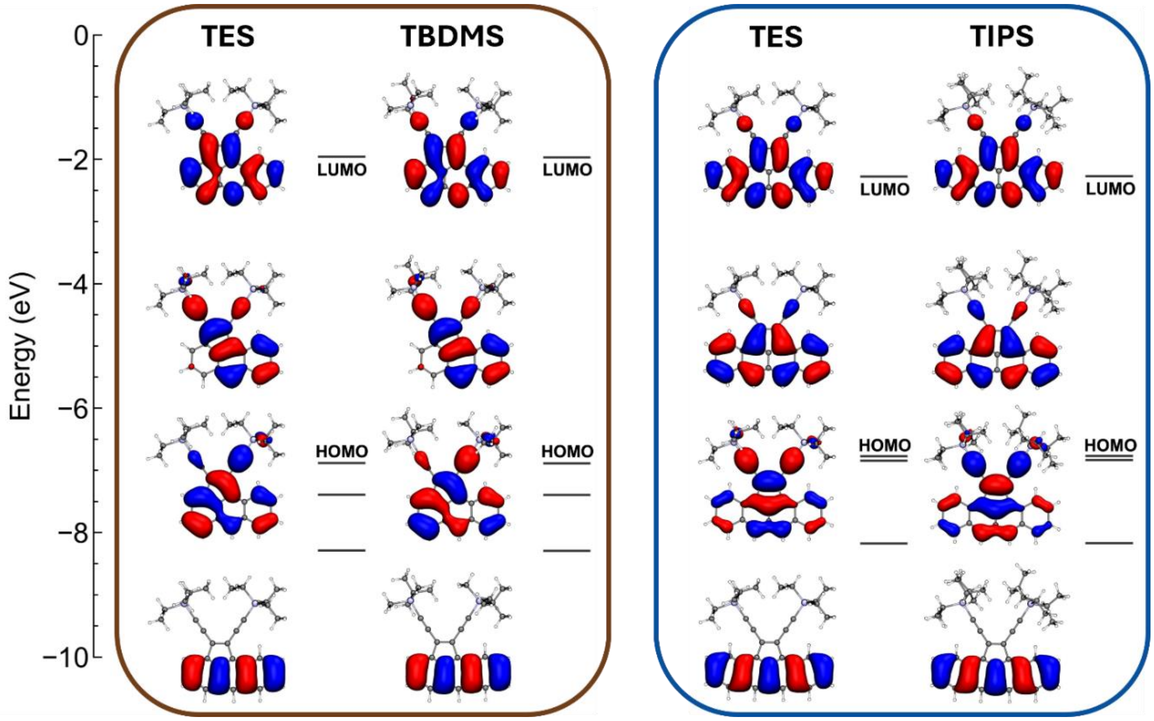

Figure S18. Frontier MOs of the aceacenes (left) and acetetracenes (right) in a toluene PCM at TD-CAM-B3LYP-D3(BJ)/6-311++G** level of theory (isovalue = 0.02).

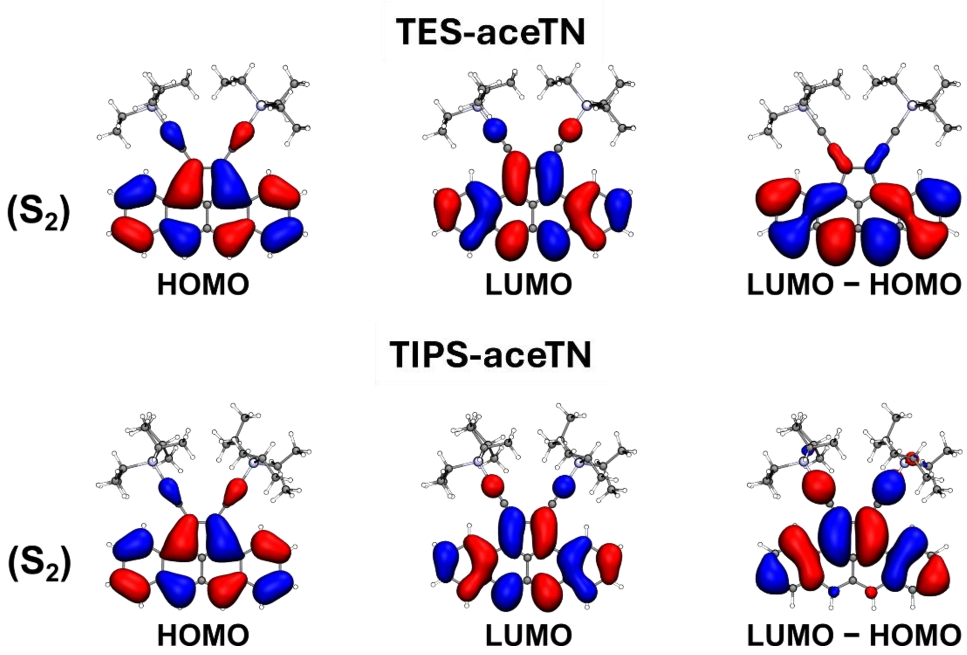

Figure S19. Natural transition orbitals (NTOs) from the second singlet excited state (S_2) of **TES-aceTN** (top) and **TIPS-aceTN** (bottom) at TD-CAM-B3LYP-D3(BJ)/6-311++G** level of theory. Difference densities (LUMO minus HOMO) show how **TES-aceTN** and **TIPS-aceTN** differ and agree with the transition dipole along the y-axis (isovalue = 0.02)

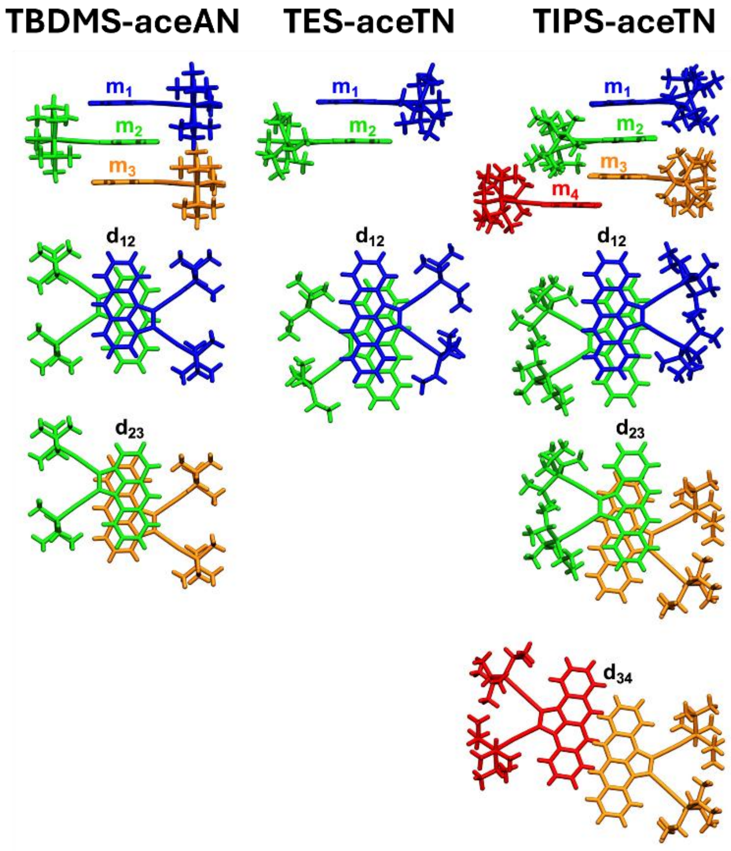

Figure S20. Monomers and nearest neighbor dimers from **TBDMS-aceAN**, **TES-aceTN**, and **TIPS-aceTN** used in the electronic coupling, reorganization energy, and TD-DFT calculations. Molecule designations match those highlighted in Figure 3 of the main text.

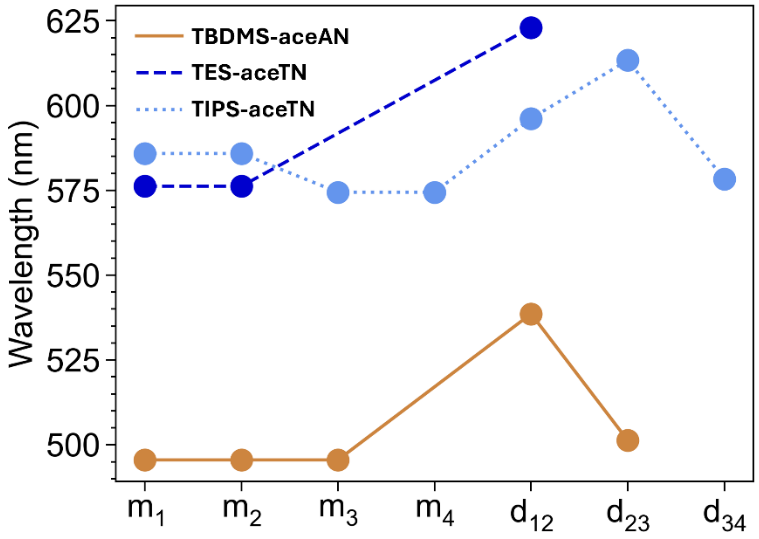

Figure S21. S_1 excitation wavelengths (nm) as a function of monomers and dimers extracted from **TBDMS-aceAN**, **TES-aceTN**, and **TIPS-aceTN** crystal structures at TD-CAM-B3LYP-D3(BJ)/6-311++G** level of theory.

Table S4. Binding energies between adjacent molecules extracted from **TBDMS-aceAN**, **TES-aceTN**, and **TIPS-aceTN** at CAM-B3LYP-D3(BJ)/6-311++G** level of theory.

	$E_{b,12}$ [eV]	$E_{b,23}$ [eV]	$E_{b,34}$ [eV]
**TBDMS-aceAN**	-1.148	-0.993	---
**TES-aceTN**	-1.401	---	---
**TIPS-aceTN**	-1.371	-1.392	-0.381

Table S5. Plane-to-plane distances in π-stacked molecules in crystal structures of **TBDMS-aceAN**, **TES-aceTN**, and **TIPS-aceTN**.

	$d_{b,12}$ [Å]	$d_{b,23}$ [Å]	$d_{b,34}$ [Å]
**TBDMS-aceAN**	3.38	3.38	---
**TES-aceTN**	3.37	---	---
**TIPS-aceTN**	3.32	3.38	2.86

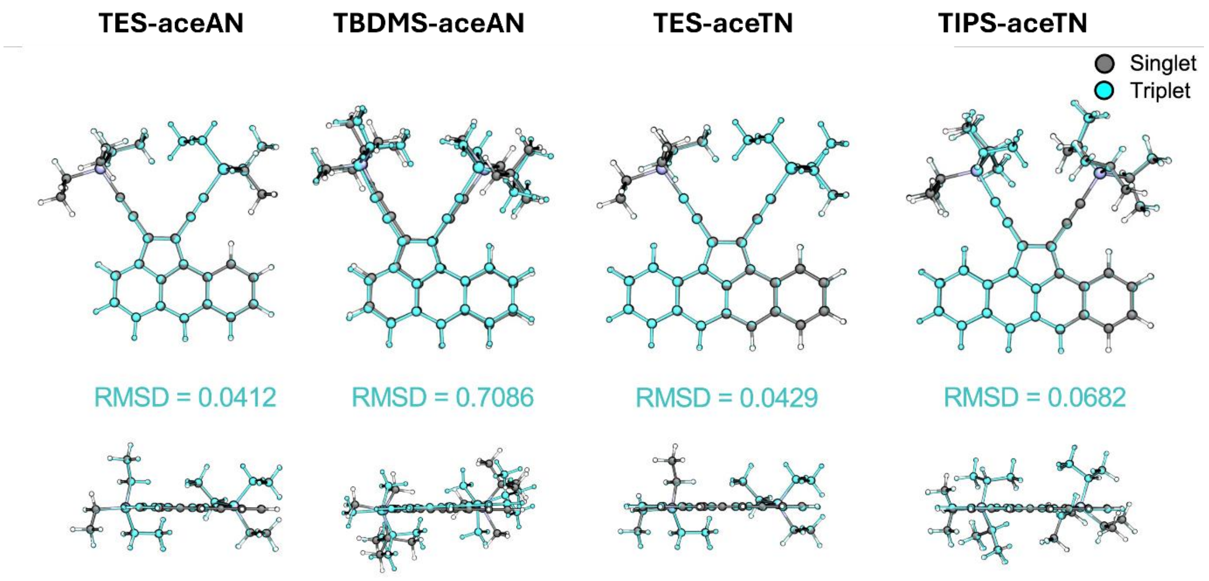

Figure S22. Optimized singlet (grey) and triplet (cyan) geometries of **TES-aceAN, TBDMS-aceAN**, **TES-aceTN**, and **TIPS-aceTN** at CAM-B3LYP-D3(BJ)/6-311++G**/PCM(toluene) level of theory. Root mean square deviations (RMSD) are in units of angstrom.

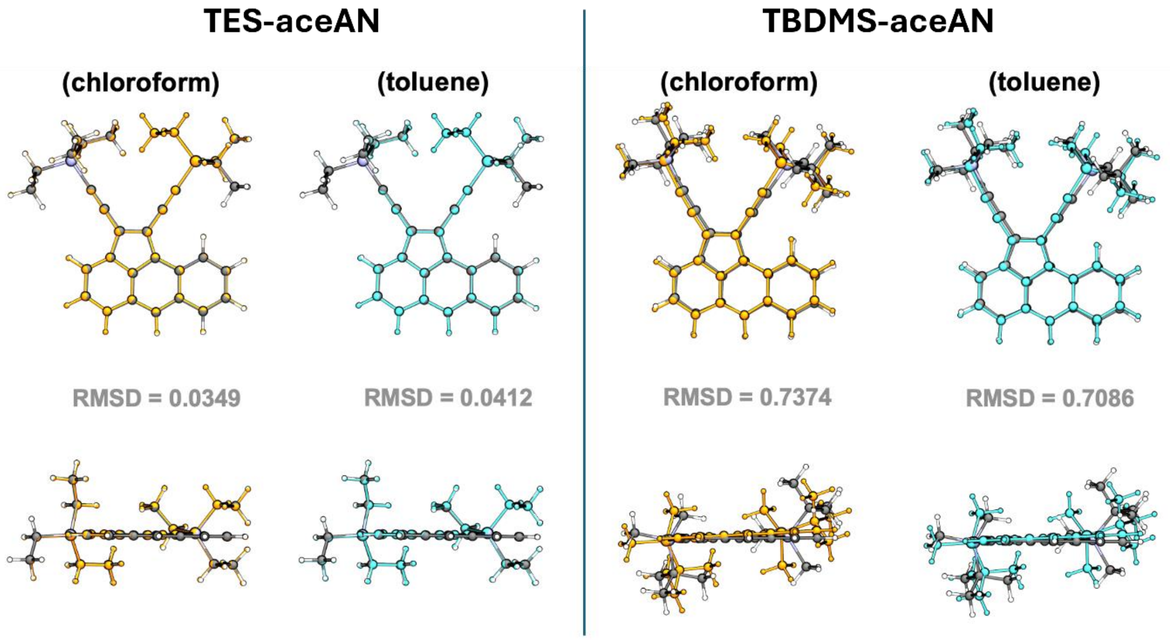

Figure S23: Optimized singlet (grey) and triplet geometries (orange or cyan) of **TES-aceAN** and **TBDMS-aceAN** at CAM-B3LYP-D3(BJ)/6-311++G** level of theory in chloroform (orange) and toluene (cyan) PCM. Root mean square deviations (RMSD) are in units of angstrom.

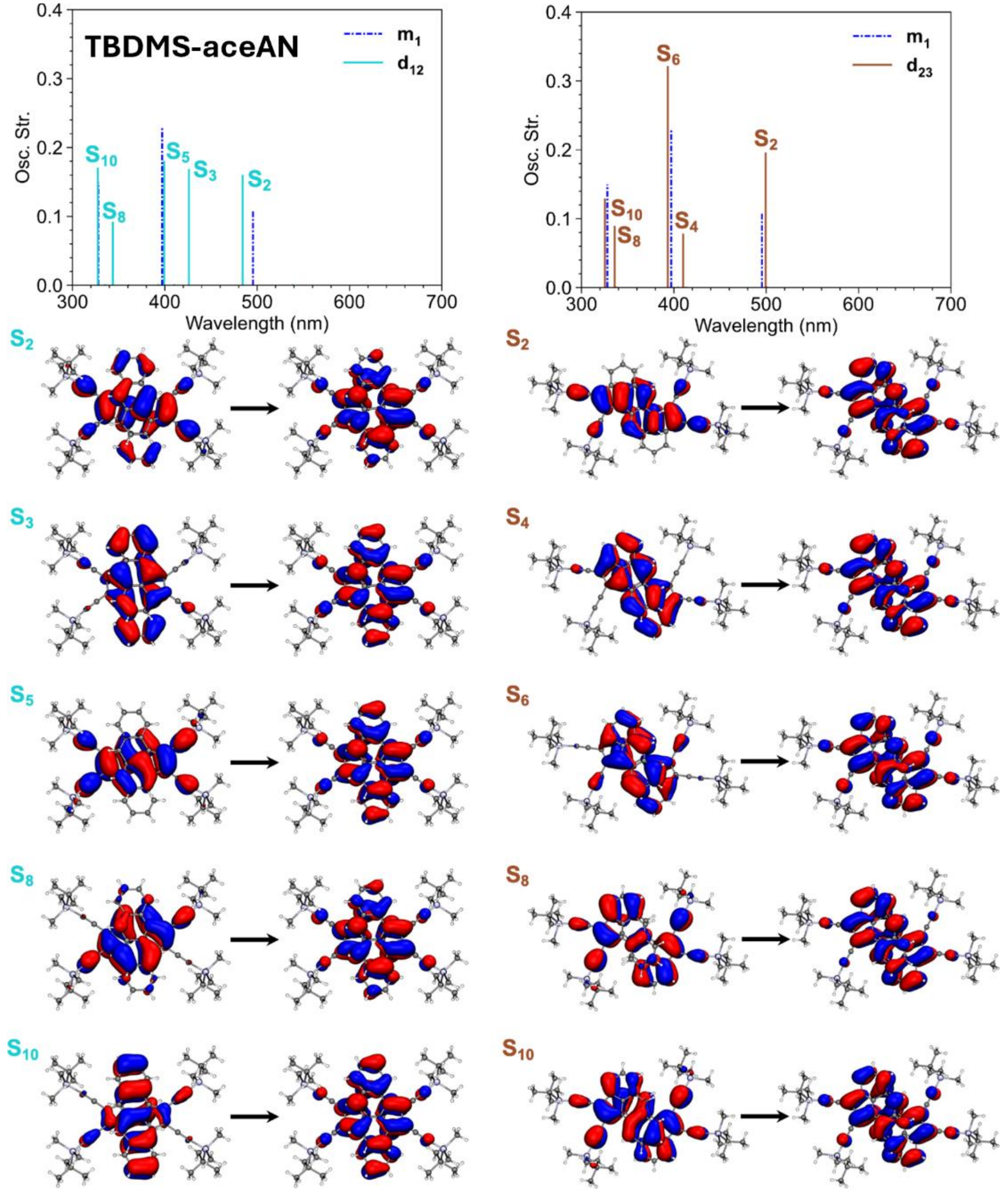

Figure S24. Natural transition orbitals (NTOs) of excitations from 300 – 700 nm for dimer d_{12} and d_{23} of **TBDMS-aceAN**. CAM-B3LYP-D3(BJ)/6-311++G**/PCM(toluene).

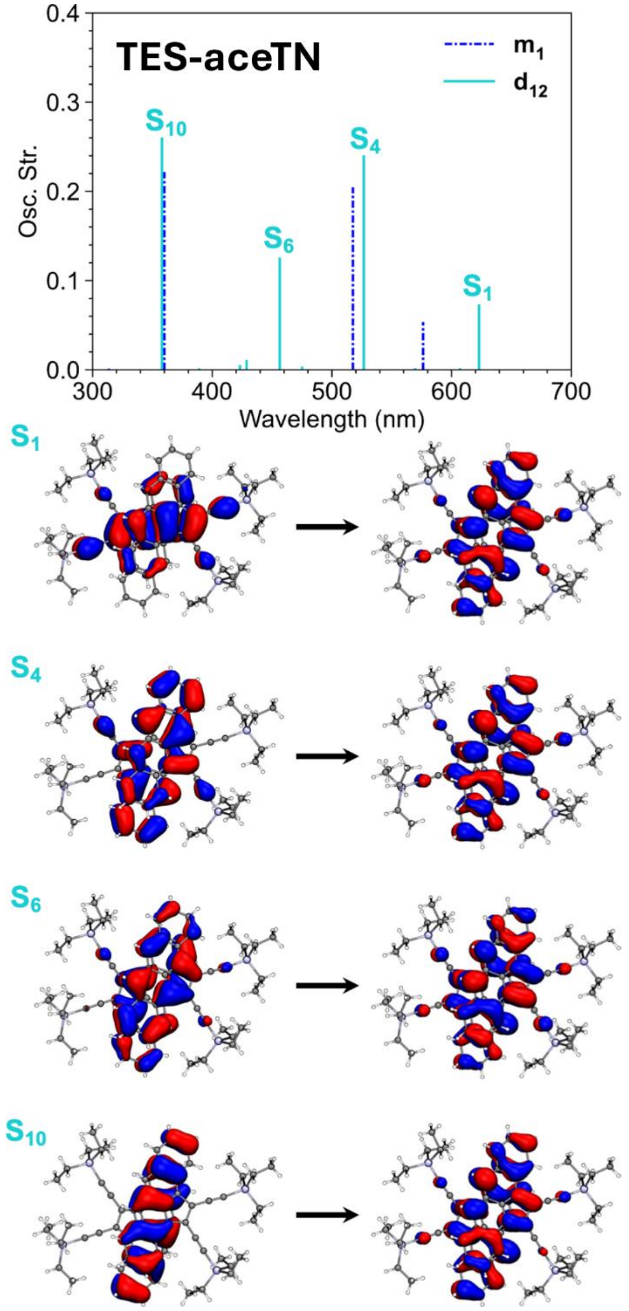

Figure S25. Natural transition orbitals (NTOs) of excitations from 300 – 700 nm for dimer d_{12} of **TES-aceTN**. CAM-B3LYP-D3(BJ)/6-311++G**/PCM(toluene).

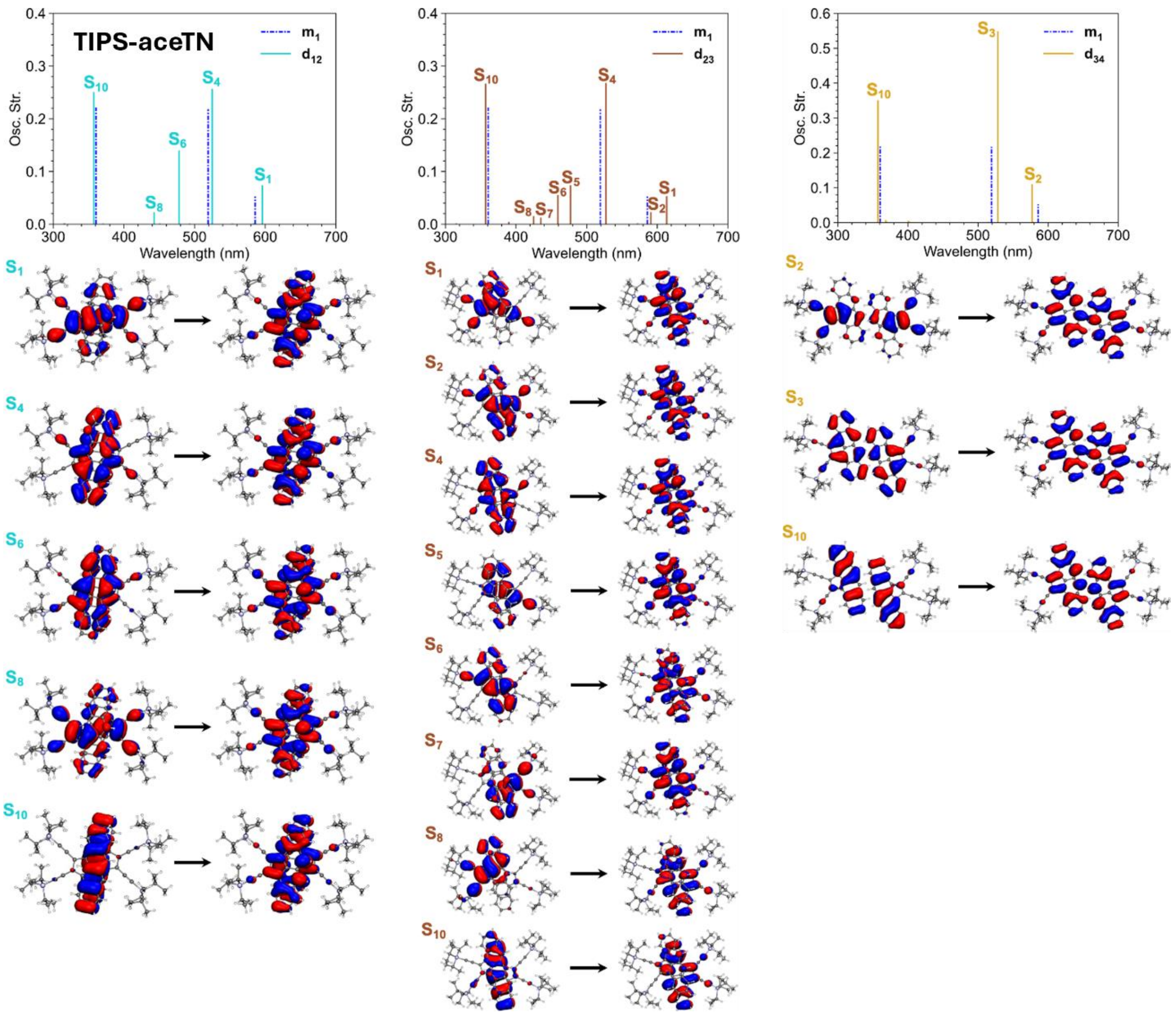

Figure S26. Natural transition orbitals (NTOs) of excitations from 300 – 700 nm for dimer d_{12}, d_{23}, and d_{34} of **TIPS-aceTN**. CAM-B3LYP-D3(BJ)/6-311++G**/PCM(toluene).

Copies of Characterization Spectra

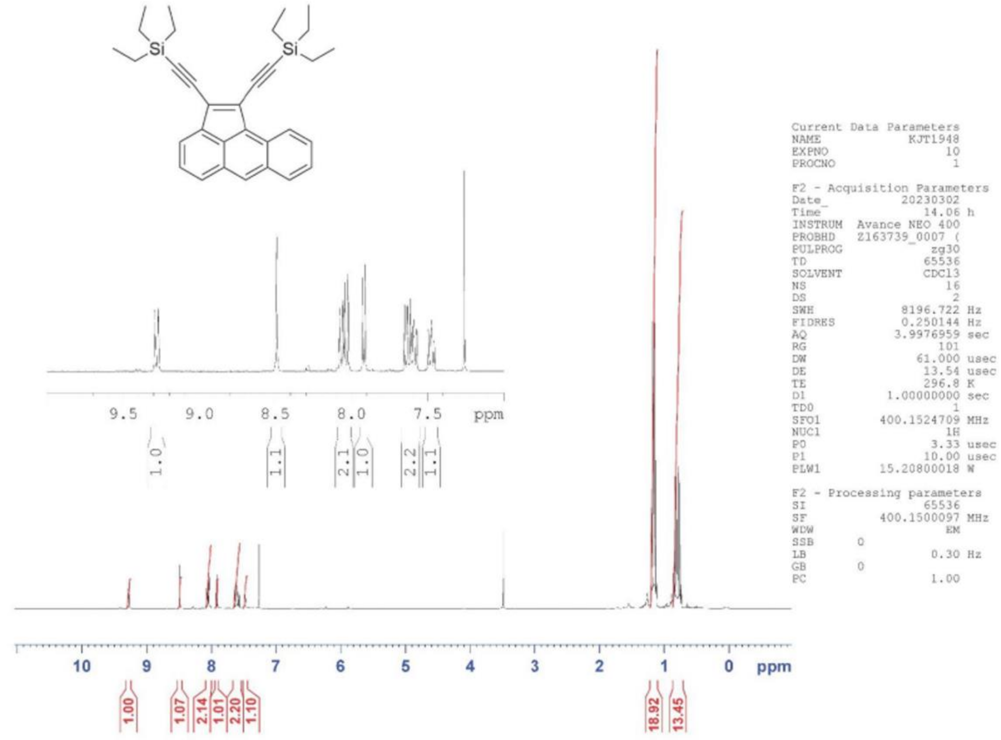

Figure S27. 400 MHz ^{1}H NMR spectrum of **TES-aceAN** measured in $CDCl_3$ at 298K.

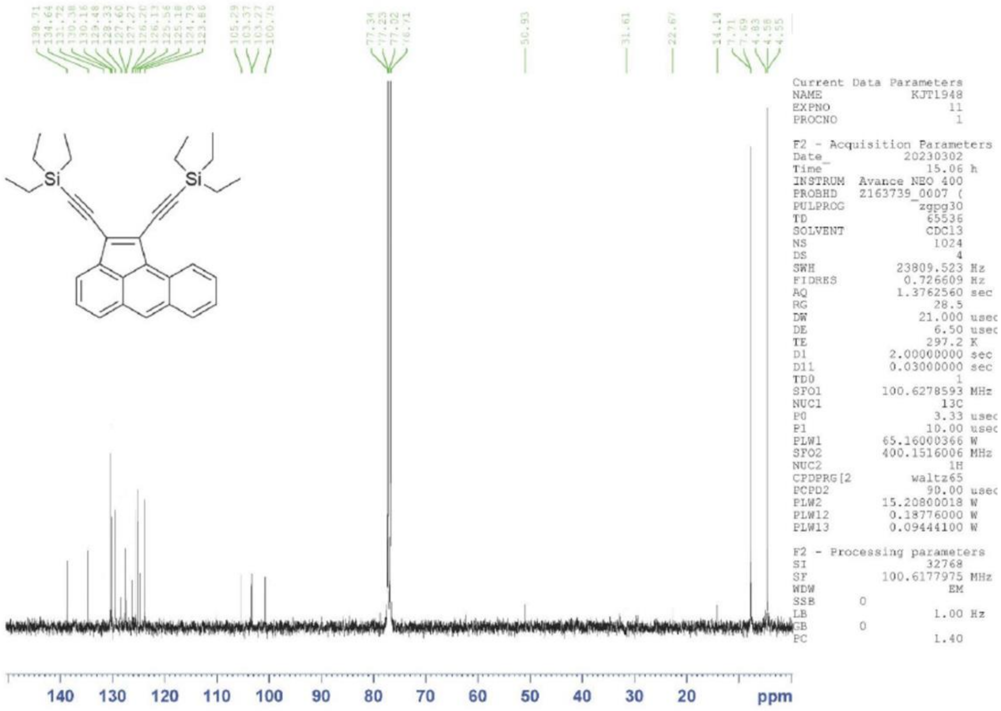

Figure S28. 100 MHz ^{13}C NMR spectrum of **TES-aceAN** measured in $CDCl_3$ at 298K.

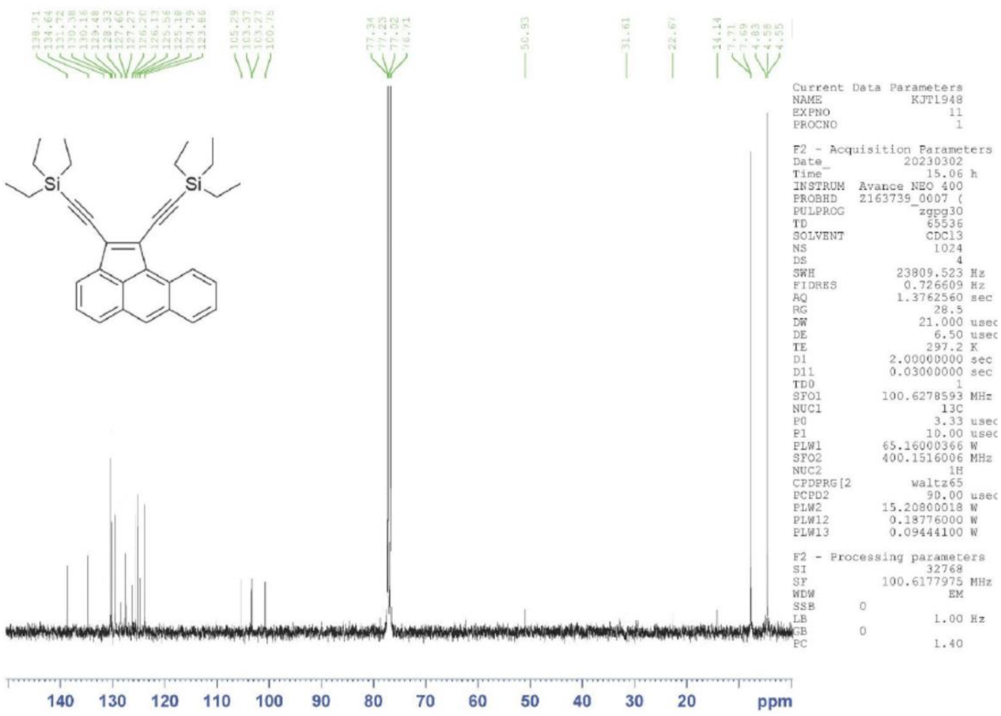

Figure S29. 400 MHz ^{1}H NMR spectrum of **TBDMS-aceAN** measured in $CDCl_3$ at 298K.

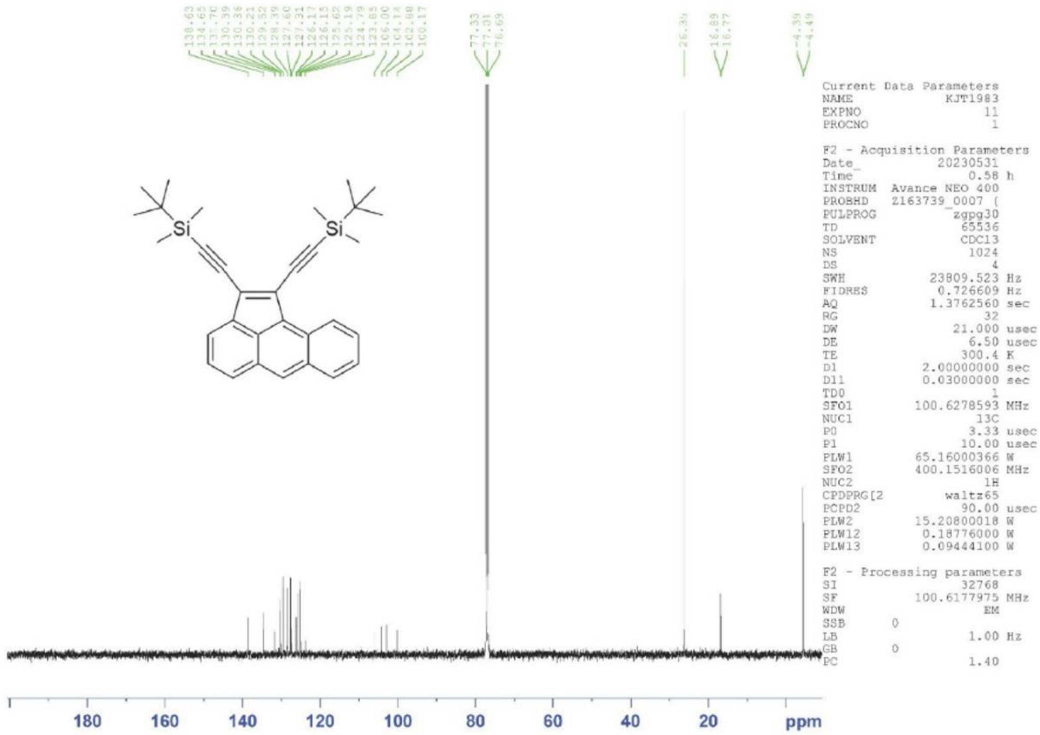

Figure S30. 100 MHz ^{13}C NMR spectrum of **TBDMS-aceAN** measured in $CDCl_3$ at 298K

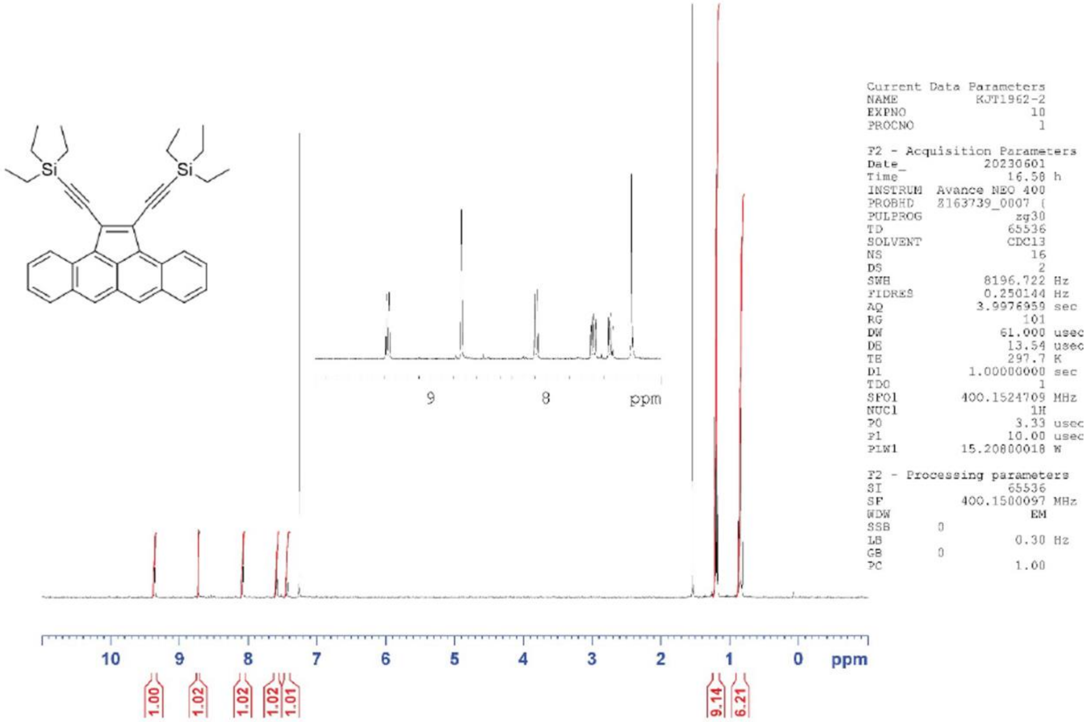

Figure S31. 400 MHz ^{1}H NMR spectrum of **TES-aceTN** measured in $CDCl_3$ at 298K.

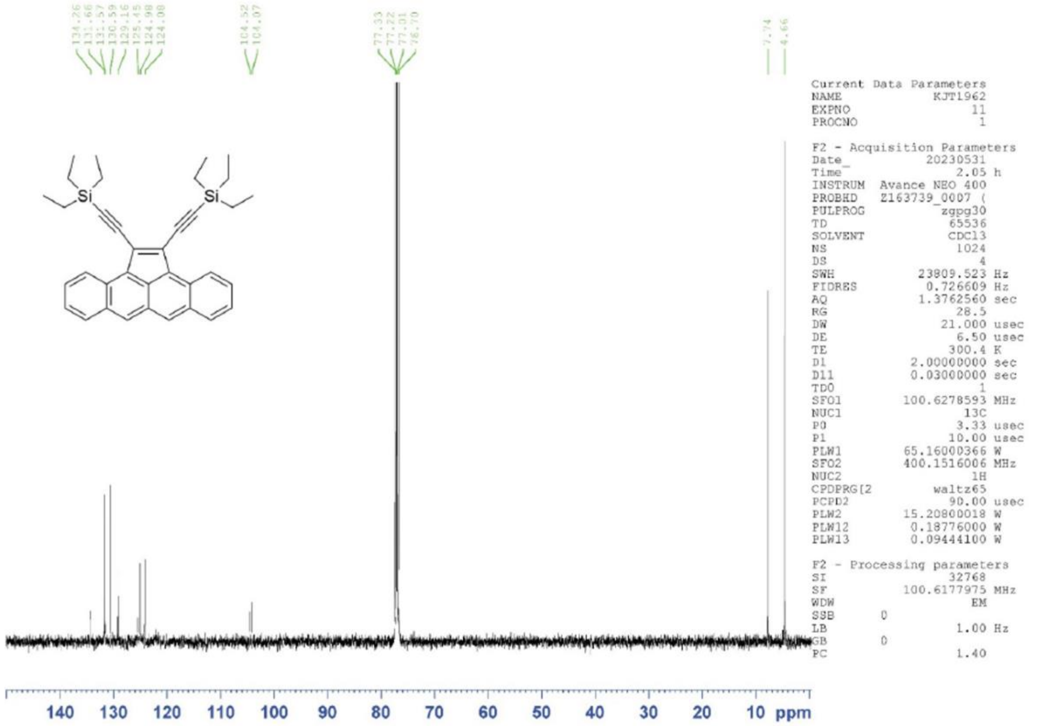

Figure S32. 100 MHz ^{13}C NMR spectrum of **TES-aceTN** measured in $CDCl_3$ at 298K.

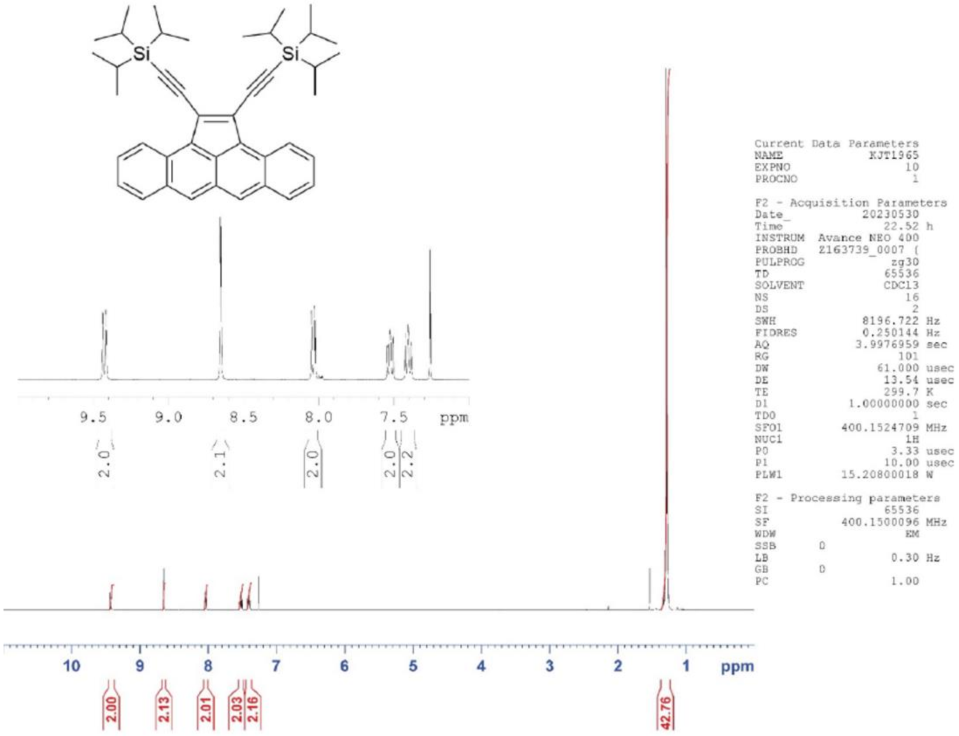

Figure S33. 400 MHz ^{1}H NMR spectrum of **TIPS-aceTN** measured in $CDCl_3$ at 298K

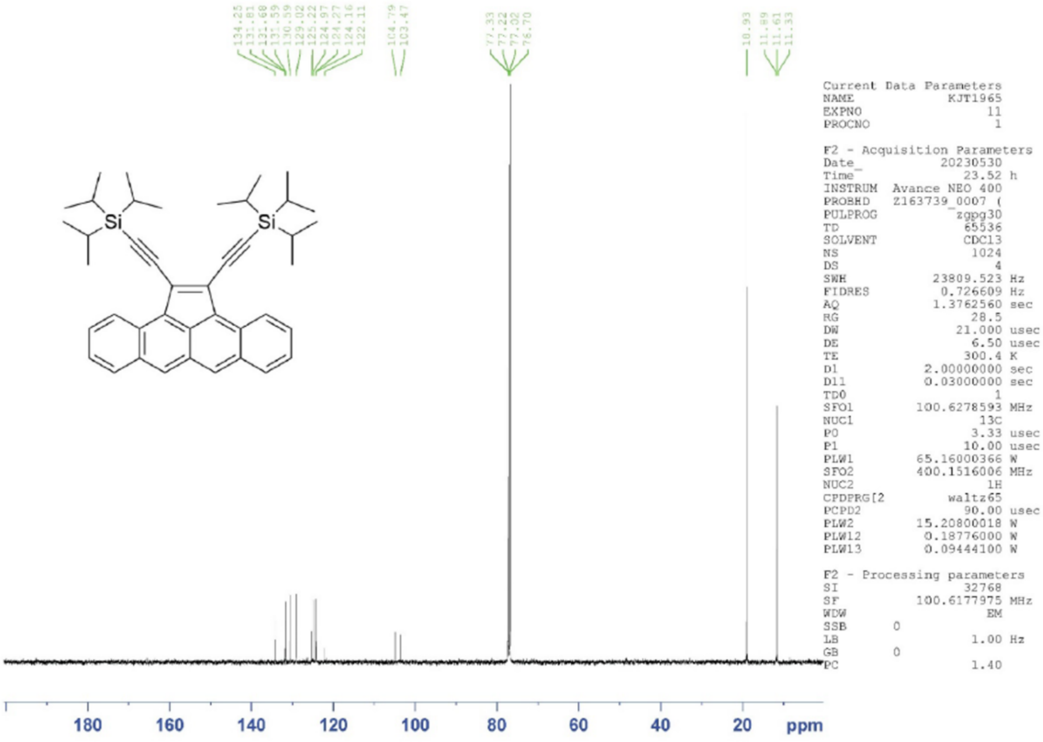

Figure S34. 100 MHz ^{13}C NMR spectrum of **TIPS-aceTN** measured in $CDCl_3$ at 298K.

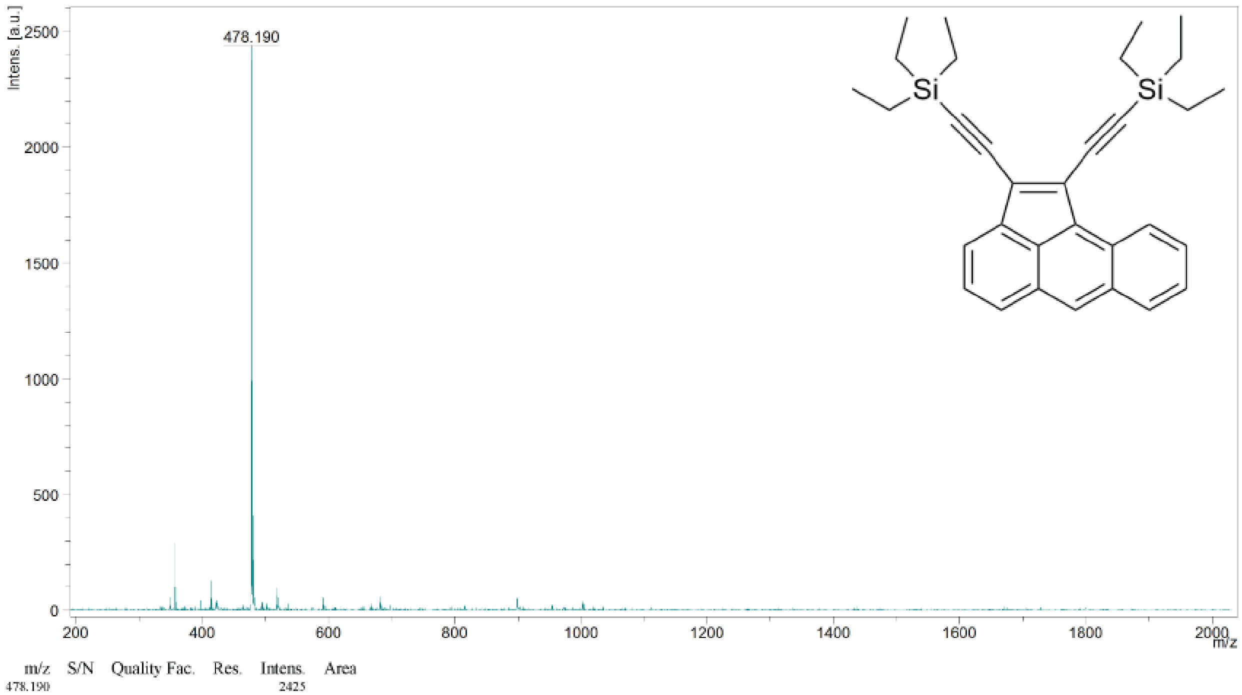

Figure S35. MALDI-MS spectrum of **TES-aceAN**.

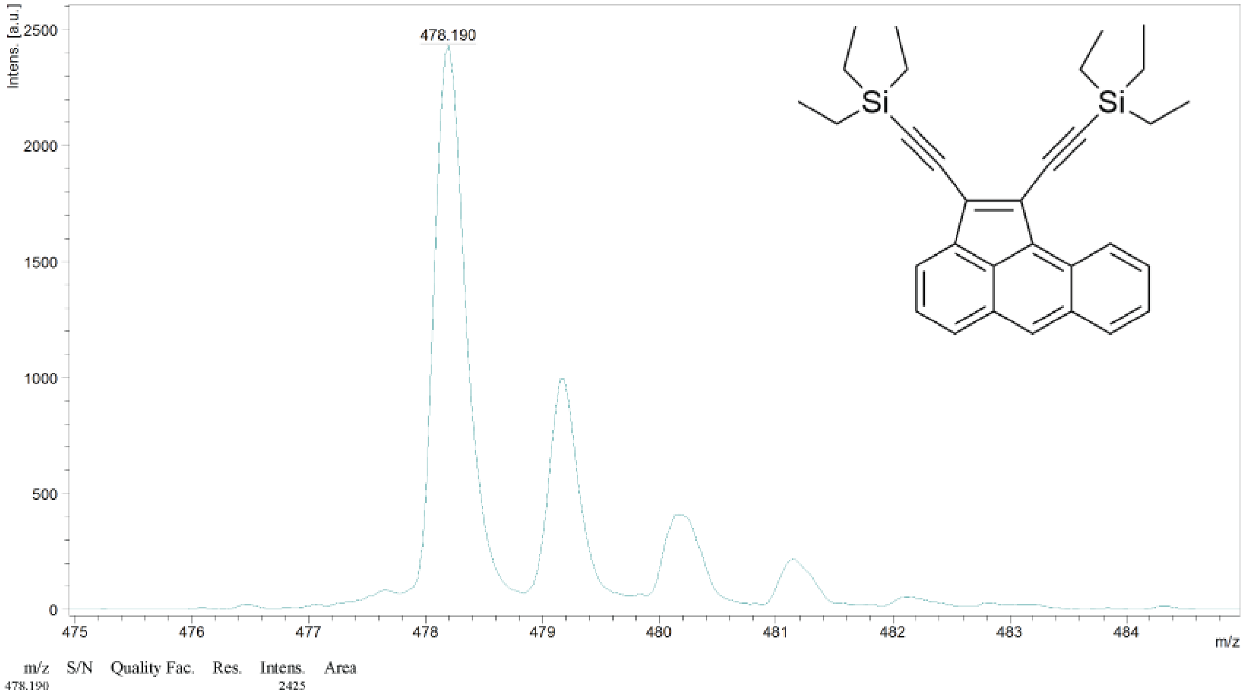

Figure S36. MALDI-MS spectrum of **TES-aceAN**.

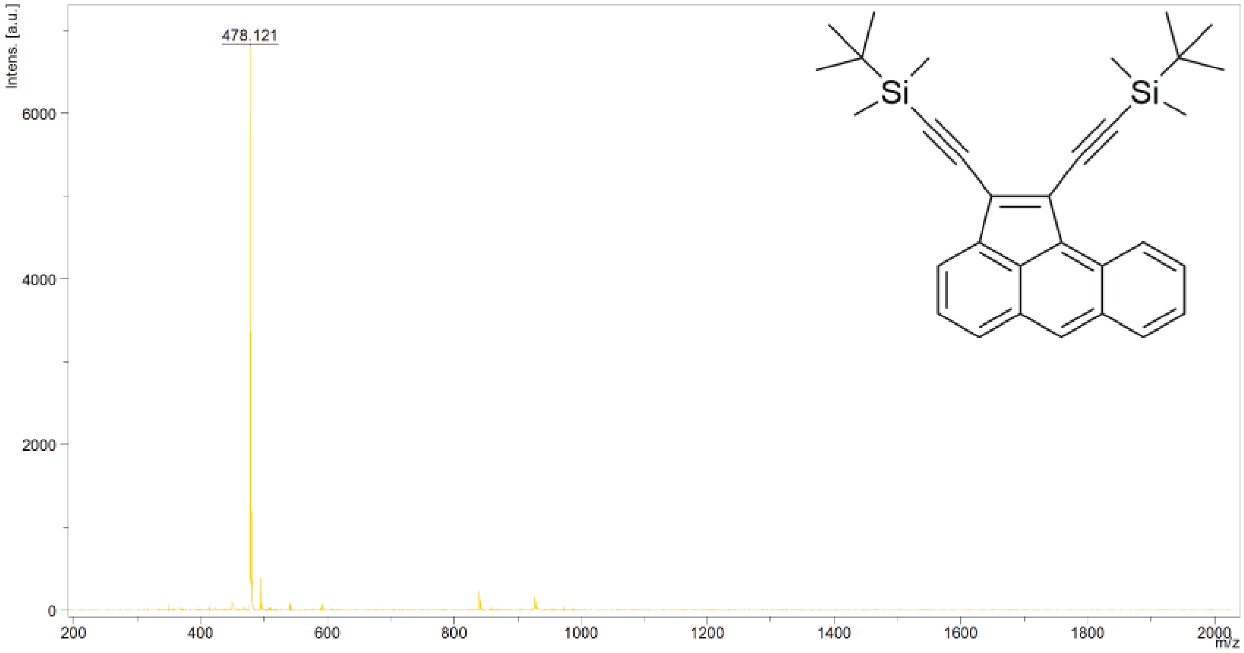

m/z S/N Quality Fac. Res. Intens. Area
478.121 6816

Figure S37. MALDI-MS spectrum of **TBDMS-aceAN**.

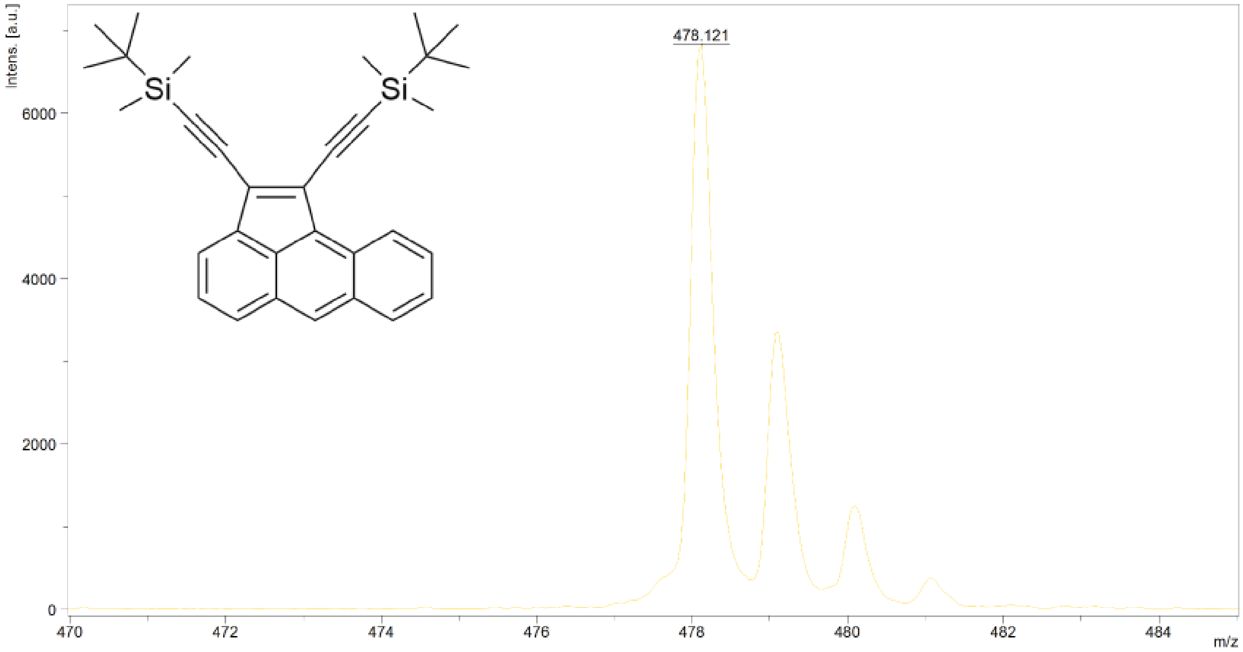

m/z S/N Quality Fac. Res. Intens. Area
478.121 6816

Figure S38. MALDI-MS spectrum of **TBDMS-aceAN**.

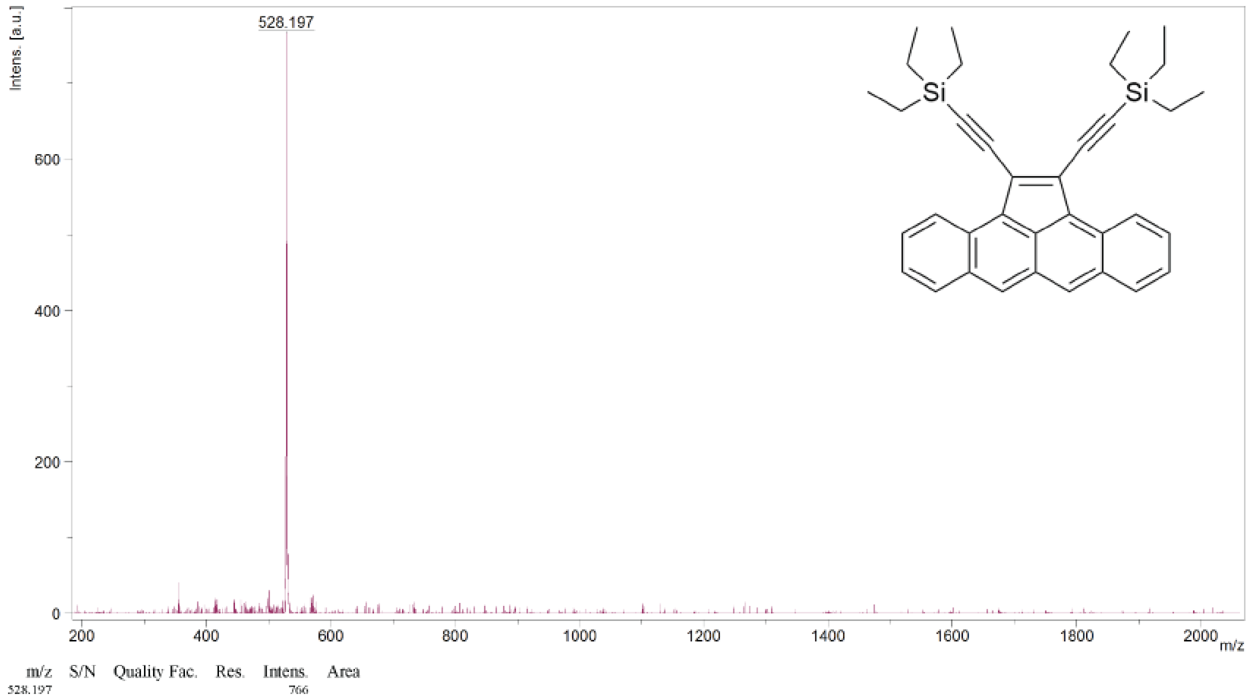

Figure S39. MALDI-MS spectrum of **TES-aceTN**.

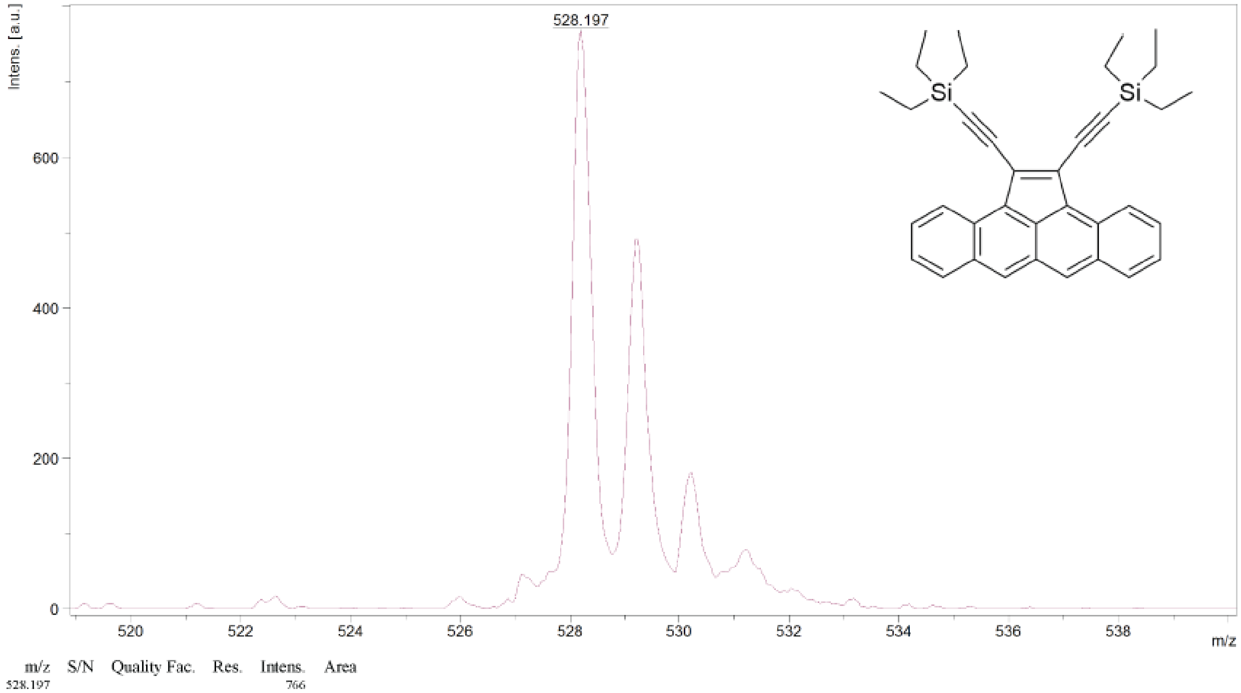

Figure S40. MALDI-MS spectrum of **TES-aceTN**.

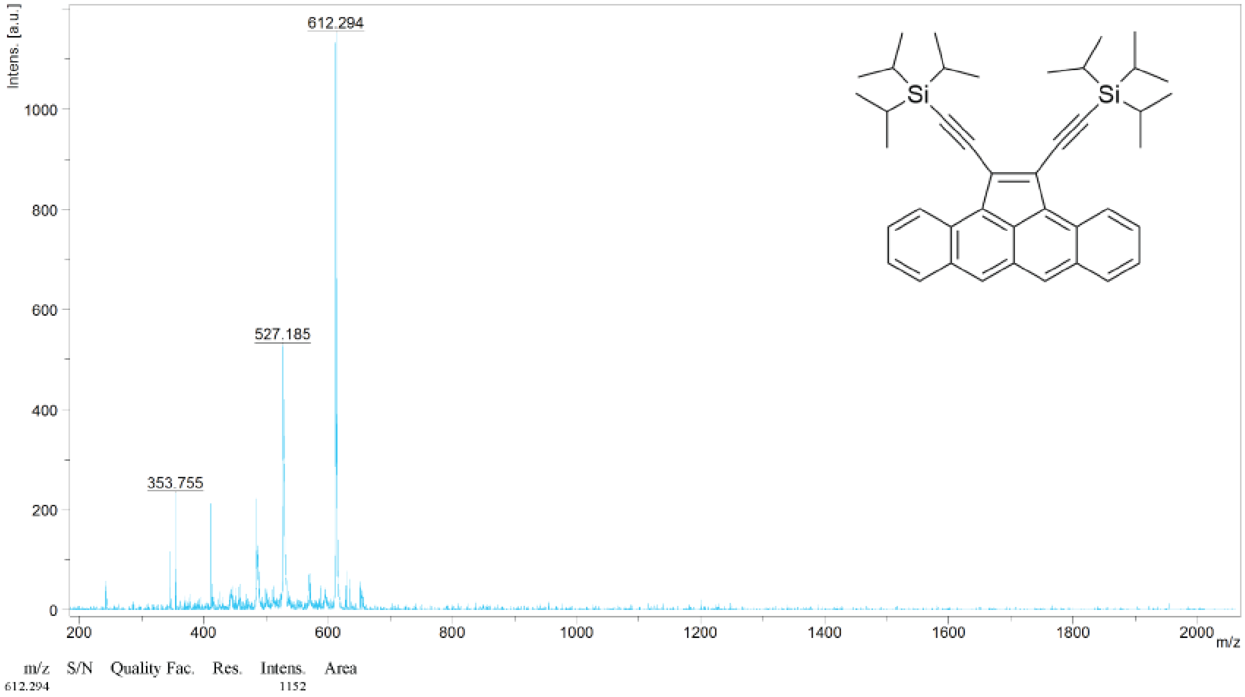

Figure S41. MALDI-MS spectrum of **TIPS-aceTN**. Lower mass peaks correspond to fragmentation of TIPS group.

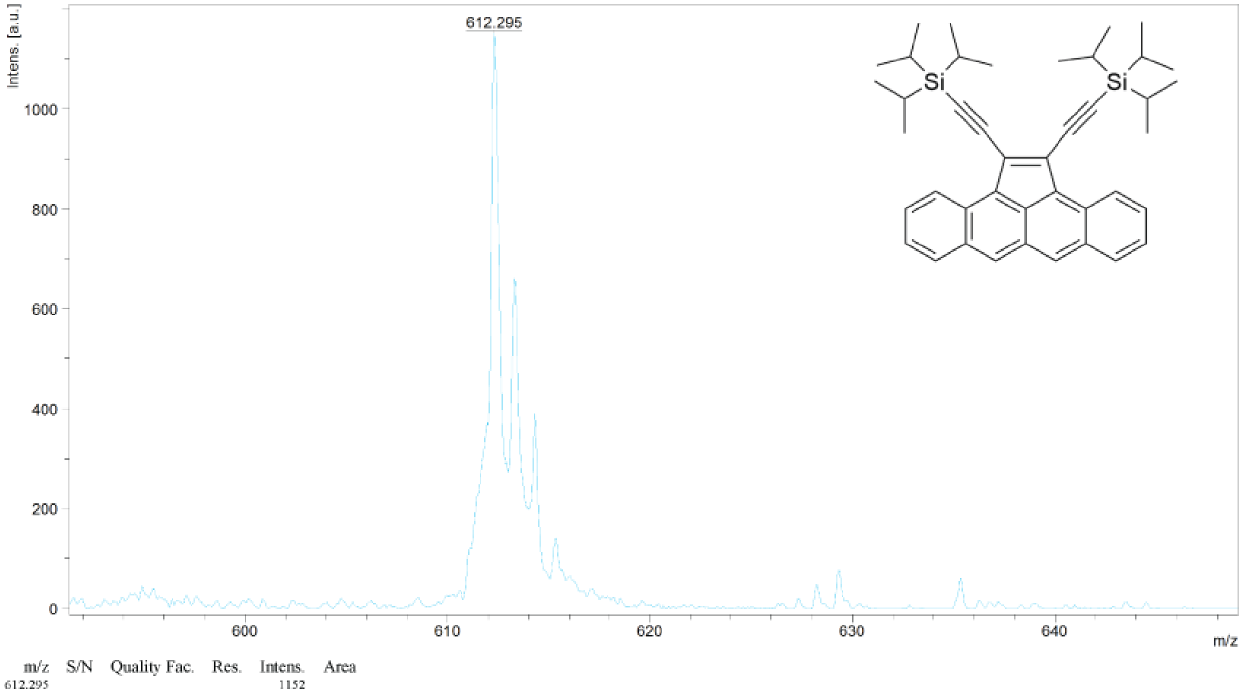

Figure S42. MALDI-MS spectrum of **TIPS-aceTN**.

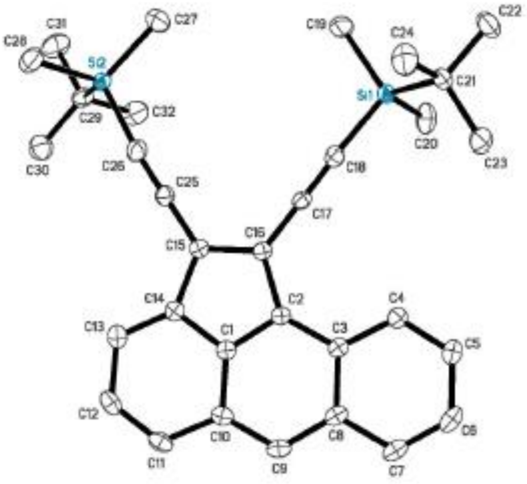

Figure S43 and Table S6. Ellipsoid plot (50%) of **TBDMS-aceAN**. Crystal data and structure refinement for m23163.

Identification code	m23163	
Empirical formula	C32 H38 Si2	
Formula weight	478.80	
Temperature	100.0(2) K	
Wavelength	0.71073 A	
Crystal system, space group	Triclinic, P-1	
Unit cell dimensions	a = 7.3564(3) A	alpha = 96.151(2) deg.
	b = 11.2360(6) A	beta = 101.525(2) deg.
	c = 18.2803(10) A	gamma = 101.285(2) deg.
Volume	1434.98(13) A^3	
Z, Calculated density	2, 1.108 Mg/m^3	
Absorption coefficient	0.141 mm^-1	
F(000)	516	
Crystal size	0.240 x 0.130 x 0.110 mm	
Theta range for data collection	2.041 to 27.514 deg.	
Limiting indices	-8<=h<=9, -14<=k<=14, -23<=l<=23	
Reflections collected / unique	45981 / 6603 [R(int) = 0.0379]	
Completeness to theta = 25.242	99.9 %	
Absorption correction	Semi-empirical from equivalents	
Max. and min. transmission	0.959 and 0.901	
Refinement method	Full-matrix least-squares on F^2	
Data / restraints / parameters	6603 / 0 / 317	
Goodness-of-fit on F^2	1.054	
Final R indices [I>2sigma(I)]	R1 = 0.0352, wR2 = 0.0878	
R indices (all data)	R1 = 0.0431, wR2 = 0.0931	
Extinction coefficient	n/a	
Largest diff. peak and hole	0.409 and -0.241 e.A^-3	

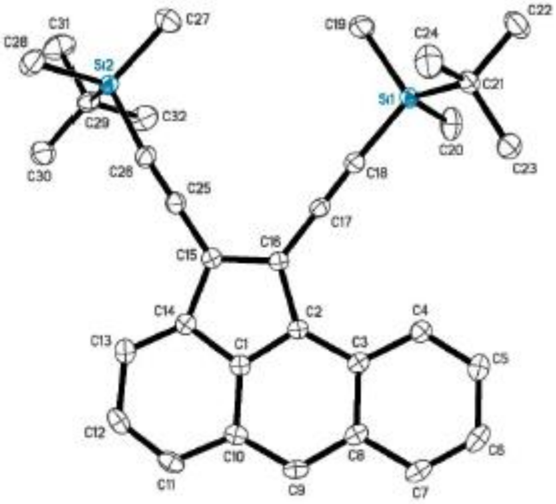

Figure S44 and Table S7. Ellipsoid plot (50%) of **TES-aceTN**. Crystal data and refinement for x13133.

Identification code	x13133
Empirical formula	C36 H40 Si2
Formula weight	528.86
Temperature	90.0(2) K
Wavelength	1.54178 A
Crystal system, space group	Orthorhombic, P b c a
Unit cell dimensions	a = 7.7326(3) A alpha = 90 deg.
	b = 23.4376(9) A beta = 90 deg.
	c = 33.5260(12) A gamma = 90 deg.
Volume	6076.0(4) A^3
Z, Calculated density	8, 1.156 Mg/m^3
Absorption coefficient	1.212 mm^-1
F(000)	2272
Crystal size	0.300 x 0.060 x 0.010 mm
Theta range for data collection	3.772 to 68.433 deg.
Limiting indices	-9<=h<=6, -27<=k<=27, -40<=l<=40
Reflections collected / unique	49530 / 5246 [R(int) = 0.0436]
Completeness to theta = 67.679	94.5 %
Absorption correction	Semi-empirical from equivalents
Max. and min. transmission	0.7531 and 0.5603
Refinement method	Full-matrix least-squares on F^2
Data / restraints / parameters	5246 / 0 / 349
Goodness-of-fit on F^2	1.135
Final R indices [I>2sigma(I)]	R1 = 0.0486, wR2 = 0.1380
R indices (all data)	R1 = 0.0531, wR2 = 0.1409
Extinction coefficient	n/a
Largest diff. peak and hole	0.322 and -0.376 e.A^-3

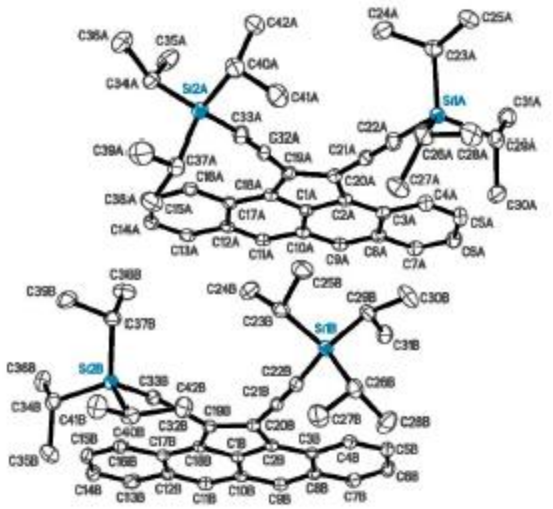

Figure S45 and Table S8. Ellipsoid plot (50%) of TIPS-aceTN. Crystal data and structure refinement for k13054c.

Identification code	k13054c
Empirical formula	C42 H52 Si2
Formula weight	613.01
Temperature	90.0(2) K
Wavelength	0.71073 A
Crystal system, space group	Triclinic, P -1
Unit cell dimensions	a = 13.7321(1) A alpha = 93.3568(5) deg.
	b = 13.9472(2) A beta = 106.5745(5) deg.
	c = 20.0284(2) A gamma = 96.7673(5) deg.
Volume	3633.77(7) A^3
Z, Calculated density	4, 1.121 Mg/m^3
Absorption coefficient	0.125 mm^-1
F(000)	1328
Crystal size	0.300 x 0.250 x 0.030 mm
Theta range for data collection	1.477 to 27.497 deg.
Limiting indices	-17<=h<=17, -18<=k<=18, -26<=l<=26
Reflections collected / unique	90797 / 16645 [R(int) = 0.0539]
Completeness to theta = 25.242	100.0 %
Absorption correction	Semi-empirical from equivalents
Max. and min. transmission	0.9878 and 0.8833
Refinement method	Full-matrix least-squares on F^2
Data / restraints / parameters	16645 / 0 / 817
Goodness-of-fit on F^2	1.030
Final R indices [I>2sigma(I)]	R1 = 0.0474, wR2 = 0.1101
R indices (all data)	R1 = 0.0806, wR2 = 0.1258
Extinction coefficient	n/a
Largest diff. peak and hole	0.337 and -0.328 e.A^-3

TDDFT Tables

Table S9: Spin orbit couplings (meV) between the first five singlet and triplet excited states of **TES-aceAN** optimized at TD-CAM-B3LYP-D3(BJ)/6-311++G**/PCM(chloroform) level of theory.

	T_1	T_2	T_3	T_4	T_5
S_0	0.0073	0.0050	0.0180	0.1681	0.0171
S_1	0.0032	0.0198	0.0391	0.0070	0.0053
S_2	0.0094	0.0192	0.0460	0.0344	0.0230
S_3	0.0826	0.0908	0.0187	0.0029	0.0254
S_4	0.4585	0.4632	0.2259	0.1970	0.2577
S_5	0.0189	0.0067	0.0198	0.0085	0.0099

Table S10: Spin orbit couplings (meV) between the first five singlet and triplet excited states of **TBDMS-aceAN** optimized at TD-CAM-B3LYP-D3(BJ)/6-311++G**/PCM(chloroform) level of theory.

	T_1	T_2	T_3	T_4	T_5
S_0	0.0043	0.0055	0.0173	0.1694	0.0139
S_1	0.0038	0.0205	0.0392	0.0068	0.0052
S_2	0.0089	0.0197	0.0468	0.0342	0.0238
S_3	0.0817	0.0918	0.0186	0.0028	0.0255
S_4	0.4171	0.4807	0.1910	0.1709	0.2828
S_5	0.0303	0.0460	0.0166	0.0091	0.0351

Table S11: Spin orbit couplings (meV) between the first five singlet and triplet excited states of **TES-aceTN** optimized at TD-CAM-B3LYP-D3(BJ)/6-311++G**/PCM(toluene) level of theory.

	T_1	T_2	T_3	T_4	T_5
S_0	0.0018	0.0210	0.0673	0.1517	0.0040
S_1	0.0229	0.0011	0.0007	0.0003	0.0254
S_2	0.0004	0.0265	0.0394	0.0607	0.0009
S_3	0.1211	0.0007	0.0007	0.0003	0.0297
S_4	0.0114	0.0034	0.0010	0.0007	0.0238
S_5	0.2807	0.5069	0.3031	0.1427	0.2459

Table S12: Spin orbit couplings in (meV) between the first five singlet and triplet excited states of **TIPS-aceTN** optimized at TD-CAM-B3LYP-D3(BJ)/6-311++G**/PCM(toluene) level of theory.

	T_1	T_2	T_3	T_4	T_5
S_0	0.0010	0.0051	0.0732	0.1742	0.0030
S_1	0.0240	0.0011	0.0010	0.0013	0.0243
S_2	0.0004	0.0232	0.0393	0.0583	0.0006
S_3	0.1201	0.0019	0.0006	0.0002	0.0299
S_4	0.0203	0.0043	0.0019	0.0008	0.0230
S_5	0.2802	0.4807	0.2883	0.1338	0.2516

Table S13: Spin orbit couplings (meV) between the first five singlet and triplet excited states of monomer-1 (m1) extracted from the **TBDMS-aceAN** crystal at TD-CAM-B3LYP-D3(BJ)/6-311++G**level of theory.

	T_1	T_2	T_3	T_4	T_5
S_0	0.0080	0.0129	0.0069	0.1583	0.0221
S_1	0.0049	0.0184	0.0342	0.0064	0.0092
S_2	0.0075	0.0206	0.0514	0.0337	0.0261
S_3	0.0796	0.0982	0.0194	0.0048	0.0241
S_4	0.4767	0.4330	0.2156	0.2216	0.2405
S_5	0.4314	0.3273	0.1964	0.2321	0.3501

Table S14: Spin orbit couplings (meV) between the first five singlet and triplet excited states of monomer-1 (m1) extracted from the **TES-aceTN** crystal at TD-CAM-B3LYP-D3(BJ)/6-311++G** level of theory.

	T_1	T_2	T_3	T_4	T_5
S_0	0.0011	0.0252	0.0575	0.1509	0.0042
S_1	0.0219	0.0016	0.0037	0.0037	0.0229
S_2	0.0012	0.0244	0.0394	0.0613	0.0056
S_3	0.1255	0.0043	0.0007	0.0005	0.0307
S_4	0.0211	0.0151	0.0066	0.0032	0.0225
S_5	0.2820	0.5191	0.3042	0.1632	0.2327

Table S15: Spin orbit couplings (meV) between the first five singlet and triplet excited states of monomer-1 (m1) extracted from the **TIPS-aceTN** crystal at TD-CAM-B3LYP-D3(BJ)/6-311++G** level of theory.

	T_1	T_2	T_3	T_4	T_5
S_0	0.0085	0.0340	0.0713	0.1728	0.0143
S_1	0.0230	0.0042	0.0043	0.0031	0.0239
S_2	0.0033	0.0229	0.0398	0.0598	0.0068
S_3	0.1240	0.0082	0.0023	0.0009	0.0328
S_4	0.0332	0.0156	0.0092	0.0029	0.0197
S_5	0.2886	0.4907	0.2842	0.1497	0.2554

Table S16: Spin orbit couplings (meV) between the first ten singlet and triplet excited states of dimer-12 (d12) extracted from the **TBDMS-aceAN** crystal at TD-CAM-B3LYP-D3(BJ)/6-311++G** level of theory.

	**T_1**	**T_2**	**T_3**	**T_4**	**T_5**	**T_6**	**T_7**	**T_8**	**T_9**	**T_{10}**
**S_0**	0.1310	0.0000	0.0001	0.1348	0.0000	0.1372	0.1693	0.0000	0.0000	0.1988
**S_1**	0.0133	0.0000	0.0000	0.0226	0.0000	0.0480	0.0234	0.0000	0.0000	0.0187
**S_2**	0.0000	0.0167	0.0199	0.0000	0.0382	0.0000	0.0000	0.0216	0.0198	0.0000
**S_3**	0.0000	0.0161	0.0257	0.0000	0.0428	0.0000	0.0000	0.0069	0.0392	0.0000
**S_4**	0.0282	0.0000	0.0000	0.0248	0.0000	0.0610	0.0103	0.0000	0.0000	0.0519
**S_5**	0.0000	0.0196	0.0206	0.0000	0.0394	0.0000	0.0000	0.0048	0.0347	0.0000
**S_6**	0.0205	0.0000	0.0000	0.0285	0.0000	0.0108	0.0032	0.0000	0.0000	0.0048
**S_7**	0.0559	0.0000	0.0000	0.0567	0.0000	0.0106	0.0105	0.0000	0.0000	0.0243
**S_8**	0.0000	0.0343	0.0125	0.0000	0.0162	0.0000	0.0000	0.0306	0.0471	0.0000
**S_9**	0.0752	0.0000	0.0000	0.1006	0.0000	0.0119	0.0038	0.0000	0.0000	0.0156
**S_{10}**	0.0000	0.1135	0.1205	0.0000	0.0227	0.0000	0.0000	0.0102	0.0120	0.0000

Table S17: Spin orbit couplings (meV) between the first ten singlet and triplet excited states of dimer-23 (d23) extracted from the **TBDMS-aceAN** crystal at TD-CAM-B3LYP-D3(BJ)/6-311++G** level of theory.

	**T_1**	**T_2**	**T_3**	**T_4**	**T_5**	**T_6**	**T_7**	**T_8**	**T_9**	**T_{10}**
**S_0**	0.0000	0.1123	0.0000	0.1482	0.0842	0.0000	0.2634	0.0001	0.0000	0.0906
**S_1**	0.0000	0.0188	0.0000	0.0234	0.0454	0.0000	0.0214	0.0000	0.0000	0.0340
**S_2**	0.0209	0.0000	0.0224	0.0000	0.0000	0.0414	0.0000	0.0150	0.0368	0.0000
**S_3**	0.0000	0.0217	0.0000	0.0146	0.0376	0.0000	0.0376	0.0000	0.0000	0.0194
**S_4**	0.0161	0.0000	0.0100	0.0000	0.0000	0.0255	0.0000	0.0300	0.0216	0.0000
**S_5**	0.0000	0.0204	0.0000	0.0413	0.0318	0.0000	0.0361	0.0000	0.0000	0.0166
**S_6**	0.0269	0.0000	0.0371	0.0000	0.0000	0.0514	0.0000	0.0408	0.0397	0.0000
**S_7**	0.0000	0.0842	0.0000	0.0884	0.0226	0.0000	0.0048	0.0000	0.0000	0.0170
**S_8**	0.0732	0.0000	0.0763	0.0000	0.0000	0.0142	0.0000	0.0066	0.0190	0.0000
**S_9**	0.0000	0.0647	0.0000	0.0767	0.0263	0.0000	0.0061	0.0000	0.0000	0.0069
**S_{10}**	0.0932	0.0000	0.0947	0.0000	0.0000	0.0371	0.0000	0.0101	0.0194	0.0000

Table S18: Spin orbit couplings (meV) between the first ten singlet and triplet excited states of dimer-12 (d12) extracted from the **TES-aceTN** crystal at TD-CAM-B3LYP-D3(BJ)/6-311++G** level of theory.

	T_1	T_2	T_3	T_4	T_5	T_6	T_7	T_8	T_9	T_{10}
S_0	0.0747	0.0931	0.0126	0.1610	0.0774	0.0232	0.2445	0.0875	0.0464	0.0455
S_1	0.0172	0.0149	0.0220	0.0033	0.0042	0.0232	0.0029	0.0069	0.0029	0.0128
S_2	0.0043	0.0046	0.0023	0.0344	0.0522	0.0159	0.0321	0.0110	0.0231	0.0152
S_3	0.0200	0.0237	0.0014	0.0262	0.0301	0.0099	0.0182	0.0063	0.0183	0.0078
S_4	0.0146	0.0119	0.0403	0.0030	0.0113	0.0440	0.0091	0.0123	0.0024	0.0229
S_5	0.0169	0.0208	0.0044	0.0116	0.0196	0.0076	0.0116	0.0051	0.0159	0.0063
S_6	0.0159	0.0144	0.0224	0.0024	0.0096	0.0259	0.0088	0.0151	0.0024	0.0245
S_7	0.0140	0.0133	0.0043	0.0145	0.0042	0.0102	0.0112	0.0114	0.0211	0.0147
S_8	0.0101	0.0232	0.0012	0.0150	0.0035	0.0063	0.0079	0.0146	0.0270	0.0050
S_9	0.0866	0.0966	0.0048	0.0102	0.0096	0.0044	0.0157	0.0064	0.0139	0.0030
S_{10}	0.1234	0.1033	0.0293	0.0057	0.0078	0.0221	0.0068	0.0150	0.0023	0.0226

Table S19: Spin orbit couplings (meV) between the first ten singlet and triplet excited states of dimer-12 (d12) extracted from the **TIPS-aceTN** crystal at TD-CAM-B3LYP-D3(BJ)/6-311++G** level of theory.

	T_1	T_2	T_3	T_4	T_5	T_6	T_7	T_8	T_9	T_{10}
S_0	0.0001	0.1106	0.0001	0.1618	0.0001	0.0739	0.2322	0.0001	0.0704	0.0001
S_1	0.0212	0.0000	0.0223	0.0000	0.0171	0.0000	0.0000	0.0053	0.0000	0.0179
S_2	0.0000	0.0194	0.0000	0.0207	0.0000	0.0204	0.0109	0.0000	0.0196	0.0000
S_3	0.0000	0.0094	0.0000	0.0441	0.0000	0.0529	0.0146	0.0000	0.0287	0.0000
S_4	0.0180	0.0000	0.0433	0.0000	0.0377	0.0000	0.0000	0.0153	0.0000	0.0550
S_5	0.0000	0.0417	0.0000	0.0087	0.0000	0.0071	0.0162	0.0000	0.0229	0.0000
S_6	0.0188	0.0000	0.0251	0.0000	0.0236	0.0000	0.0000	0.0276	0.0000	0.0204
S_7	0.0000	0.0181	0.0000	0.0148	0.0000	0.0145	0.0018	0.0000	0.0213	0.0000
S_8	0.0204	0.0000	0.0033	0.0000	0.0226	0.0000	0.0000	0.0154	0.0000	0.0053
S_9	0.0000	0.1424	0.0000	0.0190	0.0000	0.0184	0.0142	0.0000	0.0126	0.0000
S_{10}	0.1634	0.0000	0.0255	0.0000	0.0285	0.0000	0.0000	0.0198	0.0000	0.0108

Table S20: Spin orbit couplings (meV) between the first ten singlet and triplet excited states of dimer-23 (d23) extracted from the **TIPS-aceTN** crystal at TD-CAM-B3LYP-D3(BJ)/6-311++G** level of theory.

	T_1	T_2	T_3	T_4	T_5	T_6	T_7	T_8	T_9	T_{10}
S_0	0.1058	0.0687	0.0260	0.1497	0.0584	0.0551	0.2269	0.1393	0.0975	0.1031
S_1	0.0114	0.0206	0.0187	0.0207	0.0229	0.0272	0.0210	0.0041	0.0041	0.0231
S_2	0.0125	0.0086	0.0124	0.0303	0.0386	0.0175	0.0429	0.0246	0.0245	0.0203
S_3	0.0240	0.0153	0.0024	0.0198	0.0155	0.0150	0.0145	0.0099	0.0075	0.0126
S_4	0.0086	0.0123	0.0407	0.0082	0.0152	0.0405	0.0220	0.0116	0.0408	0.0240
S_5	0.0188	0.0113	0.0158	0.0164	0.0086	0.0248	0.0121	0.0111	0.0117	0.0329
S_6	0.0112	0.0235	0.0131	0.0070	0.0167	0.0073	0.0054	0.0187	0.0186	0.0140
S_7	0.0140	0.0189	0.0070	0.0161	0.0122	0.0113	0.0239	0.0050	0.0239	0.0056
S_8	0.0088	0.0247	0.0066	0.0125	0.0085	0.0098	0.0048	0.0193	0.0100	0.0074
S_9	0.1176	0.0610	0.0081	0.0099	0.0079	0.0046	0.0076	0.0088	0.0096	0.0087
S_{10}	0.0899	0.1320	0.0165	0.0071	0.0119	0.0175	0.0074	0.0142	0.0022	0.0085

Table S21: Spin orbit couplings (meV) between the first ten singlet and triplet excited states of dimer-34 (d34) extracted from the **TIPS-aceTN** crystal at TD-CAM-B3LYP-D3(BJ)/6-311++G** level of theory.

	T_1	T_2	T_3	T_4	T_5	T_6	T_7	T_8	T_9	T_{10}
S_0	0.0001	0.0509	0.0698	0.0001	0.1133	0.0001	0.0001	0.1345	0.0320	0.0001
S_1	0.0000	0.0158	0.0027	0.0000	0.0048	0.0000	0.0000	0.0024	0.0085	0.0000
S_2	0.0155	0.0000	0.0000	0.0048	0.0000	0.0055	0.0052	0.0000	0.0000	0.0108
S_3	0.0011	0.0000	0.0000	0.0304	0.0000	0.0325	0.0594	0.0000	0.0000	0.0099
S_4	0.0000	0.0018	0.0307	0.0000	0.0327	0.0000	0.0000	0.0601	0.0206	0.0000
S_5	0.0000	0.0073	0.0046	0.0000	0.0036	0.0000	0.0000	0.0085	0.0022	0.0000
S_6	0.0230	0.0000	0.0000	0.0018	0.0000	0.0037	0.0100	0.0000	0.0000	0.0061
S_7	0.0000	0.0495	0.0037	0.0000	0.0058	0.0000	0.0000	0.0059	0.0243	0.0000
S_8	0.0222	0.0000	0.0000	0.0050	0.0000	0.0065	0.0048	0.0000	0.0000	0.0198
S_9	0.0000	0.1025	0.0038	0.0000	0.0034	0.0000	0.0000	0.0031	0.0097	0.0000
S_{10}	0.1118	0.0000	0.0000	0.0048	0.0000	0.0056	0.0052	0.0000	0.0000	0.0176

Table S22. Singlet excited states of **TES-aceAN** optimized in a chloroform PCM with singlet spin.

Excited State	Energy (eV)	λ (nm)	Oscillator Strength	<S**2>
1	2.5535	485.55	0.1738	0
2	3.1061	399.16	0.2948	0
3	3.7847	327.59	0.2532	0
4	4.3628	284.18	0.0004	0
5	4.4398	279.25	0.0658	0
6	4.4809	276.69	0.0001	0
7	4.7105	263.21	0.2546	0
8	4.7788	259.44	0.6997	0
9	4.8947	253.3	0.1951	0
10	5.2715	235.2	0.3615	0
11	5.3307	232.58	0.2784	0
12	5.3416	232.11	0.0036	0
13	5.4314	228.27	0.0011	0
14	5.4395	227.93	0.2254	0
15	5.4774	226.36	0.0014	0
16	5.6767	218.41	0.1808	0
17	5.6905	217.88	0.0059	0
18	5.7519	215.55	0.3575	0
19	5.8578	211.66	0.0038	0
20	5.8768	210.97	0.089	0
21	5.8943	210.35	0.0069	0
22	5.9314	209.03	0.0001	0
23	5.9441	208.58	0.0019	0
24	5.9916	206.93	0.0035	0
25	6.0035	206.52	0.0007	0
26	6.0228	205.86	0.0001	0
27	6.061	204.56	0.0547	0
28	6.1003	203.24	0.1254	0
29	6.1361	202.06	0.0008	0
30	6.152	201.54	0.0476	0

Table S23. Singlet excited states of **TES-aceAN** optimized in a toluene PCM with singlet spin.

Excited State	Energy (eV)	λ (nm)	Oscillator Strength	<S**2>
1	2.543	487.55	0.1738	0
2	3.1057	399.22	0.3012	0
3	3.7827	327.77	0.2615	0
4	4.3519	284.89	0.0003	0
5	4.4353	279.54	0.067	0
6	4.4731	277.18	0.0002	0
7	4.7083	263.33	0.2954	0
8	4.7666	260.11	0.695	0
9	4.8907	253.51	0.192	0
10	5.2645	235.51	0.4112	0
11	5.3225	232.94	0.2691	0
12	5.3332	232.48	0.0037	0
13	5.4219	228.67	0.0007	0
14	5.4367	228.05	0.219	0
15	5.4631	226.95	0.0022	0
16	5.6635	218.92	0.0076	0
17	5.671	218.63	0.1762	0
18	5.7484	215.68	0.3405	0
19	5.8379	212.38	0.0041	0
20	5.8659	211.37	0.0963	0
21	5.8885	210.55	0.0045	0
22	5.9371	208.83	0.0005	0
23	5.9648	207.86	0.0013	0
24	5.9875	207.07	0.0001	0
25	6.0086	206.35	0.0041	0
26	6.0195	205.97	0.002	0
27	6.0698	204.26	0.0395	0
28	6.0874	203.67	0.0012	0
29	6.0989	203.29	0.1264	0
30	6.1271	202.35	0.0436	0

Table S24. Triplet excited states of **TES-aceAN** optimized in a chloroform PCM with singlet spin.

Excited State	Energy (eV)	λ (nm)	Oscillator Strength	<S**2>
1	1.2741	973.1	0	2
2	1.694	731.89	0	2
3	3.0162	411.06	0	2
4	3.1351	395.48	0	2
5	3.7623	329.55	0	2
6	3.9464	314.17	0	2
7	4.1649	297.69	0	2
8	4.1976	295.37	0	2
9	4.2687	290.45	0	2
10	4.326	286.6	0	2
11	4.5447	272.81	0	2
12	4.5969	269.71	0	2
13	4.7535	260.83	0	2
14	4.9225	251.87	0	2
15	5.0021	247.86	0	2
16	5.073	244.4	0	2
17	5.0822	243.96	0	2
18	5.1498	240.75	0	2
19	5.2686	235.32	0	2
20	5.2957	234.12	0	2
21	5.3914	229.97	0	2
22	5.398	229.69	0	2
23	5.5811	222.15	0	2
24	5.6671	218.78	0	2
25	5.6825	218.18	0	2
26	5.7409	215.97	0	2
27	5.788	214.21	0	2
28	5.8173	213.13	0	2
29	5.8452	212.11	0	2
30	5.8814	210.81	0	2

Table S25. Triplet excited states of **TES-aceAN** optimized in a toluene PCM with singlet spin.

Excited State	Energy (eV)	λ (nm)	Oscillator Strength	<S**2>
1	1.2652	979.96	0	2
2	1.6877	734.65	0	2
3	3.0204	410.49	0	2
4	3.134	395.61	0	2
5	3.758	329.92	0	2
6	3.9433	314.42	0	2
7	4.154	298.47	0	2
8	4.1937	295.64	0	2
9	4.2605	291.01	0	2
10	4.3241	286.73	0	2
11	4.5441	272.85	0	2
12	4.5867	270.31	0	2
13	4.7456	261.26	0	2
14	4.9179	252.11	0	2
15	4.9956	248.19	0	2
16	5.0635	244.86	0	2
17	5.073	244.4	0	2
18	5.1369	241.36	0	2
19	5.2626	235.59	0	2
20	5.2945	234.17	0	2
21	5.3866	230.17	0	2
22	5.3927	229.91	0	2
23	5.5866	221.93	0	2
24	5.6415	219.77	0	2
25	5.6855	218.07	0	2
26	5.7424	215.91	0	2
27	5.7975	213.86	0	2
28	5.8273	212.76	0	2
29	5.843	212.19	0	2
30	5.8607	211.55	0	2

Table S26. Singlet excited states of **TBDMS-aceAN** optimized in a chloroform PCM with singlet spin.

Excited State	Energy (eV)	λ (nm)	Oscillator Strength	<S**2>
1	2.5517	485.9	0.1747	0
2	3.1047	399.35	0.2957	0
3	3.7832	327.72	0.2544	0
4	4.3806	283.03	0.0014	0
5	4.4324	279.73	0.071	0
6	4.4801	276.74	0.0003	0
7	4.7039	263.58	0.2198	0
8	4.7759	259.6	0.7296	0
9	4.8897	253.56	0.1787	0
10	5.2241	237.33	0.1518	0
11	5.318	233.14	0.3821	0
12	5.3451	231.96	0.0004	0
13	5.3692	230.92	0.2507	0
14	5.4173	228.87	0.0029	0
15	5.495	225.63	0.0015	0
16	5.6195	220.63	0.2524	0
17	5.6752	218.47	0.0179	0
18	5.6965	217.65	0.157	0
19	5.7628	215.14	0.1272	0
20	5.8763	210.99	0.1456	0
21	5.8846	210.69	0.0479	0
22	5.9104	209.77	0.0292	0
23	5.9353	208.89	0.0006	0
24	5.9902	206.98	0.0012	0
25	6.0167	206.07	0.0007	0
26	6.0982	203.31	0.1063	0
27	6.1147	202.76	0.0047	0
28	6.1718	200.89	0.0385	0
29	6.1834	200.51	0.1023	0
30	6.1984	200.03	0.119	0

Table S27. Singlet excited states of **TBDMS-aceAN** optimized in a toluene PCM with singlet spin.

Excited State	Energy (eV)	λ (nm)	Oscillator Strength	<S**2>
1	2.5411	487.92	0.1742	0
2	3.105	399.31	0.302	0
3	3.7814	327.88	0.2623	0
4	4.367	283.91	0.0011	0
5	4.4275	280.04	0.0714	0
6	4.4662	277.61	0.0004	0
7	4.702	263.69	0.249	0
8	4.7633	260.29	0.7345	0
9	4.8872	253.69	0.1763	0
10	5.2294	237.09	0.1857	0
11	5.3098	233.5	0.4369	0
12	5.3383	232.25	0.0018	0
13	5.3754	230.65	0.2011	0
14	5.4104	229.16	0.0024	0
15	5.4817	226.18	0.0015	0
16	5.6096	221.02	0.2025	0
17	5.6518	219.37	0.0633	0
18	5.7072	217.24	0.1894	0
19	5.761	215.21	0.0795	0
20	5.8389	212.34	0.0292	0
21	5.876	211	0.1262	0
22	5.8851	210.68	0.0582	0
23	5.9383	208.79	0.0006	0
24	5.9841	207.19	0.0006	0
25	5.9966	206.76	0.001	0
26	6.0764	204.04	0.005	0
27	6.0897	203.6	0.0957	0
28	6.1257	202.4	0.0301	0
29	6.1725	200.86	0.0618	0
30	6.1939	200.17	0.1669	0

Table S28. Triplet excited states of **TBDMS-aceAN** optimized in a chloroform PCM with singlet spin.

Excited State	Energy (eV)	λ (nm)	Oscillator Strength	<S**2>
1	1.2733	973.72	0	2
2	1.6927	732.47	0	2
3	3.014	411.36	0	2
4	3.1329	395.75	0	2
5	3.7599	329.75	0	2
6	3.9471	314.11	0	2
7	4.1774	296.8	0	2
8	4.1937	295.64	0	2
9	4.2639	290.78	0	2
10	4.3216	286.9	0	2
11	4.5415	273	0	2
12	4.5956	269.79	0	2
13	4.7538	260.81	0	2
14	4.9201	251.99	0	2
15	4.9996	247.99	0	2
16	5.0702	244.53	0	2
17	5.0793	244.1	0	2
18	5.1639	240.1	0	2
19	5.2473	236.28	0	2
20	5.2942	234.19	0	2
21	5.3225	232.94	0	2
22	5.3781	230.53	0	2
23	5.5471	223.51	0	2
24	5.5938	221.65	0	2
25	5.6261	220.37	0	2
26	5.6492	219.47	0	2
27	5.7086	217.19	0	2
28	5.7446	215.83	0	2
29	5.7965	213.89	0	2
30	5.8826	210.76	0	2

Table S29. Triplet excited states of **TBDMS-aceAN** optimized in a toluene PCM with singlet spin.

Excited State	Energy (eV)	λ (nm)	Oscillator Strength	<S**2>
1	1.2639	980.97	0	2
2	1.6869	734.98	0	2
3	3.0181	410.8	0	2
4	3.1318	395.88	0	2
5	3.756	330.09	0	2
6	3.944	314.36	0	2
7	4.1645	297.72	0	2
8	4.1892	295.96	0	2
9	4.2506	291.68	0	2
10	4.3196	287.03	0	2
11	4.541	273.03	0	2
12	4.5859	270.36	0	2
13	4.7461	261.23	0	2
14	4.9156	252.23	0	2
15	4.9934	248.3	0	2
16	5.0627	244.9	0	2
17	5.0701	244.54	0	2
18	5.1513	240.69	0	2
19	5.2465	236.32	0	2
20	5.2928	234.25	0	2
21	5.327	232.75	0	2
22	5.3748	230.68	0	2
23	5.5633	222.86	0	2
24	5.6057	221.18	0	2
25	5.6187	220.66	0	2
26	5.6346	220.04	0	2
27	5.709	217.17	0	2
28	5.7481	215.7	0	2
29	5.8022	213.69	0	2
30	5.832	212.59	0	2

Table S30. Singlet excited states of **TES-aceTN** optimized in a toluene PCM with singlet spin.

Excited State	Energy (eV)	λ (nm)	Oscillator Strength	<S**2>
1	2.2291	556.2	0.0949	0
2	2.3778	521.43	0.2951	0
3	3.4394	360.48	0.4014	0
4	3.9634	312.82	0.0018	0
5	4.2064	294.75	0.0003	0
6	4.328	286.47	0.0001	0
7	4.3688	283.79	0.0575	0
8	4.4336	279.65	0.3887	0
9	4.6451	266.91	0.0187	0
10	4.6531	266.46	1.8082	0
11	4.7877	258.96	0.3447	0
12	4.8398	256.18	0.107	0
13	5.0124	247.35	0.3843	0
14	5.2102	237.96	0.0304	0
15	5.2484	236.23	0.0006	0
16	5.2748	235.05	0	0
17	5.2858	234.56	0.0342	0
18	5.3747	230.68	0.0007	0
19	5.4143	228.99	0.001	0
20	5.4695	226.68	0.0011	0
21	5.6112	220.96	0.001	0
22	5.6615	219	0.0021	0
23	5.7187	216.8	0.0009	0
24	5.7284	216.44	0.0001	0
25	5.7373	216.1	0.0182	0
26	5.7476	215.71	0.0036	0
27	5.7538	215.48	0.0022	0
28	5.7697	214.89	0.0026	0
29	5.805	213.58	0.0549	0
30	5.8204	213.02	0.0001	0

Table S31. Triplet excited states of **TES-aceTN** optimized in a toluene PCM with singlet spin.

Excited State	Energy (eV)	λ (nm)	Oscillator Strength	<S**2>
1	0.8853	1400.54	0	2
2	1.1744	1055.71	0	2
3	2.5939	477.98	0	2
4	2.8339	437.5	0	2
5	3.2633	379.94	0	2
6	3.6186	342.63	0	2
7	3.8249	324.15	0	2
8	3.8428	322.64	0	2
9	4.0516	306.01	0	2
10	4.1628	297.84	0	2
11	4.187	296.11	0	2
12	4.2561	291.31	0	2
13	4.3226	286.83	0	2
14	4.4564	278.21	0	2
15	4.4616	277.89	0	2
16	4.706	263.46	0	2
17	4.9001	253.02	0	2
18	4.9091	252.56	0	2
19	4.9629	249.82	0	2
20	5.0181	247.07	0	2
21	5.0272	246.63	0	2
22	5.079	244.11	0	2
23	5.178	239.44	0	2
24	5.2339	236.89	0	2
25	5.2636	235.55	0	2
26	5.3797	230.47	0	2
27	5.4087	229.23	0	2
28	5.4778	226.34	0	2
29	5.5052	225.21	0	2
30	5.5369	223.93	0	2

Table S32. Singlet excited states of **TIPS-aceTN** optimized in a toluene PCM with singlet spin.

Excited State	Energy (eV)	λ (nm)	Oscillator Strength	<S**2>
1	2.2258	557.03	0.0932	0
2	2.3765	521.7	0.3045	0
3	3.4397	360.45	0.3974	0
4	3.9537	313.59	0.0015	0
5	4.1881	296.04	0.0003	0
6	4.2798	289.69	0.0001	0
7	4.3641	284.1	0.0599	0
8	4.4263	280.11	0.3903	0
9	4.6355	267.46	0.0347	0
10	4.6476	266.77	1.6921	0
11	4.7837	259.18	0.376	0
12	4.8095	257.79	0.1543	0
13	4.988	248.57	0.2785	0
14	5.0921	243.48	0.049	0
15	5.1811	239.3	0.1124	0
16	5.246	236.34	0.0004	0
17	5.2727	235.14	0.0001	0
18	5.3344	232.42	0.0015	0
19	5.3838	230.29	0.0006	0
20	5.3972	229.72	0.0014	0
21	5.4029	229.48	0.0016	0
22	5.4199	228.76	0.0004	0
23	5.5396	223.81	0.006	0
24	5.6127	220.9	0	0
25	5.7076	217.23	0.0035	0
26	5.7133	217.01	0.0036	0
27	5.7274	216.47	0.0002	0
28	5.7512	215.58	0.0001	0
29	5.7731	214.76	0.0043	0
30	5.7852	214.31	0.0157	0

Table S33. Triplet excited states of **TIPS-aceTN** optimized in a toluene PCM with singlet spin.

Excited State	Energy (eV)	λ (nm)	Oscillator Strength	<S**2>
1	0.884	1402.54	0	2
2	1.1724	1057.49	0	2
3	2.5919	478.36	0	2
4	2.8331	437.63	0	2
5	3.2616	380.13	0	2
6	3.6157	342.91	0	2
7	3.8248	324.16	0	2
8	3.8416	322.74	0	2
9	4.0403	306.87	0	2
10	4.1276	300.38	0	2
11	4.1787	296.7	0	2
12	4.2448	292.08	0	2
13	4.321	286.93	0	2
14	4.4534	278.41	0	2
15	4.4606	277.96	0	2
16	4.688	264.47	0	2
17	4.89	253.55	0	2
18	4.9061	252.72	0	2
19	4.9495	250.5	0	2
20	5.0059	247.68	0	2
21	5.0348	246.25	0	2
22	5.0759	244.26	0	2
23	5.0987	243.17	0	2
24	5.1942	238.7	0	2
25	5.2596	235.73	0	2
26	5.3048	233.72	0	2
27	5.3252	232.83	0	2
28	5.3407	232.15	0	2
29	5.3805	230.43	0	2
30	5.4068	229.31	0	2

Table S34. Singlet excited states of **m1** extracted from the **TBDMS-aceAN** crystal with singlet spin.

Excited State	Energy (eV)	λ (nm)	Oscillator Strength	<S**2>
1	2.5021	495.52	0.1097	0
2	3.1219	397.14	0.2294	0
3	3.7804	327.97	0.1496	0
4	4.3221	286.86	0.0001	0
5	4.4336	279.65	0.0119	0
6	4.4584	278.09	0.0367	0
7	4.6834	264.73	0.0516	0
8	4.801	258.25	0.4792	0
9	4.9042	252.81	0.1914	0
10	5.1985	238.5	0.1425	0

Table S35. Singlet excited states of **m2** extracted from the **TBDMS-aceAN** crystal with singlet spin.

Excited State	Energy (eV)	λ (nm)	Oscillator Strength	<S**2>
1	2.5021	495.52	0.1097	0
2	3.1219	397.15	0.2294	0
3	3.7804	327.97	0.1496	0
4	4.3221	286.86	0.0001	0
5	4.4336	279.65	0.0119	0
6	4.4584	278.09	0.0367	0
7	4.6834	264.73	0.0516	0
8	4.801	258.25	0.4792	0
9	4.9042	252.81	0.1914	0
10	5.1985	238.5	0.1425	0

Table S36. Singlet excited states of **m3** extracted from the **TBDMS-aceAN** crystal with singlet spin.

Excited State	Energy (eV)	λ (nm)	Oscillator Strength	<S**2>
1	2.5021	495.52	0.1097	0
2	3.1219	397.14	0.2294	0
3	3.7804	327.97	0.1496	0
4	4.3221	286.86	0.0001	0
5	4.4336	279.65	0.0119	0
6	4.4584	278.09	0.0367	0
7	4.6834	264.73	0.0516	0
8	4.801	258.25	0.4792	0
9	4.9042	252.81	0.1914	0
10	5.1985	238.5	0.1425	0

Table S37. Singlet excited states of **d12** extracted from the **TBDMS-aceAN** crystal with singlet spin.

Excited State	Energy (eV)	λ (nm)	Oscillator Strength	<S**2>
1	2.302	538.6	0	0
2	2.56	484.31	0.1589	0
3	2.9096	426.12	0.1675	0
4	3.0234	410.08	0	0
5	3.1048	399.33	0.1792	0
6	3.154	393.11	0	0
7	3.4343	361.02	0	0
8	3.6073	343.71	0.0906	0
9	3.6976	335.31	0	0
10	3.7862	327.46	0.1692	0

Table S38. Singlet excited states of **d23** extracted from the **TBDMS-aceAN** crystal with singlet spin.

Excited State	Energy (eV)	λ (nm)	Oscillator Strength	<S**2>
1	2.4732	501.3	0	0
2	2.4826	499.41	0.1947	0
3	2.9996	413.34	0	0
4	3.0235	410.07	0.077	0
5	3.0646	404.56	0	0
6	3.151	393.48	0.3204	0
7	3.6673	338.08	0	0
8	3.6908	335.93	0.0883	0
9	3.7957	326.65	0	0
10	3.8103	325.39	0.128	0

Table S39: Triplet excited states of **m1** extracted from the **TBDMS-aceAN** crystal with singlet spin.

Excited State	Energy (eV)	λ (nm)	Oscillator Strength	<S**2>
1	1.1986	1034.42	0	2
2	1.6208	764.95	0	2
3	2.9771	416.46	0	2
4	3.1019	399.71	0	2
5	3.7255	332.8	0	2
6	3.9253	315.86	0	2
7	4.1284	300.32	0	2
8	4.1775	296.79	0	2
9	4.2361	292.68	0	2
10	4.2936	288.77	0	2

Table S40. Triplet excited states of **m2** extracted from the **TBDMS-aceAN** crystal with singlet spin.

Excited State	Energy (eV)	λ (nm)	Oscillator Strength	<S**2>
1	1.1986	1034.42	0	2
2	1.6208	764.96	0	2
3	2.9771	416.46	0	2
4	3.1019	399.71	0	2
5	3.7255	332.8	0	2
6	3.9253	315.86	0	2
7	4.1284	300.32	0	2
8	4.1775	296.79	0	2
9	4.2361	292.68	0	2
10	4.2936	288.77	0	2

Table S41. Triplet excited states of **m3** extracted from the **TBDMS-aceAN** crystal with singlet spin.

Excited State	Energy (eV)	λ (nm)	Oscillator Strength	<S**2>
1	1.1986	1034.42	0	2
2	1.6208	764.95	0	2
3	2.9771	416.46	0	2
4	3.1019	399.71	0	2
5	3.7255	332.8	0	2
6	3.9253	315.86	0	2
7	4.1284	300.32	0	2
8	4.1775	296.79	0	2
9	4.2361	292.68	0	2
10	4.2936	288.77	0	2

Table S42. Triplet excited states of **d12** extracted from the **TBDMS-aceAN** crystal with singlet spin.

Excited State	Energy (eV)	λ (nm)	Oscillator Strength	<S**2>
1	1.1852	1046.13	0	2
2	1.2349	1004.04	0	2
3	1.5951	777.27	0	2
4	1.6436	754.35	0	2
5	2.7946	443.66	0	2
6	2.8249	438.9	0	2
7	3.0002	413.26	0	2
8	3.0749	403.22	0	2
9	3.0987	400.12	0	2
10	3.1509	393.48	0	2

Table S43. Triplet excited states of **d23** extracted from the **TBDMS-aceAN** crystal with singlet spin.

Excited State	Energy (eV)	λ (nm)	Oscillator Strength	<S**2>
1	1.2002	1033	0	2
2	1.2092	1025.37	0	2
3	1.6233	763.76	0	2
4	1.6441	754.12	0	2
5	2.8123	440.86	0	2
6	2.8138	440.63	0	2
7	3.0759	403.08	0	2
8	3.0788	402.7	0	2
9	3.1488	393.75	0	2
10	3.1723	390.83	0	2

Table S44. Singlet excited states of **m1** extracted from the **TES-aceTN** crystal with singlet spin.

Excited State	Energy (eV)	λ (nm)	Oscillator Strength	<S**2>
1	2.1517	576.21	0.0535	0
2	2.3958	517.51	0.2046	0
3	3.4447	359.93	0.2217	0
4	3.9523	313.7	0.0014	0
5	4.2109	294.43	0.0002	0
6	4.3515	284.92	0.0001	0
7	4.4131	280.95	0.0328	0
8	4.489	276.2	0.1044	0
9	4.5927	269.96	0.0105	0
10	4.6998	263.81	0.2857	0

Table S45. Singlet excited states of **m2** extracted from the **TES-aceTN** crystal with singlet spin.

Excited State	Energy (eV)	λ (nm)	Oscillator Strength	<S**2>
1	2.1517	576.21	0.0535	0
2	2.3958	517.51	0.2046	0
3	3.4447	359.93	0.2217	0
4	3.9523	313.7	0.0014	0
5	4.2109	294.43	0.0002	0
6	4.3515	284.92	0.0001	0
7	4.4131	280.95	0.0328	0
8	4.489	276.2	0.1044	0
9	4.5927	269.96	0.0106	0
10	4.6998	263.81	0.2857	0

Table S46. Singlet excited states of **d12** extracted from the **TES-aceTN** crystal with singlet spin.

Excited State	Energy (eV)	λ (nm)	Oscillator Strength	<S**2>
1	1.99	623.03	0.0719	0
2	2.0423	607.08	0.0004	0
3	2.1769	569.55	0.0005	0
4	2.3539	526.71	0.2392	0
5	2.6096	475.11	0.0023	0
6	2.7164	456.42	0.1246	0
7	2.8919	428.72	0.0098	0
8	2.9299	423.16	0.004	0
9	3.1874	388.98	0.0003	0
10	3.4638	357.94	0.2591	0

Table S47. Triplet excited states of **m1** extracted from the **TES-aceTN** crystal with singlet spin.

Excited State	Energy (eV)	λ (nm)	Oscillator Strength	<S**2>
1	0.7479	1657.78	0	2
2	1.0942	1133.12	0	2
3	2.5567	484.94	0	2
4	2.7891	444.53	0	2
5	3.2284	384.04	0	2
6	3.5817	346.16	0	2
7	3.8039	325.94	0	2
8	3.835	323.3	0	2
9	4.0696	304.66	0	2
10	4.1722	297.17	0	2

Table S48. Triplet excited states of **m2** extracted from the **TES-aceTN** crystal with singlet spin.

Excited State	Energy (eV)	λ (nm)	Oscillator Strength	<S**2>
1	0.7479	1657.76	0	2
2	1.0942	1133.13	0	2
3	2.5567	484.94	0	2
4	2.7891	444.53	0	2
5	3.2284	384.04	0	2
6	3.5817	346.16	0	2
7	3.8039	325.94	0	2
8	3.835	323.3	0	2
9	4.0696	304.66	0	2
10	4.1722	297.17	0	2

Table S49. Triplet excited states of **d12** extracted from the **TES-aceTN** crystal with singlet spin.

Excited State	Energy (eV)	λ (nm)	Oscillator Strength	<S**2>
1	0.7503	1652.52	0	2
2	0.7631	1624.84	0	2
3	1.0859	1141.78	0	2
4	1.1633	1065.83	0	2
5	2.3671	523.79	0	2
6	2.3942	517.86	0	2
7	2.6312	471.21	0	2
8	2.6638	465.45	0	2
9	2.696	459.89	0	2
10	2.77	447.59	0	2

Table S50. Singlet excited states of **m1** extracted from the **TIPS-aceTN** crystal with singlet spin.

Excited State	Energy (eV)	λ (nm)	Oscillator Strength	<S**2>
1	2.1163	585.86	0.0524	0
2	2.3875	519.31	0.2181	0
3	3.4382	360.61	0.2213	0
4	3.9293	315.53	0.0011	0
5	4.1192	300.99	0.0003	0
6	4.2265	293.35	0.0001	0
7	4.3876	282.58	0.0503	0
8	4.4178	280.64	0.1513	0
9	4.5704	271.28	0.0071	0
10	4.6955	264.05	0.3217	0

Table S51. Singlet excited states of **m2** extracted from the **TIPS-aceTN** crystal with singlet spin.

Excited State	Energy (eV)	λ (nm)	Oscillator Strength	<S**2>
1	2.1163	585.86	0.0524	0
2	2.3875	519.31	0.2181	0
3	3.4382	360.61	0.2213	0
4	3.9293	315.54	0.0011	0
5	4.1192	300.99	0.0003	0
6	4.2265	293.35	0.0001	0
7	4.3876	282.58	0.0503	0
8	4.4178	280.65	0.1513	0
9	4.5704	271.28	0.0071	0
10	4.6955	264.05	0.3217	0

Table S52. Singlet excited states of **m3** extracted from the **TIPS-aceTN** crystal with singlet spin.

Excited State	Energy (eV)	λ (nm)	Oscillator Strength	<S**2>
1	2.1583	574.45	0.0565	0
2	2.401	516.39	0.2139	0
3	3.4447	359.93	0.2144	0
4	3.9469	314.13	0.0005	0
5	4.1431	299.26	0.0003	0
6	4.2755	289.99	0.0001	0
7	4.3927	282.25	0.0397	0
8	4.4433	279.04	0.1457	0
9	4.5814	270.63	0.005	0
10	4.695	264.08	0.3185	0

Table S53. Singlet excited states of **m4** extracted from the **TIPS-aceTN** crystal with singlet spin.

Excited State	Energy (eV)	λ (nm)	Oscillator Strength	<S**2>
1	2.1583	574.45	0.0565	0
2	2.401	516.39	0.2139	0
3	3.4447	359.93	0.2144	0
4	3.9469	314.13	0.0005	0
5	4.1431	299.26	0.0003	0
6	4.2755	289.99	0.0001	0
7	4.3927	282.25	0.0397	0
8	4.4433	279.04	0.1457	0
9	4.5814	270.63	0.005	0
10	4.695	264.08	0.3185	0

Table S54. Singlet excited states of **d12** extracted from the **TIPS-aceTN** crystal with singlet spin.

Excited State	Energy (eV)	λ (nm)	Oscillator Strength	<S**2>
1	2.0796	596.19	0.0723	0
2	2.1248	583.5	0	0
3	2.2406	553.34	0	0
4	2.3621	524.88	0.2556	0
5	2.5147	493.03	0	0
6	2.5935	478.06	0.1386	0
7	2.7507	450.74	0	0
8	2.7986	443.02	0.0213	0
9	3.3485	370.27	0	0
10	3.4694	357.36	0.2493	0

Table S55. Singlet excited states of **d23** extracted from the **TIPS-aceTN** crystal with singlet spin.

Excited State	Energy (eV)	λ (nm)	Oscillator Strength	<S**2>
1	2.0215	613.33	0.0519	0
2	2.0976	591.07	0.0219	0
3	2.1672	572.09	0.0003	0
4	2.3512	527.31	0.2663	0
5	2.5985	477.14	0.0722	0
6	2.6998	459.23	0.0537	0
7	2.8506	434.94	0.011	0
8	2.9168	425.07	0.0137	0
9	3.1793	389.98	0.003	0
10	3.4726	357.04	0.2651	0

Table S56. Singlet excited states of **d34** extracted from the **TIPS-aceTN** crystal with singlet spin.

Excited State	Energy (eV)	λ (nm)	Oscillator Strength	<S**2>
1	2.1437	578.37	0	0
2	2.1471	577.46	0.1078	0
3	2.3453	528.65	0.5468	0
4	2.4095	514.57	0	0
5	3.0754	403.15	0	0
6	3.0926	400.9	0.0032	0
7	3.3523	369.85	0	0
8	3.3657	368.38	0.0056	0
9	3.4414	360.28	0	0
10	3.4684	357.46	0.3489	0

Table S57. Triplet excited states of **m1** extracted from the **TIPS-aceTN** crystal with singlet spin.

Excited State	Energy (eV)	λ (nm)	Oscillator Strength	<S**2>
1	0.7469	1660.07	0	2
2	1.0527	1177.75	0	2
3	2.5528	485.68	0	2
4	2.7715	447.35	0	2
5	3.2397	382.71	0	2
6	3.5491	349.34	0	2
7	3.7911	327.04	0	2
8	3.8396	322.91	0	2
9	3.9778	311.69	0	2
10	4.082	303.73	0	2

Table S58. Triplet excited states of **m2** extracted from the **TIPS-aceTN** crystal with singlet spin.

Excited State	Energy (eV)	λ (nm)	Oscillator Strength	<S**2>
1	0.7469	1660.08	0	2
2	1.0527	1177.76	0	2
3	2.5528	485.68	0	2
4	2.7715	447.35	0	2
5	3.2397	382.71	0	2
6	3.5491	349.34	0	2
7	3.7911	327.04	0	2
8	3.8396	322.91	0	2
9	3.9778	311.69	0	2
10	4.082	303.73	0	2

Table S59. Triplet excited states of **m3** extracted from the **TIPS-aceTN** crystal with singlet spin.

Excited State	Energy (eV)	λ (nm)	Oscillator Strength	<S**2>
1	0.7649	1620.87	0	2
2	1.0886	1138.98	0	2
3	2.5551	485.24	0	2
4	2.7871	444.86	0	2
5	3.2506	381.41	0	2
6	3.5645	347.83	0	2
7	3.8045	325.89	0	2
8	3.8274	323.94	0	2
9	4.0001	309.95	0	2
10	4.1288	300.29	0	2

Table S60. Triplet excited states of **m4** extracted from the **TIPS-aceTN** crystal with singlet spin.

Excited State	Energy (eV)	λ (nm)	Oscillator Strength	<S**2>
1	0.7649	1620.86	0	2
2	1.0886	1138.98	0	2
3	2.5551	485.24	0	2
4	2.7871	444.86	0	2
5	3.2506	381.41	0	2
6	3.5645	347.83	0	2
7	3.8045	325.88	0	2
8	3.8274	323.94	0	2
9	4.0001	309.95	0	2
10	4.1288	300.29	0	2

Table S61. Triplet excited states of **d12** extracted from the **TIPS-aceTN** crystal with singlet spin.

Excited State	Energy (eV)	λ (nm)	Oscillator Strength	<S**2>
1	0.7473	1659.12	0	2
2	0.8031	1543.79	0	2
3	1.1034	1123.62	0	2
4	1.137	1090.41	0	2
5	2.2692	546.39	0	2
6	2.3289	532.36	0	2
7	2.6276	471.86	0	2
8	2.6676	464.78	0	2
9	2.7113	457.28	0	2
10	2.7426	452.07	0	2

Table S62. Triplet excited states of **d23** extracted from the **TIPS-aceTN** crystal with singlet spin.

Excited State	Energy (eV)	λ (nm)	Oscillator Strength	<S**2>
1	0.7595	1632.51	0	2
2	0.7895	1570.32	0	2
3	1.0835	1144.32	0	2
4	1.1512	1077.03	0	2
5	2.3409	529.65	0	2
6	2.4062	515.26	0	2
7	2.5998	476.91	0	2
8	2.6734	463.77	0	2
9	2.712	457.17	0	2
10	2.7642	448.53	0	2

Table S63. Triplet excited states of **d34** extracted from the **TIPS-aceTN** crystal with singlet spin.

Excited State	Energy (eV)	λ (nm)	Oscillator Strength	<S**2>
1	0.7629	1625.21	0	2
2	0.777	1595.78	0	2
3	1.082	1145.91	0	2
4	1.084	1143.74	0	2
5	2.5463	486.92	0	2
6	2.5508	486.06	0	2
7	2.7907	444.28	0	2
8	2.7933	443.86	0	2
9	3.0653	404.47	0	2
10	3.0697	403.9	0	2

References

(1) Tarleton, A. S.; Garcia-Alvarez, J. C.; Wynn, A.; Awbrey, C. M.; Roberts, T. P.; Gozem, S. OS100: A Benchmark Set of 100 Digitized UV–Visible Spectra and Derived Experimental Oscillator Strengths. *J. Phys. Chem. A* **2022**, *126* (3), 435-443. DOI: 10.1021/acs.jpca.1c08988.

(2) Strickler, S. J.; Berg, R. A. Relationship between Absorption Intensity and Fluorescence Lifetime of Molecules. *J. Chem. Phys.* **1962**, *37* (4), 814. DOI: 10.1063/1.1733166.

(3) Kristoffersen, A. S.; Erga, S. R.; Hamre, B.; Frette, Ø. Testing Fluorescence Lifetime Standards Using Two-Photon Excitation and Time-Domain Instrumentation: Rhodamine B, Coumarin 6 and Lucifer Yellow. *J. Fluoresc.* **2014**, *24* (4), 1015-1024. DOI: 10.1007/s10895-014-1368-1.

(4) Wypych, A. W., George. *Databook of Solvents*; ChemTec Publishing, 2024.

(5) Stern, H. L.; Musser, A. J.; Gelinas, S.; Parkinson, P.; Herz, L. M.; Bruzek, M. J.; Anthony, J.; Friend, R. H.; Walker, B. J. Identification of a Triplet Pair Intermediate in Singlet Exciton Fission in Solution. *Proc. Natl. Acad. Sci.* **2015**, *112* (25), 7656-7661. DOI: 10.1073/pnas.1503471112.

(6) Van Schenck, J. D. B.; Mayonado, G.; Anthony, J. E.; Graham, M. W.; Ostroverkhova, O. Molecular Packing-Dependent Exciton Dynamics in Functionalized Anthradithiophene Derivatives: From Solutions to Crystals. *J. Chem. Phys.* **2020**, *153* (16). DOI: 10.1063/5.0026072.

(7) Thompson, N. J.; Hontz, E.; Chang, W.; Van Voorhis, T.; Baldo, M. Magnetic Field Dependence of Singlet Fission in Solutions of Diphenyl Tetracene. *Philos. Trans. R. Soc. A* **2015**, *373* (2044), 20140323. DOI: 10.1098/rsta.2014.0323.

(8) Yong, C. K.; Musser, A. J.; Bayliss, S. L.; Lukman, S.; Tamura, H.; Bubnova, O.; Hallani, R. K.; Meneau, A.; Resel, R.; Maruyama, M.; Hotta, S.; Herz, L. M.; Beljonne, D.; Anthony, J. E.; Clark, J.; Sirringhaus, H. The Entangled Triplet Pair State in Acene and Heteroacene Materials. *Nat. Commun.* **2017**, *8* (1), 15953. DOI: 10.1038/ncomms15953.

(9) Thomas, K. G.; Biju, V.; George, M. V.; Guldi, D. M.; Kamat, P. V. Excited-State Interactions in Pyrrolidinofullerenes. *J. Phys. Chem. A* **1998**, *102* (28), 5341-5348. DOI: 10.1021/jp972756z.

(10) Maeder, M. N., Yorck-Michael. *Practical Data Analysis in Chemistry*; Elsevier, 2007.

(11) Cruz, C. D.; Stephenson, J. C.; Wahlstrand, J. K. Phase-Sensitive Pump-Probe Measurement of the Complex Nonlinear Susceptibility of Silicon Across the Direct Band Edge. *Optica* **2024**, *11* (9), 1313-1319. DOI: 10.1364/OPTICA.530147.

(12) Albert-Seifried, S.; Friend, R. H. Measurement of Thermal Modulation of Optical Absorption in Pump-Probe Spectroscopy of Semiconducting Polymers. *Appl. Phys. Lett.* **2011**, *98* (22). DOI: 10.1063/1.3595340.

(13) Rao, A.; Wilson, M. W. B.; Albert-Seifried, S.; Di Pietro, R.; Friend, R. H. Photophysics of Pentacene Thin Films: The Role of Exciton Fission and Heating Effects. *Phys. Rev. B* **2011**, *84* (19), 195411. DOI: 10.1103/PhysRevB.84.195411.

(14) Le, A. K.; Bender, J. A.; Roberts, S. T. Slow Singlet Fission Observed in a Polycrystalline Perylenediimide Thin Film. *J. Phys. Chem. Lett.* **2016**, *7* (23), 4922-4928. DOI: 10.1021/acs.jpclett.6b02320.

(15) Panthi, Y. R.; Thottappali, M. A.; Horáková, P.; Kubáč, L.; Pfleger, J.; Menšík, M.; Khan, T. Photophysics of Benzoxazole and Dicyano Functionalised Diketopyrrolopyrrole Derivatives: Insights into Ultrafast Processes and the Triplet State. *ChemPhysChem* **2024**, *25* (13), e202300872. DOI: 10.1002/cphc.202300872.

(16) Fulem, M.; Laštovka, V.; Straka, M.; Růžička, K.; Shaw, J. M. Heat Capacities of Tetracene and Pentacene. *J. Chem. Eng. Data* **2008**, *53* (9), 2175-2181. DOI: 10.1021/je800382b.

(17) *CRC Handbook of Chemistry and Physics*; CRC Press/Taylor & Francis, 2018.

(18) Carslaw, H. S.; Jaeger, J. C. *Conduction of Heat in Solids*; Clarendon Press, 1947.

(19) Zhang, H.; Brill, J. W. Interlayer Thermal Conductivity of Rubrene Measured by AC-Calorimetry. *J. Appl. Phys.* **2013**, *114* (4). DOI: 10.1063/1.4816468.

(20) Okada, Y.; Uno, M.; Nakazawa, Y.; Sasai, K.; Matsukawa, K.; Yoshimura, M.; Kitaoka, Y.; Mori, Y.; Takeya, J. Low-Temperature Thermal Conductivity of Bulk and Film-Like Rubrene Single Crystals. *Phys. Rev. B* **2011**, *83* (11), 113305. DOI: 10.1103/PhysRevB.83.113305.

(21) Epstein, J.; Ong, W.-L.; Bettinger, C. J.; Malen, J. A. Temperature Dependent Thermal Conductivity and Thermal Interface Resistance of Pentacene Thin Films with Varying Morphology. *ACS Appl. Mater. Interfaces* **2016**, *8* (29), 19168-19174. DOI: 10.1021/acsami.6b06338.

(22) Zhang, H.; Yao, Y.; Payne, M. M.; Anthony, J. E.; Brill, J. W. Thermal Diffusivities of Functionalized Pentacene Semiconductors. *Appl. Phys. Lett.* **2014**, *105* (7). DOI: 10.1063/1.4894094.

(23) Yao, Y.; Shahi, M.; Payne, M. M.; Anthony, J. E.; Brill, J. W. Thermal Resistances of Thin Films of Small Molecule Organic Semiconductors. *J. Mater. Chem. C* **2016**, *4* (37), 8817-8821. DOI: 10.1039/C6TC03569H.
